# A selective WDR5 degrader inhibits acute myeloid leukemia in patient-derived mouse models

**DOI:** 10.1126/scitranslmed.abj1578

**Published:** 2021-09-29

**Authors:** Xufen Yu, Dongxu Li, Jithesh Kottur, Yudao Shen, Huen Suk Kim, Kwang-Su Park, Yi-Hsuan Tsai, Weida Gong, Jun Wang, Kyogo Suzuki, Joel Parker, Laura Herring, H. Ümit Kaniskan, Ling Cai, Rinku Jain, Jing Liu, Aneel K Aggarwal, Gang Greg Wang, Jian Jin

**Affiliations:** 1 Mount Sinai Center for Therapeutics Discovery, Icahn School of Medicine at Mount Sinai, New York, NY 10029, USA.; 2 Departments of Pharmacological Sciences and Oncological Sciences, Tisch Cancer Institute, Icahn School of Medicine at Mount Sinai, New York, NY 10029, USA.; 3 Lineberger Comprehensive Cancer Center, University of North Carolina at Chapel Hill, Chapel Hill, NC 27599, USA.; 4 Department of Biochemistry and Biophysics, University of North Carolina at Chapel Hill, Chapel Hill, NC 27599, USA.; 5 Department of Genetics, University of North Carolina at Chapel Hill, Chapel Hill, NC 27599, USA.; 6 Department of Pharmacology, University of North Carolina at Chapel Hill, Chapel Hill, NC 27599, USA.

## Abstract

Interactions between WD40 repeat domain protein 5 (WDR5) and its various partners such as mixed lineage leukemia (MLL) and c-MYC are essential for sustaining oncogenesis in human cancers. However, inhibitors that block protein-protein interactions (PPIs) between WDR5 and its binding partners exhibit modest cancer cell killing effects and lack in vivo efficacy. Here, we present pharmacological degradation of WDR5 as a promising therapeutic strategy for treating WDR5-dependent tumors and report two high-resolution crystal structures of WDR5-degrader-E3 ligase ternary complexes. We identified an effective WDR5 degrader via structure-based design and demonstrated its in vitro and in vivo antitumor activities. On the basis of the crystal structure of an initial WDR5 degrader in complex with WDR5 and the E3 ligase von Hippel–Lindau (VHL), we designed a WDR5 degrader, MS67, and demonstrated the high cooperativity of MS67 binding to WDR5 and VHL by another ternary complex structure and biophysical characterization. MS67 potently and selectively depleted WDR5 and was more effective than WDR5 PPI inhibitors in suppressing transcription of WDR5-regulated genes, decreasing the chromatin-bound fraction of MLL complex components and c-MYC, and inhibiting the proliferation of cancer cells. In addition, MS67 suppressed malignant growth of MLL-rearranged acute myeloid leukemia patient cells in vitro and in vivo and was well tolerated in vivo. Collectively, our results demonstrate that structure-based design can be an effective strategy to identify highly active degraders and suggest that pharmacological degradation of WDR5 might be a promising treatment for WDR5-dependent cancers.

## INTRODUCTION

The chromatin-associated WD40 repeat domain protein 5 (WDR5) acts as a functional subunit of the mixed lineage leukemia (MLL) histone methyltransferase complexes (1–3). WDR5 is critical for the methylation of histone H3 lysine 4 (H3K4) on chromatin catalyzed by the MLL1 complex and MLL1 complex–mediated regulations of gene transcription (1–3). WDR5 adopts a donut-shaped propeller structure, containing a WDR5 interaction (WIN) motif that binds MLL1, and another cleft known as the WDR5 binding motif (WBM) site that mediates protein-protein interactions (PPIs) with a diverse set of non-MLL partners such as c-MYC (3, 4). WDR5 contributes to tumorigenesis in a wide range of human cancers. In acute myeloid leukemia (AML) harboring MLL rearrangement (MLL-r AML), the complex assembled by WDR5 and wild-type (WT) MLL cooperates with the MLL-r chimeric oncoproteins to sustain an oncogenic gene expression program, and depletion of the WDR5-MLL1 complex suppresses the growth of MLL-r AMLs (5–7). Furthermore, WDR5 has been found to be overexpressed in a number of solid tumors including pancreatic ductal adenocarcinoma (PDAC), promoting oncogenesis (8–15). A WDR5-MYC axis was shown to be critically involved in tumorigenesis in pancreatic cancer, neuroblastoma, and Burkitt’s lymphoma (8, 15–17). Therefore, targeting WDR5-directed gene regulatory activities represents an attractive strategy for therapeutic interventions in both hematological and solid tumors.

Substantial progress has been made on generating inhibitors that block the binding of WDR5 to its partners by targeting the WIN and WBM binding sites, respectively (18–31). Both cyclic peptidomimetic inhibitors such as MM-401 (20, 21, 26) and small-molecule inhibitors such as OICR-9429 (18, 19, 24, 25, 27, 28, 30) have been developed to disrupt WDR5-MLL1 PPIs by occupying the WIN motif. Recently, small-molecule inhibitors that bind the WBM binding site and block WDR5-MYC interactions have also been reported (29, 31). Some of these WDR5 PPI inhibitors have been shown to exert antiproliferative effects in cancer cells. For example, OICR-9429 selectively kills AML cells that express p30, a mutated form of the transcription factor C/EBPα, and reduces the proliferation of cancer cells carrying TP53 gain-of-function mutations (22, 23). MM-401 induces myeloid differentiation of MLL cells (21). However, these inhibitors that block PPIs between WDR5 and its binding partners in general exhibit only partial or modest effects on cancer cells and are not efficacious in vivo in preclinical cancer models. The relatively weak antitumor activities are likely due to the fact that (i) these WDR5 PPI inhibitors, which rely on receptor occupancy pharmacology, do not achieve full and durable blockade of the interactions between WDR5 and its partners and (ii), more importantly, these PPI inhibitors target only some but not all of WDR5’s oncogenic functions, such as its interaction with MLL1 via the WIN motif and that with c-MYC via the WBM site. Thus, a new therapeutic strategy that can achieve complete and sustained blockage all of WDR5’s multifaceted oncogenic functions in tumor is desirable.

Proteolysis-targeting chimeras (PROTACs) have recently emerged as promising therapeutic modalities (32–34). PROTACs simultaneously bind the protein of interest (POI) and an E3 ligase such as von Hippel–Lindau (VHL) or cereblon (CRBN) and hijack the cellular ubiquitination-proteasome system, leading to selective polyubiquitination and subsequent degradation of the POI at the proteasome. In contrast to small-molecule inhibitors that rely on receptor occupancy pharmacology and do not typically target multiple functions of the POI, PROTACs pharmacologically deplete the POI, thus temporally eliminating all functions of the POI. Moreover, the catalytic nature of PROTACs potentially reduces the need for high drug residence time and continuous drug exposure.

Here, we report the development of a WDR5 PROTAC degrader. We present the high-resolution crystal structures of the WDR5-degrader-VHL ternary complexes, the structure-based design exploiting the initial ternary complex structure that led to a highly effective WDR5 degrader, MS67, and a thorough characterization of MS67 in a battery of biochemical, biophysical, structural, genomic, cellular, and in vivo studies. Our results demonstrate that MS67 offers a potential therapeutic avenue for WDR5-dependent cancers.

## RESULTS

### Discovery and biochemical characterization of an initial WDR5 degrader, MS33

**Fig. 1.**
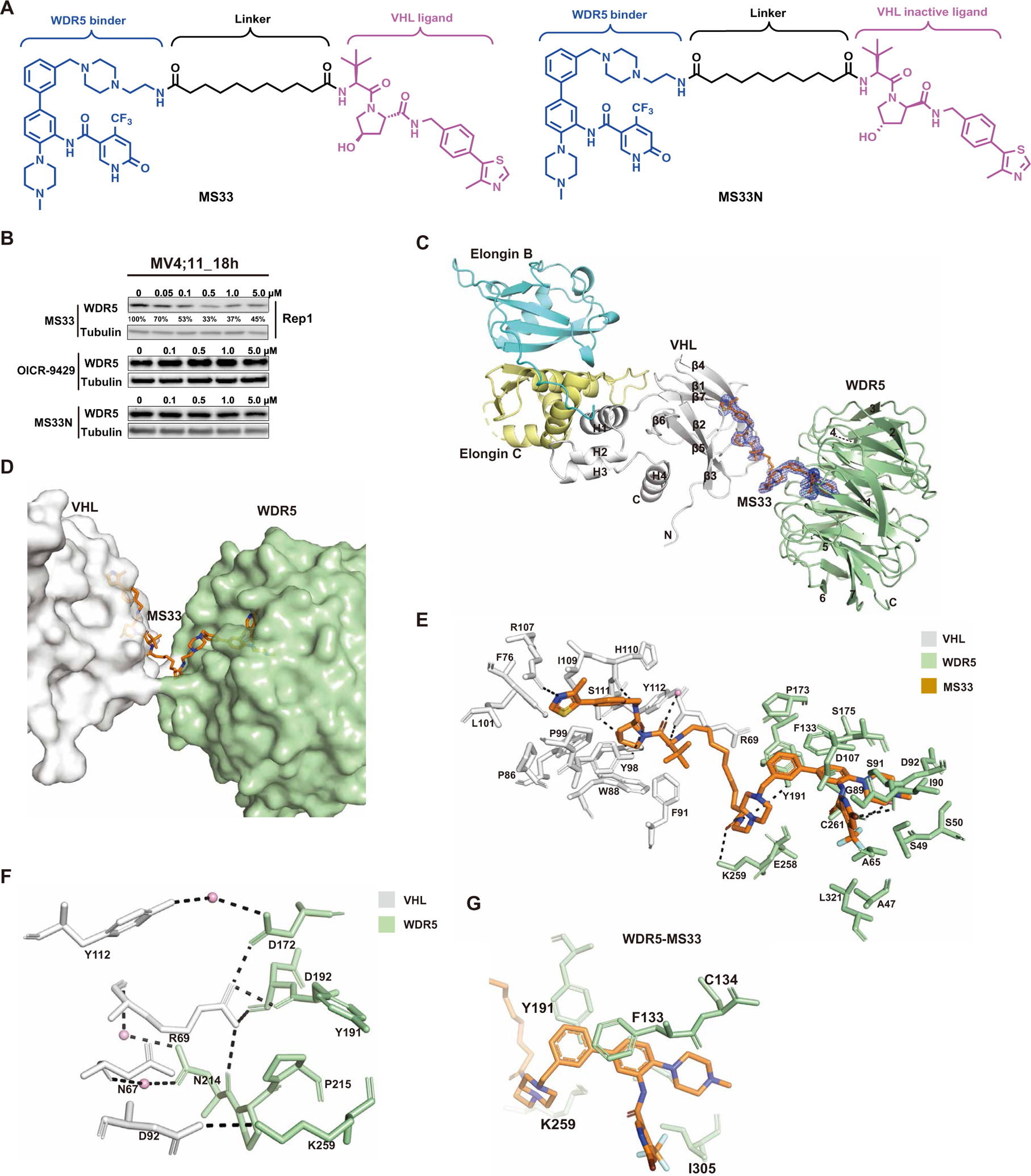
Design of WDR5 degrader MS33 and the crystal structure of the VCB-MS33-WDR5 ternary complex. (**A**) Chemical structures of WDR5 degrader MS33 and a negative control of MS33, MS33N. (**B**) Immunoblots for WDR5 and Tubulin posttreatment of MV4;11 cells with the indicated concentrations of MS33, OICR-9429, or MS33N for 18 hours. (**C**) Overall structure of the VCB-MS33-WDR5 ternary complex displayed in ribbon representation with VHL, Elongin C, Elongin B, and WDR5 colored in gray, pale yellow, cyan, and pale green, respectively. The secondary structure elements for VHL are labeled. The seven β-propellers of WDR5 are also labeled. The simulated annealing *F*_o_-*F*_c_ omit map (blue mesh) for MS33 is displayed (contoured at 3.0σ with a carve radius of 2.0 Å). (**D**) Overview of the VHL-MS33-WDR5 ternary complex, with VHL, WDR5, and MS33 shown in gray, pale green, and orange, respectively. (**E**) Detailed view of the binding interactions of MS33 with VHL (gray) and WDR5 (pale green) in the VCB-MS33-WDR5 complex. Only amino acids within 4-Å spheres of MS33 are depicted. Water molecules are depicted as pink spheres. Hydrogen bonds are depicted by dashed lines. (**F**) VHL-WDR5 interface in the VCB-MS33-WDR5 complex. The key amino acids participating in interactions at the interface between VHL and WDR5 in the VCB-MS33-WDR5 complex are shown. Arg^69^ exists in two conformations, and only one conformation is shown for clarity. Water molecules are depicted as pink spheres. Hydrogen bonds are depicted by dashed lines. (**G**) Close-up view of contacts between the WDR5 binding moiety of MS33 (in orange) and WDR5 residues (in pale green) in the VCB-MS33-WDR5 ternary complex.

We selected OICR-9429 as the WDR5 binding moiety for generating WDR5 degraders, because OICR-9429 is a well-characterized small-molecule inhibitor of WDR5 with high binding affinity (*K*_i_ = 64 nM) (fig. S1A). Upon inspecting the previously published crystal structure of OICR-9429 in complex with WDR5 [Protein Data Bank (PDB) ID: 4QL1] (24), we hypothesized that, although most of OICR-9429’s morpholine ring is missing in the cocrystal structure, this moiety is solvent-exposed on the basis of the limited electron density observed (fig. S1B). We therefore selected this moiety as a linker attachment point and replaced the morpholine group with piperazine tethered with a short ethylamine group as an exit vector for linking with an E3 ligase ligand. We synthesized heterobifunctional compounds by conjugating this modified OICR-9429–based WDR5-binding moiety to VHL and CRBN ligands via a variety of linkers (fig. S1C). Through immunoblotting analysis of these compounds (fig. S1D), we identified MS33 as an early WDR5 degrader lead, which contains the E3 ligase ligand VHL-1 (35) and a relatively long linker (Fig. 1A). We also developed MS33N, a close analog of MS33, which contains the same WDR5-binding moiety and linker, but a diastereoisomer of VHL-1 that is incapable of binding the VHL E3 ligase (36), as a control for MS33 (Fig. 1A).

We next assessed the effect of MS33 on WDR5 degradation in MV4;11 cells, a human AML cell line harboring MLL-r. We found that MS33, but not OICR-9429 or MS33N, induced WDR5 degradation in a concentration-dependent manner with a slight hook effect, a common phenomenon that some PROTACs are less effective in degrading the target protein at higher concentrations due to the formation of unproductive binary complexes (Fig. 1B and fig. S2A) (36). The half-maximal degradation concentration (DC_50_) of MS33 was 260 ± 56 nM with the maximum degradation (*D*_max_) of 71 ± 5% (Fig. 1B and fig. S2, A and B). MS33, but not OICR-9429, also induced WDR5 degradation in MV4;11 cells in a time-dependent manner with apparent degradation detected as early as 4 hours and maximal degradation at around 16 hours (fig. S2C).

We next determined the mechanism of action (MOA) of MS33-induced WDR5 degradation. Pretreatment of MV4;11 cells with OICR-9429 suppressed MS33-mediated WDR5 degradation in a concentration-dependent manner (fig. S2D). In addition, MS33-induced WDR5 degradation was effectively blocked by pretreatment of MV4;11 cells with the proteasome inhibitor carfilzomib, neddylation inhibitor MLN4924, or VHL ligands acetyl VHL-1 (Ac-VHL) and acetyl-capped methylated VHL-1 (Ac-VHL-Me) (fig. S2, D and E) (34, 37). As expected, Ac-VHL, a ligand with lower VHL-binding affinity than Ac-VHL-Me, was not as effective as Ac-VHL-Me in blocking MS33-induced WDR5 degradation. Furthermore, compared to concentration-dependent WDR5 degradation in WT 293FT cells with a hook effect observed at 5 μM, CRISPR-Cas9–mediated knockout (KO) of VHL in 293FT cells abrogated MS33-mediated WDR5 degradation (fig. S2F). Collectively, these results demonstrate that MS33 induced WDR5 degradation in a concentration, time, WDR5, E3 ligase VHL, and proteasome-dependent manner.

### Structural and biophysical characterization of the WDR5-MS33-VCB ternary complex

To understand the underlying structural mechanism by which MS33 corecruits WDR5 and VCB (VHL–Elongin C–Elongin B ternary complex) to establish a “degrader” complex and to design potentially more effective WDR5 degraders, we solved the crystal structure of the WDR5-MS33-VCB complex at 1.7-Å resolution (PDB ID: 7JTO; table S1). The ternary complex of WDR5-MS33-VCB crystallizes in space group *P*2_1_ with one molecule in the crystallographic asymmetric unit. As anticipated from previous structural studies, WDR5 is composed of a seven-blade β-propeller structure, wherein the blades are arranged around a pseudo-symmetry axis, and with a channel running through the middle of the β-propeller structure. The “top” side of the β-propeller structure contains the WBM site, whereas the “bottom” face has the WIN motif. VHL of VCB is composed of a larger β domain, which is composed predominantly of β sheets, that binds to a peptide segment of hypoxia-inducible factor–1α (HIF-1α) and smaller α domain, composed of α helices, that makes the majority of contacts to Elongin C (Fig. 1C).

The electron density for MS33 is well defined and bridges WDR5 and VHL (Fig. 1C). The VHL-1 and OICR-9429 moieties of MS33 fit into the HIF-1α–binding pocket of VHL and the WIN cavity of WDR5, respectively (Fig. 1, C to E), and interacted in an almost identical manner as described in previous binary structures (PDB IDs: 6GFY and 4QL1) (24, 38). One key difference was that, whereas the electron density for most of the morpholine ring of OICR-9429 was missing in the OICR-9429-WDR5 binary complex, suggestive of multiple conformations, electron density for the structurally equivalent piperazine ring in MS33 was ordered, wherein the ring protruded out of the WIN cavity and connected to the linker (Fig. 1, C and E). Overall, the linker in MS33 was relatively extended and resulted in a sparse protein-protein interface between VHL and WDR5 (Fig. 1, C to E). The most prominent of protein-protein contacts involved Arg^69^ of VHL, in one of its two conformations, making hydrogen bonds and electrostatic interactions with the side chain of Asp^172^ and the main chain carbonyl atoms of Tyr^191^, Asp^192^, and Asn^214^ of WDR5, as well as electrostatic interactions between Asp^92^ of VHL and Lys^259^ of WDR5 (Fig. 1F). Many water molecules permeated the VHL-WDR5 interface, with several molecules mediating contacts between VHL and WDR5 (Fig. 1F). Because of the relatively large separation between VHL and WDR5, there were almost no “cross” protein-ligand interactions. That is, VHL interacted exclusively with the VHL-1 portion of MS33 and WDR5 interacted exclusively with the OICR-9429 portion (Fig. 1E).

To assess the effect of the VHL-WDR5 protein-protein contacts on the cooperativity of the WDR5-MS33-VCB ternary complex, we used isothermal titration calorimetry (ITC). The experimental strategy was similar to that previously described (39) in which WDR5 was first titrated against MS33 to saturation and then VCB was titrated into the saturated WDR5-MS33 complex, forming the WDR5-MS33-VCB ternary complex (fig. S3). Titration of VCB into MS33 was used as a reference. We observed an enhancement in VCB binding to a preformed WDR5-MS33 binary complex (α = *K*_d_(binary)/*K*_d_(ternary) = 1.66), reflecting that the VHL and WDR5 interface was stabilized by a few positive interactions (Fig. 1F). The dissociation constant (*K*_d_) values for MS33 binding to VCB and WDR5 were 870 ± 76 nM and 120 ± 7 nM, respectively (fig. S3). We also determined that MS33N bound WDR5 with a similar affinity (*K*_d_ = 86 ± 3.4 nM) as MS33 but did not bind VHL as expected (fig. S4, A and B).

### Structure-based discovery of the WDR5 degrader, MS67

**Fig. 2.**
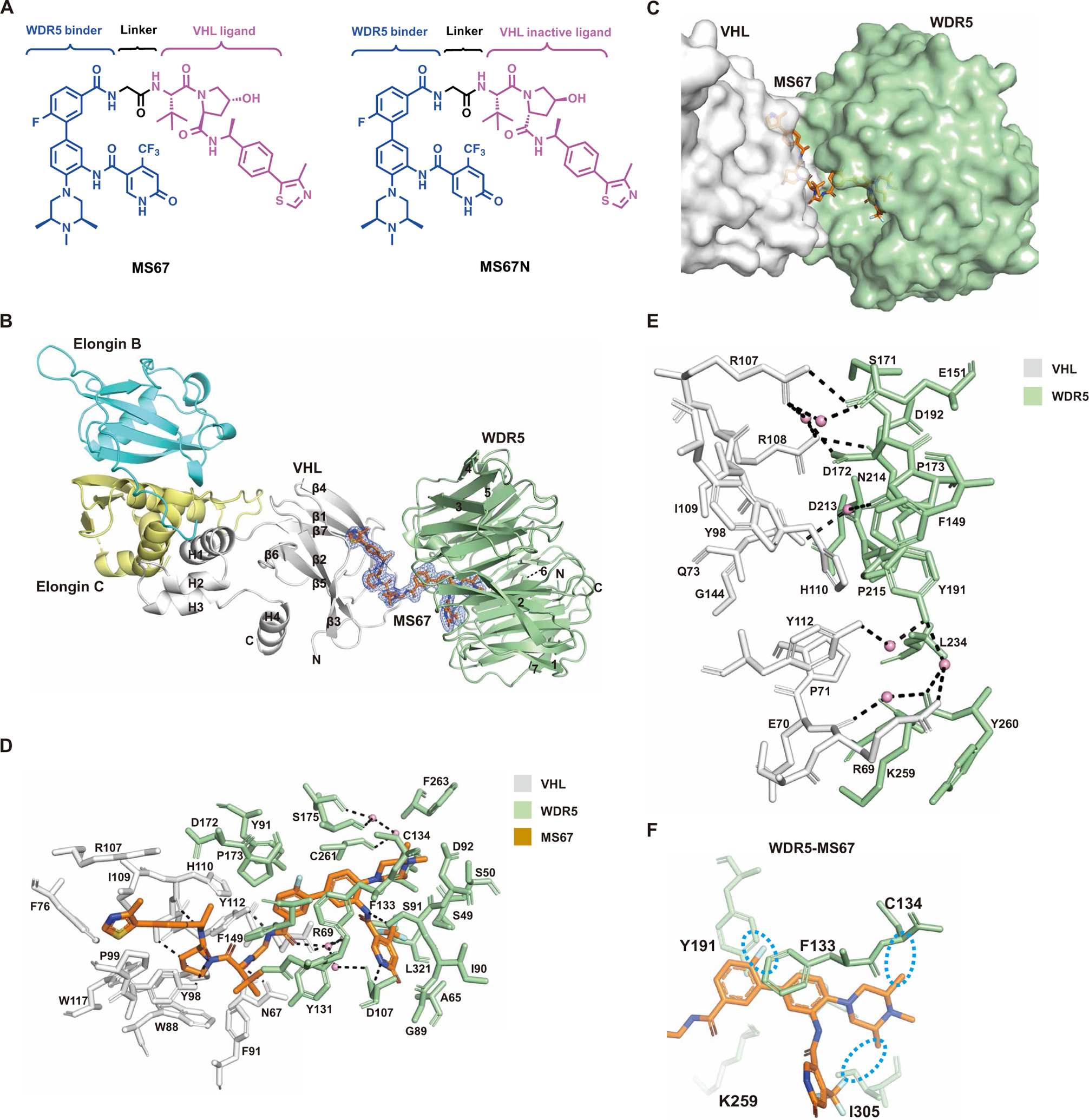
Design of WDR5 degrader MS67 and the crystal structure of the VCB-MS67-WDR5 ternary complex. (**A**) Chemical structures of WDR5 degrader MS67 and a negative control of MS67, MS67N. (**B**) Overall structure of the VCB-MS67-WDR5 ternary complex displayed in ribbon representation with VHL, Elongin C, Elongin B, and WDR5 colored in gray, pale yellow, cyan, and pale green, respectively. The secondary structure elements for VHL are labeled. The seven β-propellers of WDR5 are also labeled. The simulated annealing *F*_o_-*F*_c_ omit map (blue mesh) for MS67 is displayed (contoured at 3.0σ with a carve radius of 2.0 Å). (**C**) Overview of the VHL-MS67-WDR5 ternary complex, with VHL, WDR5, and MS67 shown in gray, pale green, and orange, respectively. (**D**) Detailed view of the binding interactions of MS67 with VHL (gray) and WDR5 (pale green) in the VCB-MS67-WDR5 complex. Only amino acids within 4-Å spheres of MS67 are depicted. Water molecules are depicted as pink spheres. Hydrogen bonds are depicted by dashed lines. (**E**) VHL-WDR5 interface in the VCB-MS67-WDR5 complex. The key amino acids participating in interactions at the interface between VHL and WDR5 in the VCB-MS67-WDR5 complex are shown. Water molecules are depicted as pink spheres. Hydrogen bonds are depicted by dashed lines. (**F**) Close-up view of contacts between the WDR5 binding moiety of MS67 (in orange) and WDR5 residues (in pale green) in the VCB-MS67-WDR5 ternary complex. The cyan dotted circles highlight the newly introduced substituents to the WDR5 binding moiety of MS67.

The crystal structure of the WDR5-MS33-VCB complex provided crucial insights into the WDR5-VHL interface induced by MS33 and protein-ligand interactions and offered a unique opportunity to design more effective WDR5 degraders. We exploited these structural insights and designed a small set of WDR5 degraders to optimize the linker, WDR5 binding moiety, and VHL binding moiety (fig. S5A). On the basis of the WDR5-MS33-VCB ternary structure (Fig. 1, C to E), we reasoned that a shorter linker would juxtapose WDR5 and VHL closer together, allowing for increased protein-protein and cross protein-ligand interactions, which would likely result in enhanced cooperativity of the ternary complex. Thus, we designed two short linkers including a much shorter linker by removing the upper piperazinyl group of MS33 (fig. S5A). We also designed and incorporated moieties that could potentially bind with higher affinities to WDR5 and VHL, respectively. By analyzing the WDR5-MS33-VCB ternary structure, we found that the lower methylpiperazine group of MS33 does not fully occupy the hydrophobic binding cavity of WDR5 (Fig. 1G). The introduction of two methyl substituents at the 2- and 4-position of the methylpiperazine group, respectively, could, in principle, enhance hydrophobic interactions between the ligand and WDR5. Furthermore, the addition of a fluoro substituent to the upper phenyl ring of MS33 could enhance its interactions with nearby Phe^133^ and Tyr^191^ of WDR5 (Fig. 1G). Moreover, it was previously reported that the replacement of the VHL-1 ligand with methylated VHL-1 (VHL-1–Me) could enhance VHL binding and result in more effective degraders (40). We synthesized and evaluated the compounds that contain these chemical modifications alone or in combination (fig. S5, A and B). From this study, we identified MS67 as an effective WDR5 degrader (Fig. 2A). Our structure-activity relationship results (fig. S5) suggested that it was critical to simultaneously shorten the linker and enhance binding affinities to both WDR5 and VHL to generate an effective WDR5 degraders. Last, we also designed MS67N (Fig. 2A), a diastereoisomer of MS67, which contains the identical WDR5 binding moiety and linker but a diastereoisomer of VHL-1–Me to maintain WDR5 binding but abrogate VHL binding, as a negative control of MS67.

We next solved the crystal structure of the WDR5-MS67-VCB complex at 2.1-Å resolution (Fig. 2B), wherein the complex crystallizes in space group *P*2_1_2_1_2_1_ with one complex in the crystallographic asymmetric unit (PDB ID: 7JTP; table S1). As anticipated, MS67 had VHL and WDR5 closer than in the MS33 ternary complex, resulting in a more extensive protein-protein interface (Fig. 2, B to D). The trajectory of the MS67 linker was roughly orthogonal to that of the MS33 linker and created a different VHL-WDR5 interface than that observed in the MS33 ternary complex (Figs. 1, C to E, and 2, B to D; and fig. S6). Overall, WDR5 underwent a large rotation and translation in the direction of the loop between β4 and β5 of VHL (Fig. 2B and fig. S6). Compared to the WDR5-MS33-VCB ternary complex, there was a greater preponderance of nonpolar interactions at the VHL-WDR5 interface, as exemplified by Tyr^191^ and Leu^234^ of WDR5 making nonpolar contacts with His^110^ and Pro^71^ of VHL, respectively (Fig. 2E). Several new hydrogen bonds were also present, including ones between the side chain of WDR5 Asp^172^ and the side chains of VHL Arg^107^ and Arg^108^ (Fig. 2E). Unlike the MS33 ternary complex, there were substantial cross protein-ligand interactions, wherein WDR5 made contacts with the VHL-binding portion of MS67 and VHL made contacts with the WDR5-binding portion of MS67 (Fig. 2D). The methyl and *t*-butyl groups of the VHL-1–Me moiety were involved in hydrophobic contacts with Phe^149^, Pro^173^, and Tyr^131^ of WDR5, and conversely, the fluorobenzyl ring of the WDR5-binding moiety made van der Waals contacts with Tyr^112^ and His^110^ of VHL. Together, the more extensive VHL-WDR5 interface and the cross protein-ligand interaction were expected to increase the cooperativity of the WDR5-MS67-VCB complex. Furthermore, the WDR5-MS67-VCB structure also confirmed our design for enhancing WDR5 binding. The methyl substituents we introduced at the 2- and 4-position of the methyl piperazine group were able to fill in the hydrophobic cavity where the methylpiperazine group sat and the fluoro group added to the phenyl ring interacted with Phe^133^ and Tyr^191^ of WDR5 (Fig. 2F).

**Fig. 3.**
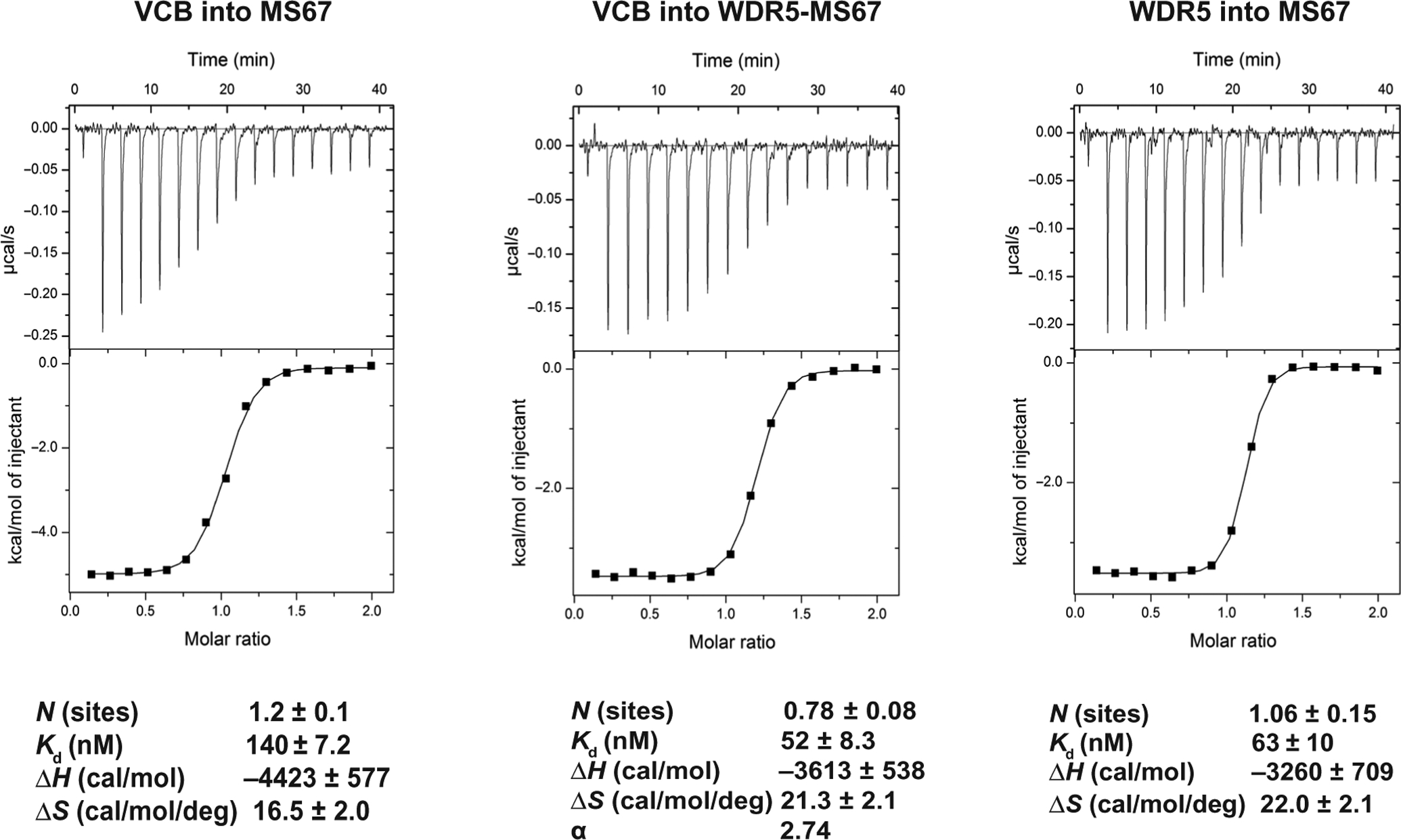
Inverse ITC titrations of VCB into MS67 and MS67-WDR5 complex. Representative inverse ITC titrations are shown for VCB into MS67 (left), VCB into MS67-WDR5 complex (middle), and WDR5 into degrader MS67 (right) for measuring binding kinetic and determining cooperativity (α) for MS67. The calculated values represent the means ± SD from three independent experiments. First injection has been removed from the fitting.

We conducted ITC studies and determined that the binding affinities of MS67 to VCB (*K*_d_ of 140 ± 7.2 nM versus 870 ± 76 nM) and WDR5 (*K*_d_ of 63 ± 10 nM versus 120 ± 7 nM) were indeed improved compared to MS33 (Fig. 3 and figs. S3 and S7). The overall affinity of the WDR5-MS67-VCB ternary complex was a magnitude higher than that for the WDR5-MS33-VCB complex (*K*_d_ of 52 ± 8.3 nM versus 520 ± 34 nM), reflecting enhanced interactions between MS67 and VHL and between MS67 and WDR5. In addition, there was a marked increase in cooperativity between MS67-WDR5 and VCB (α of 2.74 for MS67 versus 1.66 for MS33), reflecting the more extensive VHL-WDR5 interface and cross protein-ligand interactions. Furthermore, we confirmed that MS67N bound WDR5 with high affinity (*K*_d_ = 47 ± 3.4 nM), similar to MS67, but did not bind VHL (fig. S8, A and B). Overall, our structural and biophysical characterization results suggested that MS67 could be a more effective WDR5 degrader than MS33.

### MS67 potently and selectively degrades WDR5 in MLL-r AML and PDAC cells

**Fig. 4.**
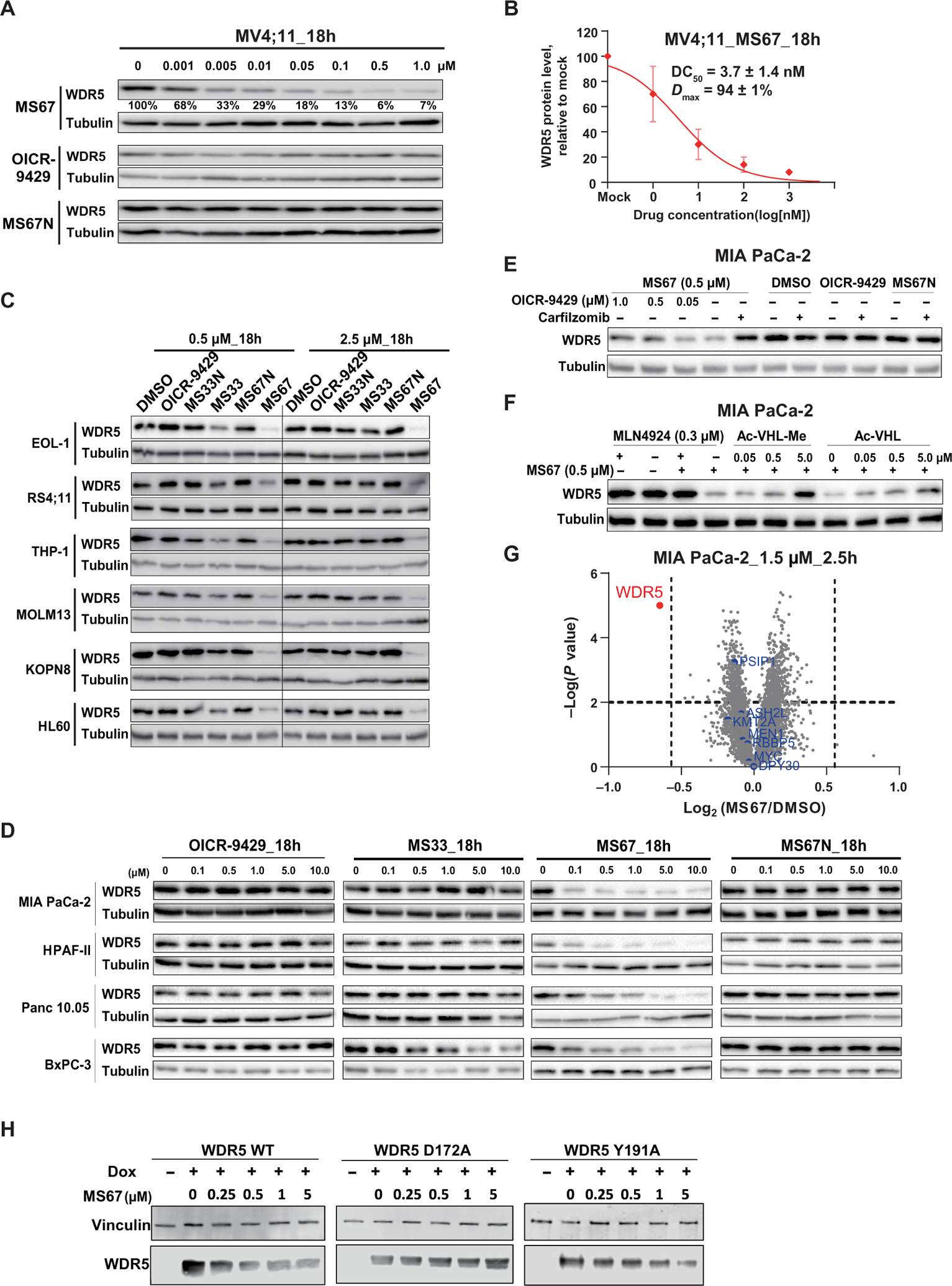
MS67 potently and selectively degrades WDR5 in MLL-r AML and PDAC cells. (**A**) Immunoblots for WDR5 and Tubulin posttreatment of MV4;11 cells with the indicated concentrations of MS67, MS67N, or OICR-9429 for 18 hours. (**B**) DC_50_ and *D*_max_ values of MS67 in MV4;11 cells are shown as the means ± SD from three independent experiments. MV4;11 cells were treated with MS67 for 18 hours. The band intensity is determined by ImageJ software. (**C**) Immunoblots for WDR5 and Tubulin posttreatment of the indicated MLL-r AML cell lines and HL-60 (a non-MLL-r leukemia cell line) with dimethyl sulfoxide (DMSO) and 0.5 or 2.5 μM OICR-9429, MS33, MS33N, MS67, or MS67N for 18 hours. (**D**) Immunoblots for WDR5 and Tubulin posttreatment with the indicated concentrations of OICR-9429, MS33, MS67, or MS67N in the indicated PDAC cell lines for 18 hours. (**E**) Immunoblots for WDR5 and Tubulin after a 2-hour pretreatment with DMSO, carfilzomib (0.4 μM), or OICR-9429 (0.05, 0.5, and 1.0 μM), followed by a 4-hour treatment with 0.5 μM MS67 in MIA PaCa-2 cells. (**F**) Immunoblots for WDR5 and Tubulin after a 2-hour pretreatment with DMSO, MLN4924 (0.3 μM), or Ac-VHL-Me/Ac-VHL (0.05, 0.5, and 5 μM), followed by a 4-hour treatment with MS67 (0.5 μM) in MIA PaCa-2 cells. (**G**) Quantitative proteomics analysis of MIA PaCa-2 cells treated with 1.5 μM MS67 versus DMSO for 2.5 hours. A total of 4039 proteins were identified and quantified. The dashed lines indicate a cutoff of *P* value less than 0.01 (*y* axis) and fold change greater than 1.5 (*x* axis) in three biological replicates. (**H**) The effect of MS67 on degrading WDR5 WT and D172A and Y191A WDR5 mutants. HEK293T cells ectopically overexpressed with WDR5 WT and D172A and Y191A mutants, respectively, upon treatment with doxycycline (Dox) or DMSO, were treated with DMSO or MS67 at indicated concentrations for 72 hours.

We next evaluated the effect of MS67 on degrading WDR5 in human MLL-r AML and PDAC cells, the growth of which were previously shown to be WDR5-dependent (8, 21). We first treated MV4;11 cells with MS67, OICR-9429, or MS67N for 18 hours and found that MS67, but not OICR-9429 and MS67N, induced WDR5 degradation at a concentration as low as 1 nM with DC_50_ of 3.7 ± 1.4 nM and achieved near-complete depletion of WDR5 at 0.5 μM with *D*_max_ of 94 ± 1% (Fig. 4, A and B, and fig. S9A). The DC_50_ value of MS67 was about 70-fold better than that of MS33, and the *D*_max_ value of MS67 (about 94 versus 71% for MS33) was also higher than that of MS33. We also determined that MS67 had a DC_50_ value of 45 ± 16 nM and *D*_max_ value of 85 ± 6% in MIA PaCa-2 cells (fig. S9, B and C).

We further compared the effect of MS33 and MS67 on degrading WDR5 in a large panel of MLL-r AML and PDAC cells. We found that the effect of MS33 on WDR5 degradation was rather restricted to MLL-r AML cells and the effect was minimal in the tested PDAC cells (Fig. 4, C and D). Among the MLL-r AML cells tested, MS33 degraded WDR5 most effectively in MV4;11 (Fig. 1B and fig. S2A) and EOL-1 (Fig. 4C) cells, and less effectively in RS4;11, THP1, MOLM13 and KOPN8 cells with a hook effect at 2.5 μM (Fig. 4C). In contrast, MS67 induced WDR5 depletion much more effectively in all six MLL-r AML and four PDAC cell lines without a hook effect and in a concentration-dependent manner in PDAC cells (Fig. 4, C and D). As expected, MS67N and OICR-9429 were ineffective in degrading WDR5 in MLL-r AML and PDAC cells (Fig. 4, C and D). Furthermore, the WDR5 degradation effect induced by MS67 was time dependent in both MLL-r AML (MV4;11) and PDAC (MIA PaCa-2) cells with apparent degradation occurring as early as 2 hours (fig. S9, D and E). The maximal degradation was achieved at around 4 hours in MV4;11 cells and around 24 hours in MIA PaCa-2 cells. MS67N and OICR-9429 did not degrade WDR5 in this time-course study in MIA PaCa-2 cells (fig. S9E).

Similar to what we observed for MS33, the MS67-induced WDR5 degradation could be rescued by pretreatment of MIA PaCa-2 cells with OICR-9429, carfilzomib, or MLN4924 (Fig. 4, E and F). In addition, pretreatment of MIA PaCa-2 cells with Ac-VHL-Me or Ac-VHL (Fig. 4F) or KO of VHL in 293FT cells (fig. S9F) also reduced MS67-induced WDR5 degradation. Collectively, these results together with the MOA data of MS33 (fig. S2, D to F) demonstrate the VHL E3 ligase, ubiquitin-proteasome system, and WDR5-dependent MOA for these WDR5 degraders. We also performed washout studies and found that the WDR5 protein expression rebounds substantially at ~48 hours and was near fully recovered at ~72 hours after treatment with MS67 in MV4;11 cells (fig. S9G). In MIA PaCa-2 cells, a similar but faster recovery was observed, the WDR5 protein expression rebounded substantially at ~24 hours and was near fully recovered at ~36 hours after treatment with MS67 (fig. S9H). MS67, but not MS67N or OICR-9429, also degraded WDR5 in a concentration-dependent manner in three murine AML cell lines established by Hoxa9 plus Meis1, MLL-AF9, or MLL-ENL (fig. S9I). MS67 was less potent in these murine AML cell lines than in the human AML lines.

To assess selectivity of MS67, we first used a mass spectrometry (MS)–based global proteomic profiling approach and found that of the 4000+ proteins detected, WDR5 was the sole protein showing a significant decrease or increase in protein amount (with a cutoff of *P* value less than 0.01 and fold change greater than 1.5, relative to the mock treatment) in MIA PaCa-2 cells treated with 1.5 μM MS67 for 2.5 hours (Fig. 4G). We next assessed selectivity of MS67 against 22 protein methyltransferases (table S2) and a broad panel of common drug targets including 45 kinases (table S3) and 44 G protein–coupled receptors (GPCR), ion channels, and transporters (table S4). MS67 did not effectively inhibit or bind these 100+ targets except Sigma 2 receptor (exhibited 67 ± 10% binding at 1 μM). We subsequently determined that MS67 had moderate binding affinity (*K*_i_ = 1.0 ± 0.8 μM) to Sigma 2 receptor (fig. S10).

We next conducted a mutagenesis study to determine whether some of the WDR5-VHL interactions revealed by the WDR5-MS67-VCB structure were important for MS67-induced WDR5 degradation. We had determined that WDR5 Asp^172^ formed hydrogen bonds with VHL Arg^107^ and Arg^108^ and that WDR5 Tyr^191^ made nonpolar contacts with VHL His^110^ (Fig. 2E), so we generated inducible stable cell lines that overexpress WDR5, either WT or mutant (D172A or Y191A), upon treatment with doxycycline. We found that MS67 effectively degraded WT WDR5 but did not degrade the WDR5 D172A mutant and was less effective in degrading the WDR5 Y191A mutant in these cell lines (Fig. 4H). These results indicated that at least some of the WDR5-VHL interactions induced by MS67 were important for MS67-mediated WDR5 degradation and that Asp^172^ of WDR5 had a greater effect than Tyr^191^ of WDR5 on degradation. Collectively, these results indicated that MS67 was a selective WDR5 degrader and encouraged us to explore potential utilities of this WDR5 degrader.

### MS67 suppresses transcription of WDR5-regulated genes

**Fig. 5.**
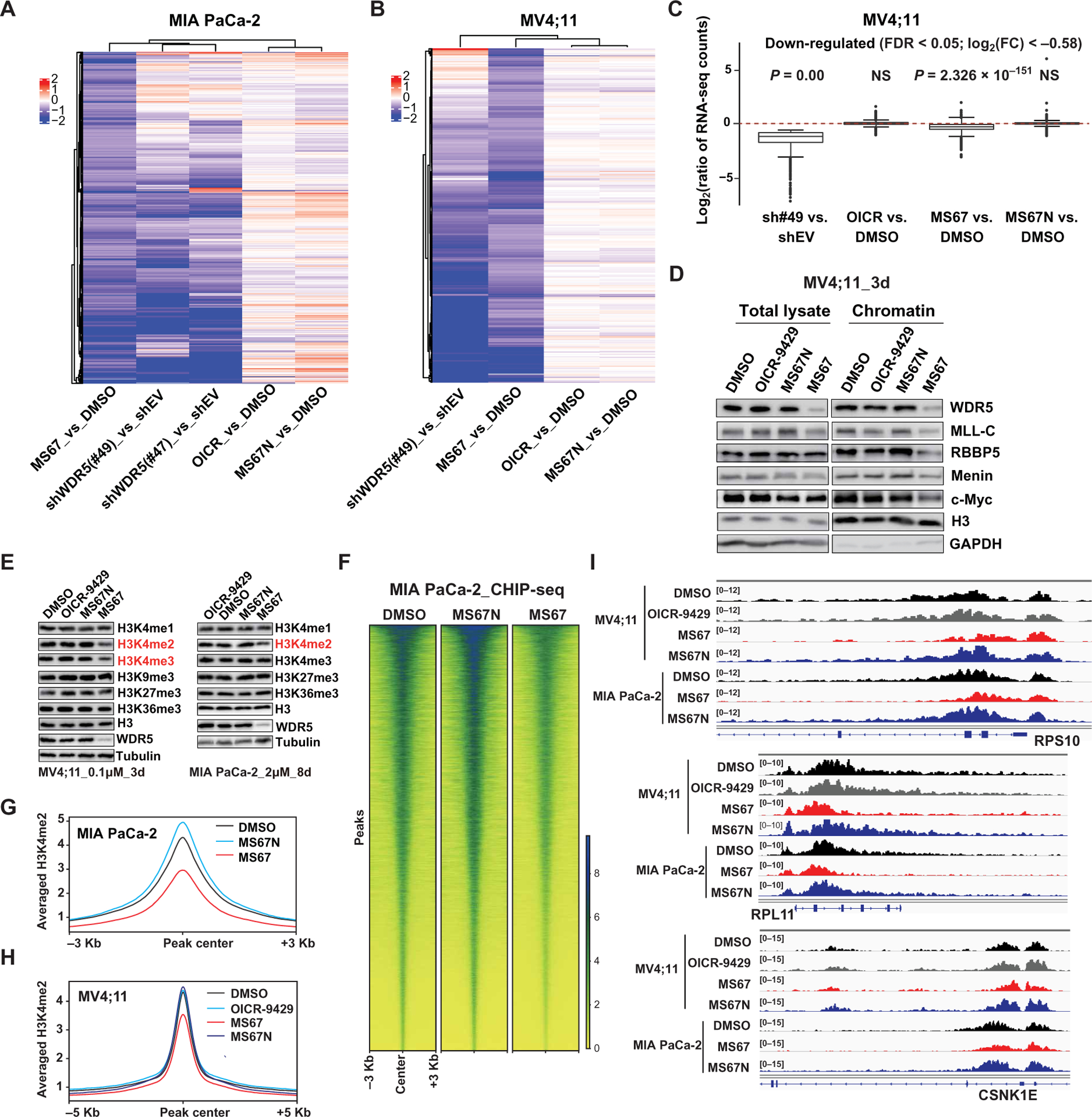
MS67 is effective in suppressing transcription of WDR5-regulated genes and H3K4me2 on chromatin. (**A**) Heatmaps using the indicated sample comparisons show log_2_ ratios for 842 genes significantly down-regulated in MIA PaCa-2 cells after a 6-day treatment with 1 μM MS67, relative to mock. Down-regulation is determined with a cutoff of log_2_[fold change (FC)] less than −0.58 and false discovery rate (FDR) less than 0.05. Comparisons were done using RNA-seq profiles of cells transduced with a WDR5-targeting shRNA (either sh#47 or sh#49) versus empty vector (shEV) (0.5 μg/ml Dox, 4-day treatment) and cells treated with OICR-9429 (1 μM, 6-day treatment) versus DMSO, MS67N (1 μM, 6-day treatment) versus DMSO, or MS67 (1 μM, 6-day treatment) versus DMSO. (**B**) Heatmaps using the indicated sample comparisons show log_2_ ratios for 464 genes significantly down-regulated in MV4;11 cells after a 3-day treatment with 0.1 μM MS67, relative to mock. Down-regulation is determined with a cutoff of log_2_(FC) less than −0.58 and FDR less than 0.05. Comparison was done using RNA-seq profiles of cells transduced with sh#49 versus shEV (0.5 μg/ml Dox, 4-day treatment) and cells treated with OICR-9429 (0.1 μM, 3-day treatment) versus DMSO, MS67N (0.1 μM, 3-day treatment) versus DMSO, or MS67 versus DMSO. (**C**) Box plots showing the log_2_ ratios for genes showing 1529 significant down-regulation in MV4;11 cells transduced with a WDR5-targeting shRNA(sh#49), relative to shEV. Comparison was done across sh#49 versus shEV, OICR-9429 versus DMSO, MS67N versus DMSO, and MS67 versus DMSO. *P* value was generated for each comparison. NS, not significant. (**D**) Immunoblots for the indicated MLL-complex proteins and c-MYC, either in total cell extract or in chromatin-bound fractions, in MV4;11 cells treated with DMSO or 0.1 μM OICR-9429, MS67N, or MS67 for 3 days. GAPDH, glyceraldehyde-3-phosphate dehydrogenase. (**E**) Immunoblots for the indicated histone modifications (with H3 as a loading control) and WDR5 (with Tubulin as control) posttreatment of MV4;11 cells (left; 0.1 μM for 3 days) or MIA PaCa-2 cells (right; 2.0 μM for 8 days) with DMSO, OICR-9429, MS67N, or MS67. (**F**) Heatmap showing the H3K4me2 density of ±3 Kb around the called peaks, as assessed by the spike-in normalized ChIP-seq profiles of MIA PaCa-2 cells treated with DMSO (left), MS67N (2 μM) (middle), or MS67 (2 μM) (right) for 8 days. (**G** and **H**) Averaged H3K4me2 ChIP-seq signals at the called peaks identified under the mock-treated condition posttreatment of MIA PaCa-2 (G) (2 μM for 8 days) and MV4;11 cells (H) (0.1 μM for 3 days) with DMSO, OICR-9429, MS67N, or MS67. (**I**) Integrative Genomics Viewer (IGV) views of the indicated gene locus (RPS10, RPL11, and CSNK1E) showing the H3K4me2 decrease induced by treatment of MV4;11 (0.1 μM for 3 days) and MIA PaCa-2 cells (2 μM for 8 days) with MS67 in comparison with DMSO, OICR-9429, or MS67N treated.

We next evaluated the gene-regulatory effects of MS67 in vitro using RNA sequencing (RNA-seq)–based transcriptome profiling. We first determined WDR5-regulated transcripts using two independent, inducible WDR5-targeting short hairpin RNAs (shRNAs) expressed in MIA PaCa-2 cells (fig. S11, A and B). RNA-seq revealed that differentially expressed genes (DEGs) due to WDR5 knockdown (KD) by the two shRNAs were highly correlated (fig. S11C). RNA-seq profiling of MIA PaCa-2 cells treated with MS67 relative to mock treatment (fig. S11, D and E) identified a substantial portion of DEGs overlapping with those due to WDR5 KD (Fig. 5A and fig. S11F). Most of these WDR5-regulated transcripts did not exhibit significant changes after the treatment with either OICR-9429 or MS67N (Fig. 5A and fig. S11E). We next conducted similar RNA-seq experiments in MV4;11 cells (fig. S11, G to J) and again found that MS67, but not OICR-9429 and MS67N, exhibited suppressing effects on WDR5-mediated gene transcription and that there was a substantial overlap in DEGs between the MS67-treated cells and WDR5 KD cells (Fig. 5, B and C, and fig. S11, I and J). Gene set enrichment analysis revealed that in both MIA PaCa-2 (fig. S12, A to G) and MV4;11 (fig. S13, A to K) cells, treatment by MS67 was positively associated with overall reduced expression of WDR5 direct targets or WDR5-regulated transcripts, reduced activities in protein translation or ribosomes, and down-regulation of transcripts related to c-MYC, hypoxia, and cell proliferation, consistent with what has been reported for WDR5 blockade (17, 28–30, 41). MS67 treatment led to ~15 to 25% overlap of the down-regulated genes in MV4;11 and MIA PaCa-2 cells (fig. S14A). WDR5 KD also resulted in ~15 to 25% overlap of the down-regulated genes in MV4;11 and MIA PaCa-2 cells (fig. S14B). The 94 genes down-regulated upon MS67 treatment in both cell lines also overlapped with those caused by WDR5 KD in the same cell lines (fig. S14, C and D). Gene ontology analysis of the 94 genes down-regulated by the MS67 treatment in both cell lines uncovered enrichment of the gene signatures related to ribosomal components (fig. S14E). These results suggest that different cancer types may share a common response to MS67. Overall, the marked changes in the global transcriptome by MS67, but not OICR-9429 or MS67N, in both MLL-r AML and PDAC cells strongly support that WDR5 degraders such as MS67 are effective in suppressing transcription of WDR5-regulated genes.

In addition to degrading total and chromatin-bound WDR5 (Fig. 5D), MS67 also decreased chromatin-bound fractions of MLL complex components, such as MLL, RBBP5, and Menin, and c-MYC (Fig. 5D), another WDR5 partner in cancer (17, 41). On the other hand, MS67N or OICR-9429 did not decrease the chromatin associations of these WDR5 partners. KD of WDR5 led to global decreases in H3K4me2/3 (fig. S15A), the histone modifications catalyzed by the WDR5-MLL1 complex. We found that MS67, but not MS67N or OICR-9429, phenocopied the effect of WDR5 KD on decreasing H3K4me2/3 in both MV4;11 and MIA PaCa-2 cells (Fig. 5E), whereas other examined histone methylation marks such as H3K9me3, H3K27me3, and H3K36me3 were not affected by any tested compounds (Fig. 5E). We also conducted chromatin immunoprecipitation sequencing (ChIP-seq) of H3K4me2 in MIA PaCa-2 and MV4;11 cells (fig. S15B) and found that MS67 was effective in suppressing gene-associated H3K4me2 (Fig. 5, F to H, and fig. S15C), as exemplified by the reduction of H3K4me2 at cancer-associated genes such as translation-related ribosomal components, BCL2, and HOX cluster genes (Fig. 5I and fig. S15D). Collectively, these results demonstrate that MS67 is effective in suppressing both WDR5-related gene expression programs and WDR5/MLL-induced H3K4 methylations on chromatin.

### MS67 effectively suppresses growth of human cancer cells in vitro and in vivo

**Fig. 6.**
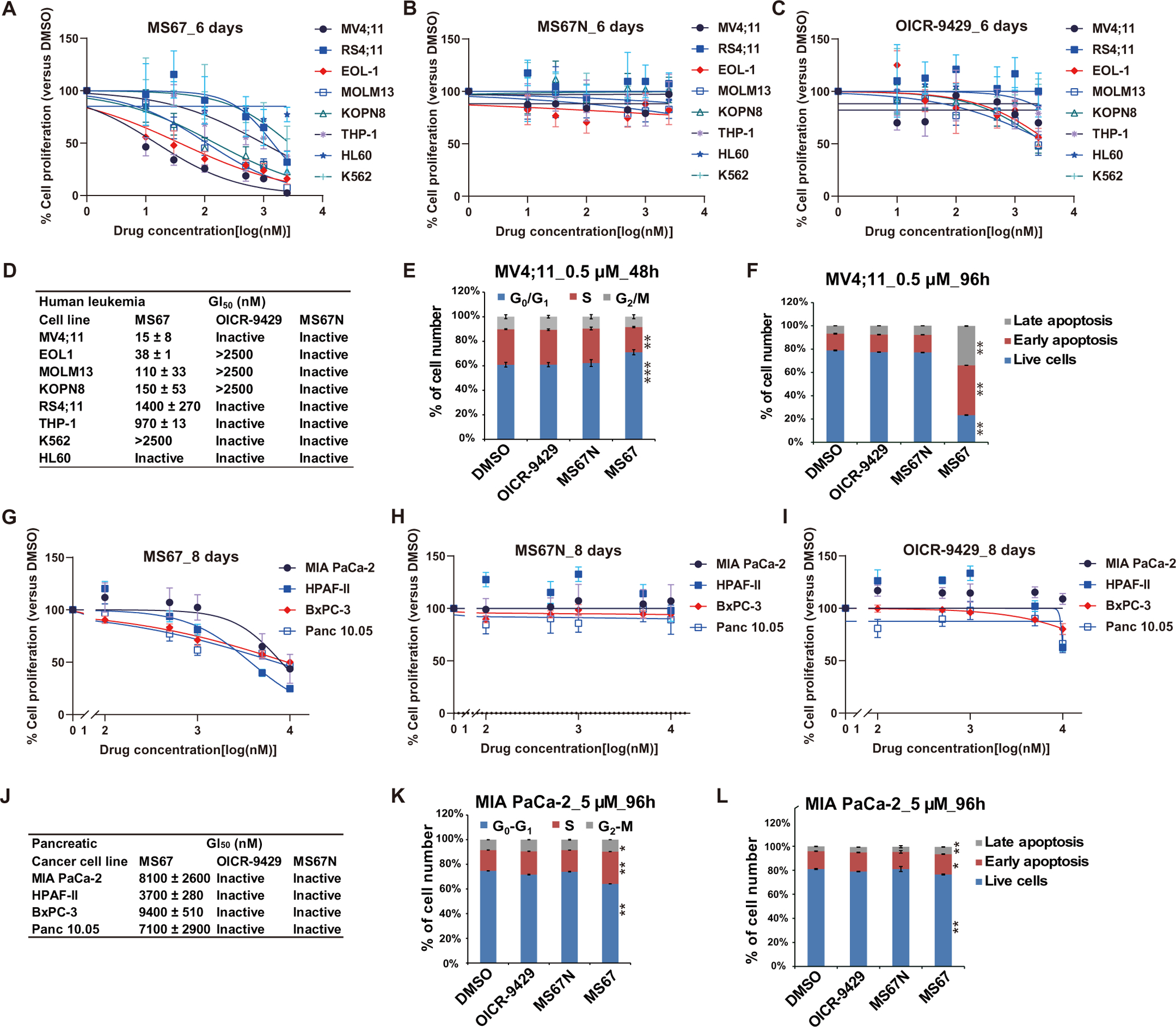
MS67 suppresses the growth of MLL-r AML and PDAC cells in vitro. (**A** to **C**) Growth inhibition curves of MS67 (A), MS67N (B), and OICR-9429 (C) in human leukemia cells, MV4;11, RS4;11; EOL-1, MOLM13, KOPN8, THP-1, HL-60, and K562. *Y* axis, presented in the means ± SEM of data from three independent experiments, shows the relative cell number posttreatment with the indicated concentrations (*x* axis) of compounds for 6 days, normalized to DMSO-treated. (**D**) Summary of GI_50_ values of MS67, MS67N, and OICR-9429 in the human leukemia cells after a 6-day treatment. (**E** and **F**) Cell cycle progression (E) (after a 48-hour treatment) and apoptosis analysis (F) (after a 96-hour treatment) using MV4;11 cells treated with DMSO or 0.5 μM OICR-9429, MS67N, or MS67. Cell cycle index was analyzed by flow cytometry after propidium iodide (PI) staining, with the cell cycle phases indicated at the top of (E). Student’s *t* test, **P < 0.01 and ***P < 0.001. (**G** to **I**) Growth inhibition curves of MS67 (G), MS67N (H), and OICR-9429 (I) in PDAC cells: MIA PaCa-2, HPAF-II, BxPC-3, and Panc 10.05. *Y* axis, presented in the means ± SEM of data from three independent experiments, shows the relative cell number posttreatment with the indicated concentrations (*x* axis) of compounds for 8 days, normalized to DMSO-treated. (**J**) Summary of GI_50_ values of MS67, MS67N, and OICR-9429 in the PDAC cells after an 8-day treatment. (**K** and **L**) Cell cycle progression (K) and apoptosis analysis (L) using MIA PaCa-2 cells treated with DMSO or 5 μM OICR-9429, MS67N, or MS67 for 96 hours. Cell cycle index was analyzed by flow cytometry after PI staining, with the cell cycle phases indicated at the top of (K). Student’s *t* test, *P < 0.1 and **P < 0.01.

Next, we evaluated the antiproliferative effects of MS67 in MLL-r AML and PDAC cells. Compared to OICR-9429, MS67 showed increased inhibition of in vitro growth in a panel of MLL-r AML lines, whereas MS67N was unable to suppress their growth (Fig. 6, A to D, and fig. S16). The effect of MS67 phenocopied that of WDR5 KD in MV4;11 and MOLM13 cells (fig. S17, A and B). Half-maximal growth inhibition concentration (GI_50_) values of MS67 in the two most sensitive AML lines, MV4;11 and EOL-1, were 15 ± 8 nM and 38 ± 1 nM, respectively, whereas the GI_50_ values of OICR-9429 in these two cell lines was greater than 2500 nM (Fig. 6, A, C, and D). MLL-r acute leukemia cell lines including MV4;11, EOL-1, MOLM13, KOPN8, RS4;11, and THP-1 were sensitive to MS67, whereas leukemia cell lines that did not harbor MLL-r (including K562, HL60, and a murine AML line transformed by Hoxa9 plus Meis1) were insensitive to MS67 (Fig. 6, A and D, and fig. S17C). MS67 was also much more effective than OICR-9429 or MS67N in arresting cell cycle progression (Fig. 6E and fig. S18A, top) and inducing apoptosis in sensitive AML cells (Fig. 6F and figs. S18B, top, and S19A). All three compounds had little or no effects on cell cycle progression and apoptosis in the three insensitive leukemia cell lines [figs. S18, A (bottom) and B (bottom), and S19B]. Similarly, MS67, but not OICR-9429 or MS67N, decreased in vitro growth of the four PDAC cell lines tested (Fig. 6, G to J), caused cell cycle progression defects (Fig. 6K), and increased apoptosis (Fig. 6L and fig. S19A) in MIA Paca-2 cells. MS67 was less effective in killing PDAC cells compared to MLL-r AML cells as illustrated by its GI_50_ values (Fig. 6, D versus J). Moreover, the effect of MS67 on cell growth inhibition was similar to that of WDR5 KD in three PDAC cell lines (MIA Paca-2, BxPC-3, and Panc 10.05) (fig. S20, A to C). We found that MS67 exerted minimum growth inhibition effects (GI_50_ > 30 μM) in four additional human cancer cell lines [MCF7 (breast), NCI-H2009 (lung), PC3 (prostate), and SK-ES-1 (bone)] (fig. S20D). These negative results were largely in agreement to the reported effect of WDR5 KD/KO in these cells (22) and data in the Cancer Cell Line Encyclopedia database and suggested that MS67 is not a nonselective cytotoxic agent. Overall, our results indicated that MS67 was better than WDR5 PPI inhibitors in inhibiting cancer cell growth in vitro.

**Fig. 7.**
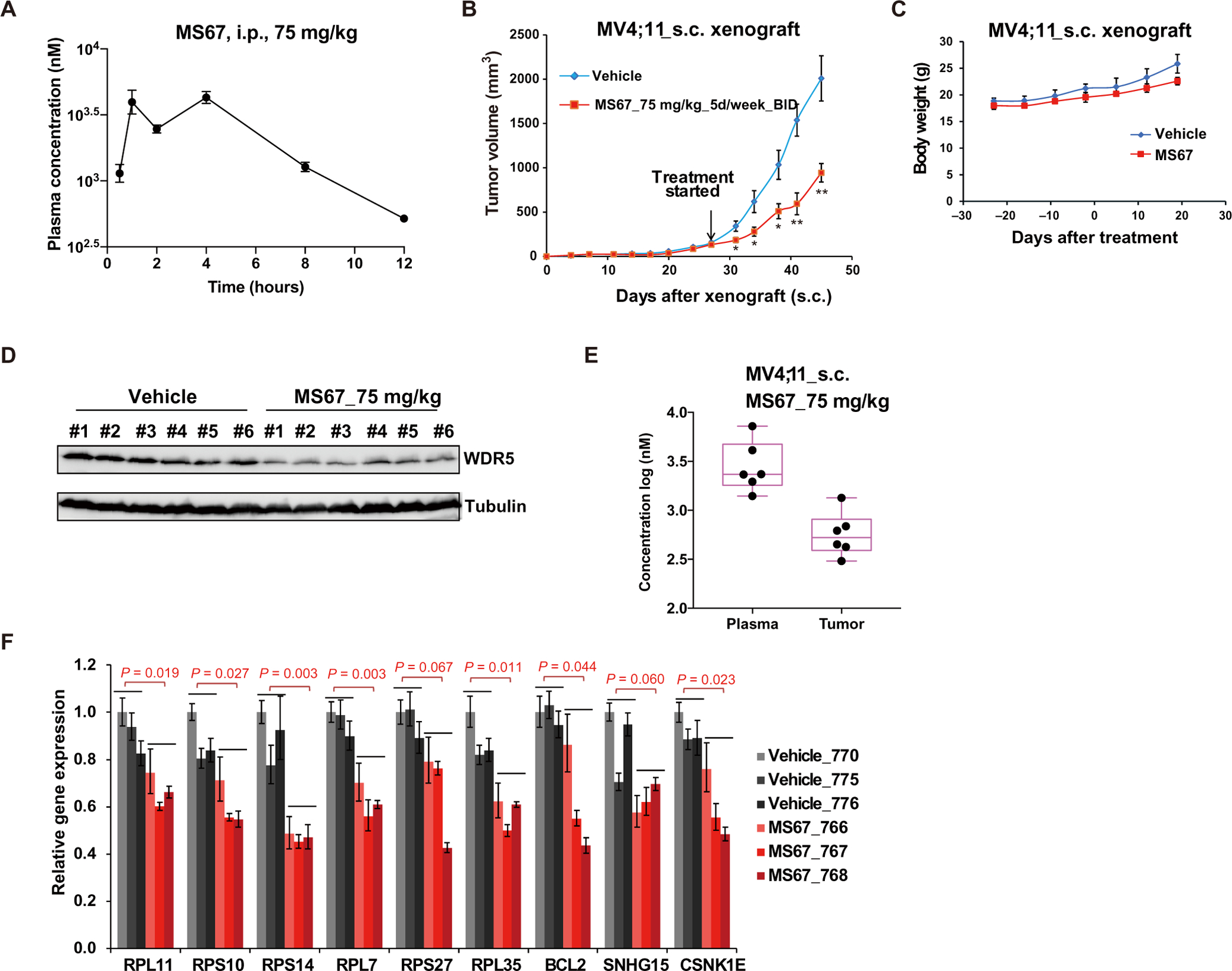
MS67 is efficacious in vivo in a MLL-r AML xenograft model. (**A**) Plasma concentrations of MS67 over a 12-hour period in mice after a single intraperitoneal (i.p.) injection of MS67 (75 mg/kg). The plasma concentrations represent the means ± SEM from three mice per time point. (**B**) The effect of MS67 treatment on the growth of MV4;11 tumors xenografted subcutaneously (s.c.). Tumor-bearing NOD/SCID/gamma(c)(null) (NSG) mice were treated with either vehicle (blue; *n* = 8) or MS67 [75 mg/kg, i.p. twice daily (BID); red; *n* = 10] for 5 days per week, starting at day 26 after inoculation. *Y* axis shows the tumor volumes, measured every 2 to 3 days and presented in the means ± SEM. Student’s *t* test, *P < 0.05 and **P < 0.01. (**C**) Body weights of NSG mice bearing MV4;11 tumor xenografts, treated with either vehicle (blue; *n* = 5) or MS67 (75 mg/kg, i.p. BID; red; *n* = 5) for 5 days per week. (**D**) Immunoblots for WDR5 and Tubulin in tumor samples isolated from NSG mice bearing MV4;11 tumor xenografts. Tumor samples were collected at 2 hours after the last dose from NSG mice treated with vehicle (left) or MS67 (75 mg/kg, i.p. BID; right) for five consecutive days. (**E**) MS67 concentrations in plasma (left) and tumor samples (right) isolated from six NSG mice bearing MV4;11 tumor xenografts. Tumor and plasma samples were collected at 2 hours after the last dose from NSG mice treated with MS67 (75 mg/kg, i.p. BID) for five consecutive days. (**F**) RT-qPCR for the indicated WDR5 target genes in tumor samples isolated from NSG mice bearing MV4;11 tumor xenografts. Tumor samples were collected at 2 hours after the last dose from NSG mice treated with vehicle (gray, *n* = 3) or MS67 (75 mg/kg, i.p. BID; red) for five consecutive days.

We next evaluated in vivo mouse pharmacokinetic (PK) properties of MS67. After a single intraperitoneal (i.p.) injection of MS67 at a dose of 75 mg/kg, the maximum plasma concentration (*C*_max_) reached at about 4.2 μM, and the concentration of MS67 retained above 0.5 μM over 12 hours (Fig. 7A). Because MS67’s GI_50_ values in PDAC cells such as MIA PaCa-2 (8100 ± 2,600 nM) and HPAF-II (3700 ± 280 nM) were much higher than that in MV4;11 cells (15 ± 8 nM), we chose to use the MV4;11 MLL-r AML xenograft mouse model for in vivo efficacy studies. We treated mice bearing subcutaneous xenografts of MV4;11 cells with twice daily (BID) intraperitoneal injections of MS67 at 75 mg/kg 5 days a week, on day 26 after inoculation, and observed significant (*P* = 0.028 at day 38) inhibition of tumor growth in vivo by MS67, compared to vehicle (Fig. 7B). Xenograft mice treated with MS67 for 20 days did not lose any body weight (Fig. 7C). In tumor samples collected from mice 2 hours after the last dose of MS67 or vehicle for 5 days, we found that WDR5 was substantially degraded in the MS67 treated, compared to the vehicle treated (Fig. 7D). We also determined drug concentrations in the same tumor samples and found that an average concentration of 0.6 μM was achieved for MS67 in the tumor samples (Fig. 7E). An average concentration of 5 μM was reached for MS67 treatment in the plasma samples isolated from the same mice 2 hours after the last dose of MS67 (Fig. 7E). In addition, reverse transcription polymerase chain reaction (RT-PCR) analysis of these tumor samples revealed that the MS67 treatment led to down-regulation of WDR5 target genes such as ribosome subunits and oncogenesis-related transcripts including BCL2 and CSNK1E (Fig. 7F). Therefore, a PK/PD (pharmacodynamic) relationship was established for MS67 in this xenograft model.

**Fig. 8.**
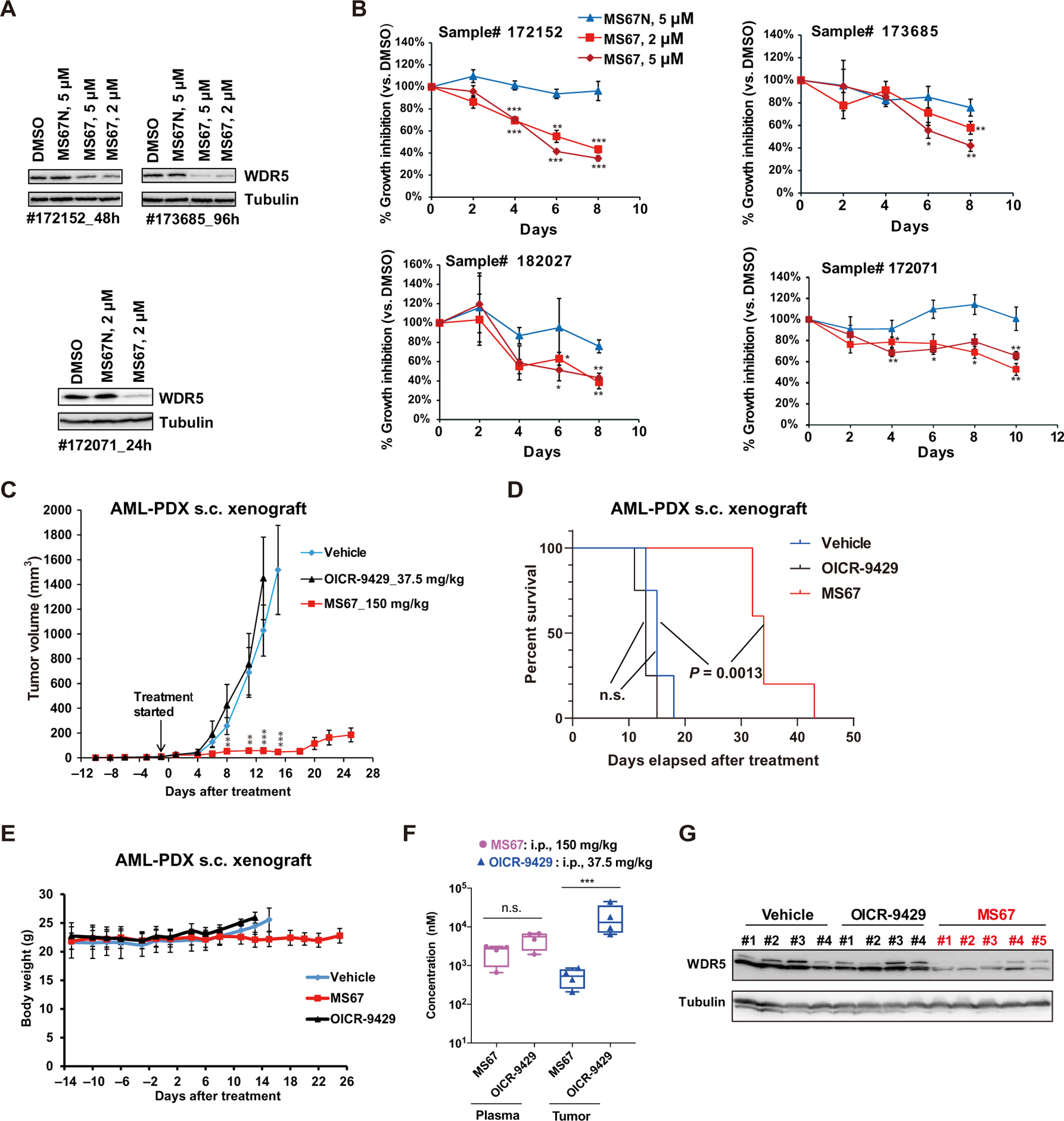
MS67 inhibits the growth of primary AML cells, suppresses tumor growth in vivo, and improves survival in a PDX model. (**A**) Immunoblots for WDR5 and Tubulin in primary AML cells of deidentified patients, which were in vitro cultured and treated with either DMSO or the indicated concentration of MS67 or MS67N for 24 hours (#172071), 48 hours (#172152), or 96 hours (#173685). (**B**) Growth inhibitory activities of MS67 and MS67N treatment in primary AML cells of deidentified patients. *Y* axis, presented in the means ± SEM of data from three independent experiments, shows the relative cell number posttreatment with compounds for the indicated duration (*x* axis), normalized to DMSO-treated. Student’s *t* test, *P < 0.05, **P < 0.01, and ***P < 0.001. (**C** to **E**) Measurement of the growth (C) of MLL-AF9^+^ AML PDX tumors xenografted subcutaneously (s.c.) in NSG-SGM3 mice treated with either vehicle (blue; *n* = 8), MS67 (red; *n* = 10), or OICR-9429 (black; *n* = 8), as well as the Kaplan-Meier survival curve (D) and averaged weight (E) of PDX-xenografted mice treated with either vehicle (blue; *n* = 4), MS67 (red; *n* = 5), or OICR-9429 (black; *n* = 4), starting at day 13 after inoculation. The used intraperitoneal doses of OICR-9492 and MS67 are 37.5 and 150 mg/kg, respectively [BID on Monday, Wednesday, and Friday, and once daily (SID) on Tuesday and Thursday for each week]. Student’s *t* test is used for (C), **P < 0.01 and ***P < 0.001. Log rank test is used for (D). (**F**) MS67 and OICR-9429 concentrations in plasma and tumor samples isolated from the NSG-SGM3 mice treated with MS67 or OICR-9429 in (C). Tumor and plasma samples were collected at 2 hours after the last dose. Unpaired two-sided Student’s *t* test, ***P < 0.001. (**G**) Immunoblots for WDR5 and Tubulin in tumor samples isolated from the NSG-SGM3 mice after the indicated drug treatment in (C). Tumor samples were collected at 2 hours after the last dose.

To further examine the therapeutic potential of MS67, we next assessed its effects on degrading WDR5 and inhibiting cell growth in primary cancer samples isolated from deidentified patients with AML (fig. S21A). We found that MS67, but not MS67N, effectively reduced WDR5 protein expression and suppressed the growth of these primary cancer cells in vitro (Fig. 8, A and B). Last, we assessed in vivo efficacy of MS67 using a patient-derived xenograft (PDX) mouse model of AML. We first treated mice bearing subcutaneous PDX with MS67 (100 mg/kg, i.p. BID 5 days per week, 11 days after inoculation) and found that MS67, relative to vehicle, significantly (*P* = 0.0026 at day 17) inhibited PDX tumor growth in vivo and significantly (*P* = 0.006) prolonged survival of the treated mice (fig. S21, B and C). Furthermore, no obvious changes in body weight of the MS67-treated mice were observed, again suggesting that MS67 was well tolerated (fig. S21D). RT-PCR analysis of the tumor samples isolated from the treated mice showed that MS67, compared to vehicle, down-regulated WDR5 target genes including oncogenesis-related transcripts such as BCL2 and CSNK1E, and ribosome subunits (fig. S21E). We next compared treatment with OICR-9429 with MS67 treatment in this PDX model. The selected doses for OICR-9429 and MS67 in this study (Fig. 8C) were based on the reported PK data of OICR-9429 (24) and the MS67 doses used in the above studies. We found that MS67, but not OICR-9429, significantly (*P* = 9.69 × 10^−05^ at day 15) suppressed tumor growth in vivo and significantly (*P* = 0.0013) prolonged the survival of mice (Fig. 8, C and D). The MS67 or OICR-9429 treatment did not lead to obvious changes in body weight (Fig. 8E). We determined drug concentrations in plasma and tumor samples isolated from treated mice at the termination of the in vivo study. Although OICR-9429 and MS67 achieved similar concentrations in plasma [5 μM (OICR-9429) versus 1.9 μM (MS67)], the concentration of OICR-9429 in tumor samples (20 μM) was much higher than that of MS67 (0.47 μM) (Fig. 8F). Moreover, MS67, but not OICR-9429, effectively degraded WDR5 in these tumor samples (Fig. 8G). Collectively, these results suggest that MS67 has better antitumor activities than WDR5 PPI inhibitors in vitro and in vivo, is well tolerated in mice, and has potential for further therapeutic development.

## DISCUSSION

Numerous studies have identified WDR5 as a promising potential therapeutic target. Efforts on targeting the WIN or WBM binding site of WDR5 have led to discovery of several inhibitors that potently and selectively block PPIs between WDR5 and its binding partners. However, these WDR5 PPI inhibitors, which rely on receptor occupancy pharmacology and target only some but not all WDR5’s oncogenic functions, exert rather modest cancer cell killing effects in general and lack in vivo efficacy (18–31).

In this study, we investigated pharmacological degradation of WDR5 as an alternative therapeutic strategy to pharmacological inhibition of WDR5 for the treatment of WDR5-dependent cancers. Using the PROTAC technology, we first generated a WDR5 degrader, MS33, and solved the high-resolution crystal structure of the WDR5-MS33-VCB ternary complex. We exploited the ternary complex structure and designed a much more effective WDR5 degrader, MS67. MS67 potently degraded WDR5 in a panel of MLL-r AML and PDAC cells with low-nanomolar DC_50_ and high *D*_max_ values in a VHL, proteasome, WDR5, and time-dependent manner and was highly selective for WDR5 in MS-based global proteomics studies. Using genomics analysis, we showed that MS67 was far more effective than the WDR5 PPI inhibitor OICR-9429 in suppressing overall transcription of WDR5-regulated genes crucially involved in oncogenesis. MS67 displayed greater antiproliferative effects than OICR-9429 in a panel of MLL-r AML and PDAC cells. MS67 was able to effectively reduce the growth of primary cancer cells from patients with AML and suppressed tumor growth in vivo in MLL-r AML xenograft and PDX models. We also showed that MS67, but not OICR-9429, prolonged the survival of mice bearing MLL-r AML PDX. However, MS67 was well tolerated in vivo. Overall, MS67 is a promising agent and has potential for further development.

One limitation of our study was the unexplained variation in the sensitivity of MLL-r AML cell lines and patient samples to MS67 treatment. Among the MLL-r AML cell lines tested, we observed variations in sensitivity to MS67 with MV4;11 and EOL-1 cells being the most sensitive and RS4;11 and THP-1 cells being least sensitive to MS67 treatment. Such variations were also observed with primary cancer cells from patients with AML. We were unable to identify specific genetic background or known mutations of the cell lines and patient samples that may explain the observed difference in sensitivity to MS67. This warrants further investigation. Another limitation of our study was the use of subcutaneous xenograft models to assess in vivo efficacy of MS67. Further evaluation of MS67’s in vivo efficacy in orthotopic in vivo models, which are preferential to subcutaneous models, is merited. Last, the PK properties of MS67 have room for improvement. Further optimization of E3 ligase ligands, linkers, and WDR5-binding moieties will likely result in improved WDR5 degraders.

It is worth noting that, to the best of our knowledge, only a very limited number of degrader ternary complex structures have been reported to date (39, 42–46). Structure-based design to generate more effective degraders is even rarer. Our structure-based design that exploits the crucial insights revealed by the crystal structure of the WDR5-MS33-VCB ternary complex resulted in identification of a highly effective WDR5 degrader, demonstrating the power of the structure-based design approach in the degrader discovery field. The promising in vitro and in vivo antitumor activities exhibited by the WDR5 degrader MS67 strongly suggest that pharmacological degradation of WDR5 is an attractive and promising therapeutic strategy for the treatment of WDR5-dependent tumors.

## MATERIALS AND METHODS

### Study design

The primary research goal of this study was to find and characterize highly effective WDR5 degraders. We designed and synthesized heterobifunctional compounds and evaluated their WDR5 degradation effect using Western blot analyses. Using x-ray crystallography and ITC, we characterized WDR5-MS33-VCB and WDR5-MS67-VCB ternary complexes. We further characterized the lead WDR5 degrader MS67 using MS-based proteomics, RNA-seq, ChIP-seq, cell proliferation, cell cycle, apoptosis, RT–quantitative PCR (qPCR), in vivo PK, and in vivo mouse xenograft and PDX studies. Mouse xenograft and PDX studies were designed to assess the effects of MS67 on tumor growth in vivo, survival, and body weight and to establish PK/PD relationships. All animal studies involving mice were performed according to the Institutional Animal Care and Use Committee–approved protocol. To ensure the reproducibility, the mice were randomized. The experiments were not carried out in a blinded manner. Each experiment was performed with multiple mice (*n* = 4 to 10 per group). The statistical tests and the experimental replicates are indicated in each figure legend individually. No data outliers were excluded in this study. All raw data and primer sequences for the main and supplementary figures and tables are summarized in data file S1.

### Statistical analysis

Experimental data are presented as the means ± SD or SEM of three independent experiments unless otherwise noted. Statistical analysis was performed using an unpaired two-sided Student’s *t* test for comparing two sets of data with assumed normal distribution. A log-rank test was used to determine statistical significance for Kaplan-Meier survival curves. For in vivo tumor progression studies, a two-sided Student’s *t* test was performed to determine the statistical differences in size of tumor xenografts. The results for immunoblotting are representative of at least two biologically independent experiments unless otherwise noted. All statistical analyses and visualizations were performed using GraphPad (Prism v8.4.2) or Excel.

## Supplementary Material

supplementary material PDF

Data File

## Data Availability

All data associated with this study are present in the paper or the Supplementary Materials. Atomic coordinates and structure factors have been deposited in the PDB with accession codes 7JTO (WDR5-MS33-VCB complex) and 7JTP (WDR5-MS67-VCB complex). RNA-seq and ChIP-seq data have been deposited in the Gene Expression Omnibus (GEO) database under the accession number GSE150555. All compounds (including MS33, MS33N, MS67 and MS67N) can be obtained through a standard material transfer agreement by contacting J.J. at jian.jin@mssm.edu.
