# Supplementary material for: A selective WDR5 degrader inhibits acute myeloid leukemia in patient-derived mouse models
Source: Sci Transl Med. Author manuscript; Available in PMC 2022 Mar 29. (PMC8500670; doi:10.1126/scitranslmed.abj1578)
Supplement: supplementary material PDF [file NIHMS1744433-supplement-supplementary_material_PDF.pdf]

Science Translational Medicine
AAAS

Supplementary Materials for

A selective WDR5 degrader inhibits acute myeloid leukemia in patient-derived mouse models

Xufen Yu *et al.*

Corresponding author: Jian Jin, jian.jin@mssm.edu; Gang Greg Wang, greg_wang@med.unc.edu

Sci. Transl. Med. **13**, eabj1578 (2021)
DOI: 10.1126/scitranslmed.abj1578

The PDF file includes:

Materials and Methods
Figs. S1 to S21
Tables S1 to S4
References (*47–81*)

Other Supplementary Material for this manuscript includes the following:

Data file S1

Materials and Methods:

Chemistry

Chemistry General Procedures. All commercial chemical reagents and solvents were used for the reactions without further purification. Microwave-heated reactions were conducted with a Discover SP microwave system with an Explorer 12 Hybrid Autosampler by CEM. Flash column chromatography was performed on Teledyne ISCO Combi*Flash* Rf^{+} instrument equipped with a 220/254/280 nm wavelength UV detector and a fraction collector. Reverse phase column chromatography was conducted on HP C18 Redi*Sep* Rf columns to purify some polar compounds. All final compounds were purified with preparative high-performance liquid chromatography (HPLC) on an Agilent Prep 1200 series with the UV detector set to 220/254 nm at a flow rate of 40 mL/min. Samples were injected onto a Phenomenex Luna 750 x 30 mm, 5 μm C18 column, and the gradient was set to 10% of acetonitrile in H_2O containing 0.1% TFA progressing to 100% of acetonitrile. All compounds assessed for biological activity have purity > 95% as determined by an Agilent 1200 series system with DAD detector and a 2.1 mm x 150 mm Zorbax 300SB-C18 5 μm column for chromatography and high-resolution mass spectra (HRMS) that were acquired in positive ion mode using an Agilent G1969A API-TOF with an electrospray ionization (ESI) source. Samples (0.5 μL) were injected onto a C18 column at room temperature, and the flow rate was set to 0.4 mL/min with water containing 0.1% formic acid as solvent A and acetonitrile containing 0.1% formic acid as solvent B. Proton nuclear magnetic resonance (^{1}H NMR) and carbon nuclear magnetic resonance (^{13}C NMR) spectra were acquired on either Bruker DXI 600 MHz (or 800 MHz) or AVANCE NEO 600 MHz Nuclear Magnetic Resonance (NMR) spectrometer. Chemical shifts for all compounds are reported in parts per million (ppm, δ). The format of chemical shift was reported as follows: chemical shift,

multiplicity (s = singlet, d = doublet, t = triplet, q = quartet, m = multiplet), coupling constant (*J* values in Hz), and integration.

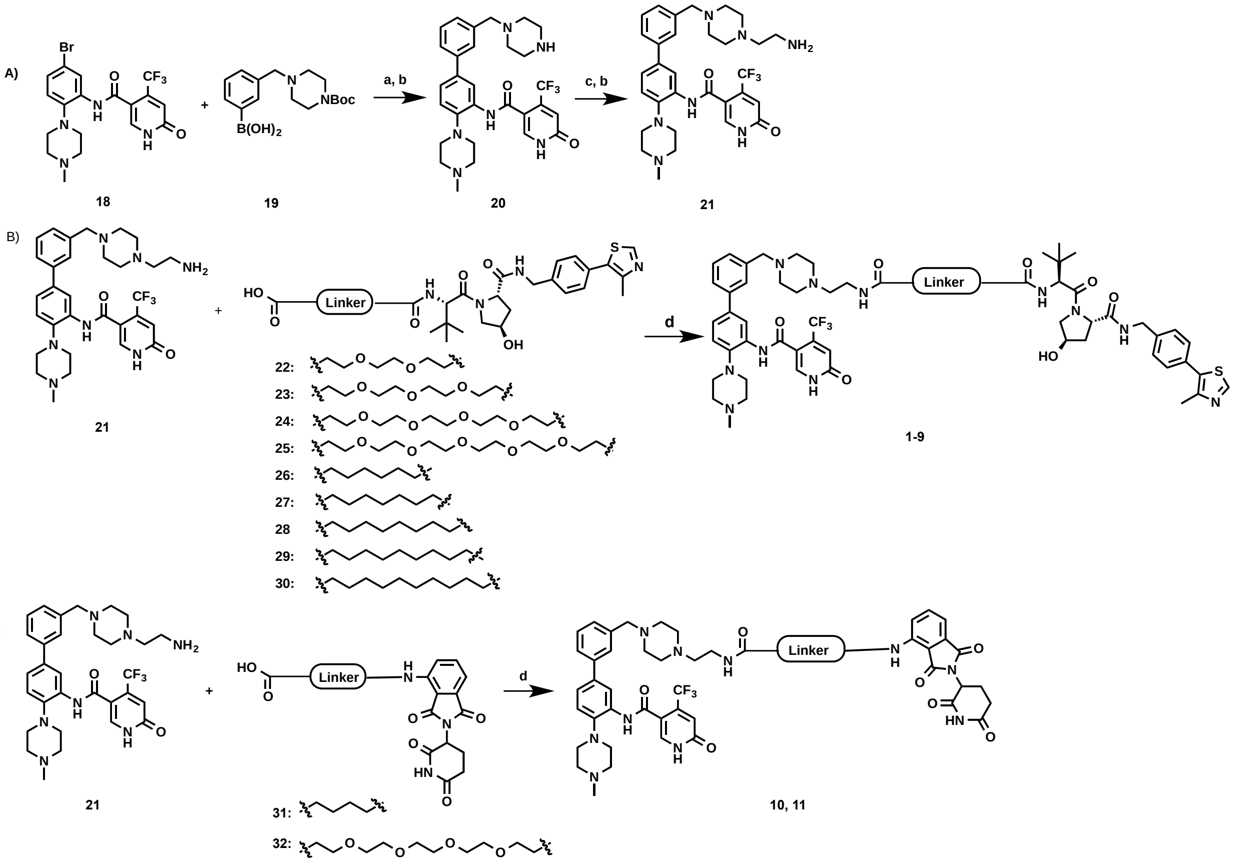

Scheme S1. Reagents and Conditions: (a) XPhos Pd G2, XPhos, Na_2CO_3, dioxane/H_2O (5 : 3), microwave (MW), 120 °C, 1 h; (b) TFA/DCM, rt, 1 h; (c) *tert*-butyl (2-oxoethyl)carbamate, $NaBH(OAc)_3$, DCM, rt, 12 h; (d) EDCI, HOAt, NMM, DMSO, rt, 12 h.

VHL-based linkers 22-30 and pomalidomide-based linkers 31-32 were prepared following the reported procedures.(*47*)

N-(4-(4-Methylpiperazin-1-yl)-3'-(piperazin-1-ylmethyl)-[1,1'-biphenyl]-3-yl)-6-oxo-4-(trifluoromethyl)-1,6-dihydropyridine-3-carboxamide (20)

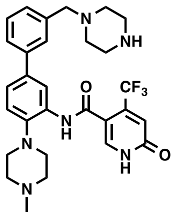

To a solution of *N*-(5-bromo-2-(4-methylpiperazin-1-yl)phenyl)-6-oxo-4-(trifluoromethyl)-1,6-dihydropyridine-3-carboxamide (**18**, 348.6 mg, 0.76 mmol, prepared according to the published procedures (*24*)) and commercially available (3-((4-(*tert*-butoxycarbonyl)piperazin-1-yl)methyl)phenyl)boronic acid (**19**, 729 mg, 2.27 mmol, 3.0 equiv) in 8 mL of 1,4-dioxane / H_2O (5 : 3) were added sodium carbonate (805.6 mg, 7.6 mmol, 10 equiv), XPhos (85 mg, 0.15 mmol, 0.2 equiv), and XPhos Pd G2 (141 mg, 0.15 mmol, 0.2 equiv). The reaction was heated to 120 °C for 1 h under microwave. The solvent was concentrated and purified by reverse phase ISCO (10% – 100% methanol / 0.1% TFA in H_2O) to afford product as white solid in TFA salt form (350 mg, 70% yield). This product was dissolved in DCM (5 mL) and TFA (5 mL). After stirring for 1 h, it was concentrated and purified by reverse phase ISCO (10% – 100% methanol / 0.1% TFA in H_2O) to afford compound **20** as white solid in TFA salt form (290 mg, yield 98%). ^{1}H NMR (600 MHz, DMSO-d_6) δ 9.61 (s, 1H), 8.18 (d, J = 2.2 Hz, 1H), 8.04 (s, 1H), 7.74 – 7.37 (m, 6H), 7.32 (d, J = 8.3 Hz, 1H), 6.85 (d, J = 2.6 Hz, 1H), 3.97 (s, 6H), 3.53 (d, J = 11.4 Hz, 2H), 3.39 – 2.96 (m, 10H), 2.88 (s, 3H). t_R = 3.58 min, HRMS (m/z) for $C_{29}H_{34}F_3N_6O_2^+$ [M + H]$^+$: calculated 555.2690, found 555.2674.

N-(3'-((4-(2-Aminoethyl)piperazin-1-yl)methyl)-4-(4-methylpiperazin-1-yl)-[1,1'-biphenyl]-3-yl)-6-oxo-4-(trifluoromethyl)-1,6-dihydropyridine-3-carboxamide (21)

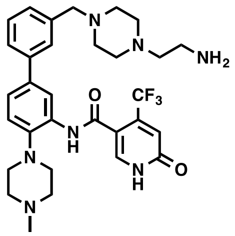

To a solution of compound **20** (238 mg, 0.43 mmol), and *tert*-butyl (2-oxoethyl)carbamate (137 mg, 0.86 mmol, 2.0 equiv) in dichloromethane (10 mL) was added sodium triacetoxyborohydride (183 mg, 0.86 mmol). After stirring overnight, saturated sodium bicarbonate was added. The mixture was extracted with DCM (3 x 10 mL), dried over Na_2SO_4, filtered and concentrated. The mixture was purified by reverse phase ISCO (10% – 100% methanol / 0.1% TFA in H_2O) to afford white solid in TFA salt form (251 mg, yield 84%). This resulting product was dissolved in DCM (5 mL) and TFA (5 mL) and stirring for 1 h. Then, it was concentrated and purified by preparative HPLC (10%-100% methanol / 0.1% TFA in H_2O) to afford compound **21** as white solid in TFA salt form (214 mg, yield 99%). ^1H NMR (600 MHz, CD_3OD) δ 8.29 (d, *J* = 2.2 Hz, 1H), 8.07 (s, 1H), 7.83 (s, 1H), 7.81 – 7.77 (m, 1H), 7.64 – 7.55 (m, 2H), 7.55 – 7.49 (m, 1H), 7.41 (d, *J* = 8.4 Hz, 1H), 6.94 (s, 1H), 4.45 (s, 2H), 3.63 (d, *J* = 11.9 Hz, 2H), 3.50 – 3.26 (m, 6H), 3.26 – 3.15 (m, 2H), 3.10 (dd, *J* = 6.8, 4.8 Hz, 2H), 2.98 (s, 3H), 2.92 – 2.78 (m, 6H), 2.79 – 2.71 (m, 2H). t_R = 3.64 min, HRMS (m/z) for $C_{31}H_{39}F_3N_7O_2^+$ $[M + H]^+$: calculated 598.3112, found 598.3083.

N-(3'-((4-((S)-15-((2S,4R)-4-hydroxy-2-((4-(4-methylthiazol-5-yl)benzyl)carbamoyl)pyrrolidine-1-carbonyl)-16,16-dimethyl-4,13-dioxo-7,10-dioxa-3,14-diazaheptadecyl)piperazin-1-yl)methyl)-4-(4-methylpiperazin-1-yl)-[1,1'-biphenyl]-3-yl)-6-oxo-4-(trifluoromethyl)-1,6-dihydropyridine-3-carboxamide (1).

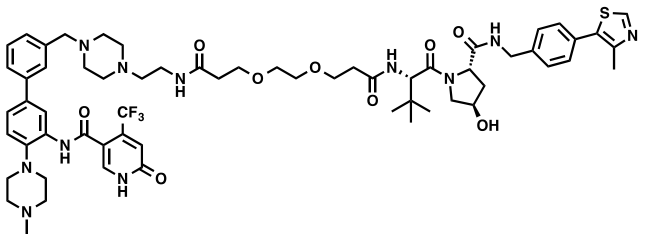

To a solution of compound **21** (13 mg, 0.018 mmol) in DMSO (1 mL) were added linker **22** (11.1 mg, 0.018 mmol, 1 equiv), 1-ethyl-3-(3-dimethylaminopropyl)carbodiimide (EDCI, 5.3 mg, 0.027 mmol, 1.5 equiv), 1-hydroxy-7-azabenzo-triazole (HOAt, 3.7 mg, 0.027 mmol, 1.5 equiv), and N-methylmorpholine (NMM, 5.6 mg, 0.054 mmol, 3 equiv). After stirring overnight at room temperature, the resulting mixture was purified by preparative HPLC (10%-100% methanol / 0.1% TFA in H_2O) to afford compound **1** as white solid in TFA salt form (15.4 mg, yield 71%). ^{1}H NMR (600 MHz, CD_3OD) δ 8.97 (s, 1H), 8.26 (d, *J* = 2.2 Hz, 1H), 8.03 (s, 1H), 7.74 (d, *J* = 1.8 Hz, 1H), 7.71 – 7.67 (m, 1H), 7.55 – 7.50 (m, 2H), 7.48 – 7.42 (m, 3H), 7.42 – 7.39 (m, 2H), 7.38 (d, *J* = 8.4 Hz, 1H), 6.94 (s, 1H), 4.63 (s, 1H), 4.58 – 4.44 (m, 3H), 4.36 (d, *J* = 15.5 Hz, 1H), 4.18 (s, 2H), 3.88 (d, *J* = 10.9 Hz, 1H), 3.78 (dd, *J* = 11.0, 3.9 Hz, 1H), 3.74 – 3.64 (m, 5H), 3.64 – 3.53 (m, 7H), 3.48 (t, *J* = 6.0 Hz, 2H), 3.30 – 3.26 (m, 5H), 3.26 – 3.10 (m, 7H), 3.06 (t, *J* = 6.0 Hz, 2H), 2.96 (s, 3H), 2.58 – 2.51(m, 1H), 2.50 – 2.41 (m, 6H), 2.26 – 2.16 (m, 1H), 2.10 – 2.03 (m, 1H), 1.02 (s, 9H). t_R = 3.60 min, HRMS (m/z) for $C_{61}H_{79}F_3N_{11}O_9S^+$ $[M + H]^+$: calculated 1198.5730, found 1198.5751.

N-(3'-((4-((S)-18-((2S,4R)-4-hydroxy-2-((4-(4-methylthiazol-5-yl)benzyl)carbamoyl)pyrrolidine-1-carbonyl)-19,19-dimethyl-4,16-dioxo-7,10,13-trioxa-3,17-diazaicosyl)piperazin-1-yl)methyl)-4-(4-methylpiperazin-1-yl)-[1,1'-biphenyl]-3-yl)-6-oxo-4-(trifluoromethyl)-1,6-dihydropyridine-3-carboxamide (2).

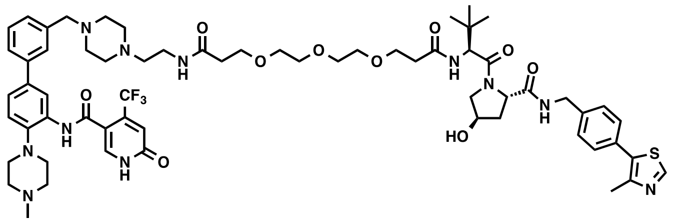

Compound **2** was synthesized following the standard procedures for preparing compound **1** from intermediate **21** (13.0 mg, 0.018 mmol), linker **23** (12.0 mg, 0.018 mmol, 1.0 equiv). Compound **2** was obtained as white solid in TFA salt form (14.4 mg, yield 64%). ^{1}H NMR (600 MHz, CD_3OD) δ 9.01 (s, 1H), 8.26 (d, J = 2.4 Hz, 1H), 8.03 (s, 1H), 7.82 – 7.62 (m, 2H), 7.59 – 7.21 (m, 8H), 6.94 (s, 1H), 4.62 (s, 1H), 4.58 – 4.42 (m, 3H), 4.36 (d, J = 15.5 Hz, 1H), 4.25 (s, 2H), 3.87 (d, J = 11.0 Hz, 1H), 3.78 (dd, J = 10.9, 4.0 Hz, 1H), 3.74 – 3.64 (m, 4H), 3.64 – 3.45 (m, 13H), 3.39 (s, 4H), 3.29 – 3.03 (m, 11H), 2.99 – 2.84 (m, 3H), 2.61 – 2.50 (m, 1H), 2.50 – 2.30 (m, 6H), 2.24 – 2.14 (m, 1H), 2.12 – 2.02 (m, 1H), 1.06 – 0.92 (m, 9H). t_R = 3.58 min, HRMS (m/z) for $C_{63}H_{83}F_3N_{11}O_{10}S^+$ $[M + H]^+$: calculated 1242.5992, found 1242.5973.

N^1-((S)-1-((2S,4R)-4-hydroxy-2-((4-(4-methylthiazol-5-yl)benzyl)carbamoyl)pyrrolidin-1-yl)-3,3-dimethyl-1-oxobutan-2-yl)-N^{16}-(2-(4-((4'-(4-methylpiperazin-1-yl)-3'-(6-oxo-4-(trifluoromethyl)-1,6-dihydropyridine-3-carboxamido)-[1,1'-biphenyl]-3-yl)methyl)piperazin-1-yl)ethyl)-4,7,10,13-tetraoxahexadecanediamide (3).

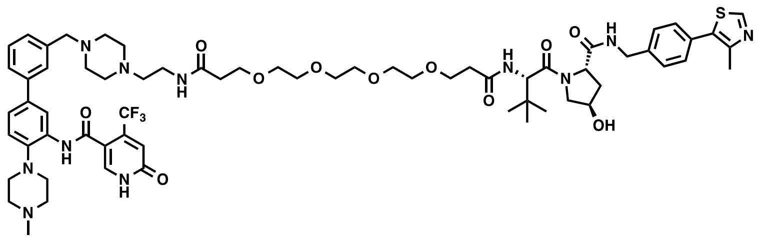

Compound **3** was synthesized following the standard procedures for preparing compound **1** from intermediate **21** (13.0 mg, 0.018 mmol), linker **24** (12.7 mg, 0.018 mmol, 1.0 equiv). Compound **3** was obtained as white solid in TFA salt form (15.4 mg, yield 67%). ^{1}H NMR (600 MHz, CD_3OD) δ 9.04 (s, 1H), 8.26 (t, *J* = 2.2 Hz, 1H), 8.03 (d, *J* = 2.1 Hz, 1H), 7.78 (d, *J* = 2.3 Hz, 1H), 7.72 (d, *J* = 7.8 Hz, 1H), 7.58 – 7.51 (m, 2H), 7.47 (dd, *J* = 8.3, 2.1 Hz, 3H), 7.42 (dd, *J* = 8.3, 2.2 Hz, 2H), 7.38 (dd, *J* = 8.4, 2.2 Hz, 1H), 6.93 (d, *J* = 2.1 Hz, 1H), 4.62 (d, *J* = 2.2 Hz, 1H), 4.58 – 4.46 (m, 3H), 4.36 (dd, *J* = 15.6, 2.2 Hz, 1H), 4.30 (d, *J* = 2.1 Hz, 2H), 3.87 (d, *J* = 11.0 Hz, 1H), 3.81 – 3.75 (m, 1H), 3.75 – 3.65 (m, 5H), 3.64 – 3.51 (m, 19H), 3.51 – 3.34 (m, 8H), 3.22 – 3.15 (m, 4H), 2.96 (d, *J* = 2.0 Hz, 3H), 2.60 – 2.51 (m, 1H), 2.51 – 2.41 (m, 6H), 2.22 (dd, *J* = 13.4, 7.9 Hz, 1H), 2.11 – 2.03 (m, 1H), 1.03 (d, *J* = 2.2 Hz, 9H). t_R = 3.59 min, HRMS (m/z) for $C_{65}H_{87}F_3N_{11}O_{11}S^+$ $[M + H]^+$: calculated 1286.6254, found 1286.6247.

N^1-((S)-1-((2S,4R)-4-hydroxy-2-((4-(4-methylthiazol-5-yl)benzyl)carbamoyl)pyrrolidin-1-yl)-3,3-dimethyl-1-oxobutan-2-yl)-N^{19}-(2-(4-((4'-(4-methylpiperazin-1-yl)-3'-(6-oxo-4-(trifluoromethyl)-1,6-dihydropyridine-3-carboxamido)-[1,1'-biphenyl]-3-yl)methyl)piperazin-1-yl)ethyl)-4,7,10,13,16-pentaoxanonadecanediamide (4).

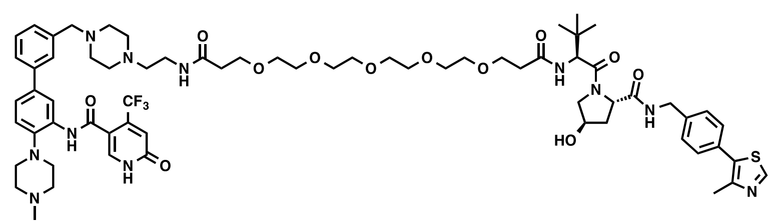

Compound **4** was synthesized following the standard procedures for preparing compound **1** from intermediate **21** (13.0 mg, 0.018 mmol), linker **25** (13.5 mg, 0.018 mmol, 1.0 equiv). Compound **4** was obtained as white solid in TFA salt form (12.1 mg, yield 51%). ^{1}H NMR (600 MHz, CD_3OD) δ 8.93 (s, 1H), 8.26 (d, *J* = 2.2 Hz, 1H), 8.03 (s, 1H), 7.73 (d, *J* = 2.1 Hz, 1H), 7.68 (d, *J*

= 7.8 Hz, 1H), 7.56 – 7.48 (m, 2H), 7.48 – 7.32 (m, 6H), 6.94 (s, 1H), 4.63 (d, *J* = 1.7 Hz, 1H), 4.58 – 4.46 (m, 3H), 4.35 (d, *J* = 15.4 Hz, 1H), 4.14 (s, 2H), 3.87 (d, *J* = 11.0 Hz, 1H), 3.79 (dd, *J* = 11.0, 3.8 Hz, 1H), 3.75 – 3.65 (m, 5H), 3.64 – 3.53 (m, 18H), 3.49 (t, *J* = 6.0 Hz, 2H), 3.28 (s, 6H), 3.16 (t, *J* = 12.3 Hz, 7H), 3.05 (t, *J* = 5.8 Hz, 2H), 2.96 (d, *J* = 1.9 Hz, 3H), 2.61 – 2.52 (m, 1H), 2.49 – 2.41 (m, 6H), 2.25 – 2.17 (m, 1H), 2.11 – 203 (m, 1H), 1.02 (d, *J* = 1.8 Hz, 9H). t_R = 3.59 min, HRMS (m/z) for $C_{67}H_{91}F_3N_{11}O_{12}S^+$ $[M + H]^+$: calculated 1330.6516, found 1330.6528.

N^1-((S)-1-((2S,4R)-4-hydroxy-2-((4-(4-methylthiazol-5-yl)benzyl)carbamoyl)pyrrolidin-1-yl)-3,3-dimethyl-1-oxobutan-2-yl)-N^8-(2-(4-((4'-(4-methylpiperazin-1-yl)-3'-(6-oxo-4-(trifluoromethyl)-1,6-dihydropyridine-3-carboxamido)-[1,1'-biphenyl]-3-yl)methyl)piperazin-1-yl)ethyl)octanediamide (5).

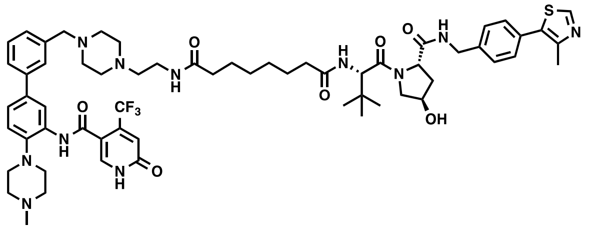

Compound **5** was synthesized following the standard procedures for preparing compound **1** from intermediate **21** (13.0 mg, 0.018 mmol), linker **26** (10.5 mg, 0.018 mmol, 1.0 equiv). Compound **5** was obtained as white solid in TFA salt form (11.5 mg, yield 55%). ^{1}H NMR (600 MHz, CD_3OD) δ 8.92 (s, 1H), 8.26 (d, *J* = 2.2 Hz, 1H), 8.03 (s, 1H), 7.73 (d, *J* = 2.0 Hz, 1H), 7.68 (d, *J* = 7.9 Hz, 1H), 7.56 – 7.48 (m, 2H), 7.48 – 7.33 (m, 6H), 6.94 (s, 1H), 4.63 (s, 1H), 4.60 – 4.44 (m, 3H), 4.36 (d, *J* = 15.5 Hz, 1H), 4.13 (s, 2H), 3.89 (d, *J* = 10.9 Hz, 1H), 3.79 (dd, *J* = 10.9, 4.0 Hz, 1H), 3.61 (d, *J* = 11.7 Hz, 2H), 3.43 (t, *J* = 6.1 Hz, 2H), 3.35 – 3.31 (m, 1H), 3.28 (s, 3H), 3.24 – 3.04 (m, 10H), 2.98 – 2.92 (m, 5H), 2.48 – 2.43 (m, 3H), 2.33 – 2.16 (m, 5H), 2.11 – 2.03

(m, 1H), 1.59 (s, 4H), 1.36 – 1.29 (m, 4H), 1.02 (s, 9H). t_R = 3.64 min, HRMS (m/z) for $C_{61}H_{79}F_3N_{11}O_7S^+$ [M + H]$^+$: calculated 1166.5831, found 1166.5804.

N[1]-((S)-1-((2S,4R)-4-hydroxy-2-((4-(4-methylthiazol-5-yl)benzyl)carbamoyl)pyrrolidin-1-yl)-3,3-dimethyl-1-oxobutan-2-yl)-N[9]-(2-(4-((4'-(4-methylpiperazin-1-yl)-3'-(6-oxo-4-(trifluoromethyl)-1,6-dihydropyridine-3-carboxamido)-[1,1'-biphenyl]-3-yl)methyl)piperazin-1-yl)ethyl)nonanediamide (6).

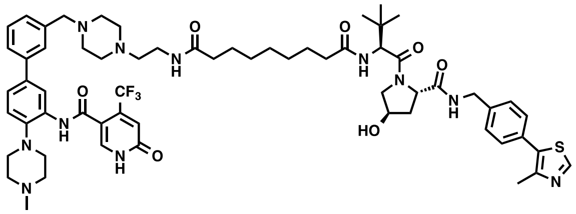

Compound **6** was synthesized following the standard procedures for preparing compound **1** from intermediate **21** (13.0 mg, 0.018 mmol), linker **27** (10.5 mg, 0.018 mmol, 1.0 equiv). Compound **6** was obtained as white solid in TFA salt form (10.4 mg, yield 49%). ^{1}H NMR (600 MHz, CD_3OD) δ 8.92 (s, 1H), 8.26 (d, *J* = 2.6 Hz, 1H), 8.03 (s, 1H), 7.76 – 7.70 (m, 1H), 7.68 (d, *J* = 7.9 Hz, 1H), 7.56 – 7.49 (m, 2H), 7.49 – 7.34 (m, 6H), 6.94 (s, 1H), 4.62 (s, 1H), 4.60 – 4.46 (m, 3H), 4.36 (d, *J* = 15.8 Hz, 1H), 4.13 (s, 2H), 3.89 (d, *J* = 10.7 Hz, 1H), 3.79 (dd, *J* = 11.0, 4.0 Hz, 1H), 3.61 (d, *J* = 11.5 Hz, 2H), 3.43 (t, *J* = 6.2 Hz, 2H), 3.37 – 3.33 (m, 1H), 3.28 (s, 3H), 3.24 – 3.05 (m, 10H), 2.99 – 2.93 (m, 5H), 2.50 – 2.45 (m, 3H), 2.32 – 2.17 (m, 5H), 2.12 – 2.04 (m, 1H), 1.66 – 1.54 (m, 4H), 1.35 – 1.29 (m, 6H), 1.03 (s, 9H). t_R = 3.60 min, HRMS (m/z) for $C_{62}H_{81}F_3N_{11}O_7S^+$ [M + H]$^+$: calculated 1180.5988, found 1180.5975.

N[1]-((S)-1-((2S,4R)-4-hydroxy-2-((4-(4-methylthiazol-5-yl)benzyl)carbamoyl)pyrrolidin-1-yl)-3,3-dimethyl-1-oxobutan-2-yl)-N[10]-(2-(4-((4'-(4-methylpiperazin-1-yl)-3'-(6-oxo-4-

(trifluoromethyl)-1,6-dihydropyridine-3-carboxamido)-[1,1'-biphenyl]-3-yl)methyl)piperazin-1-yl)ethyl)decanediamide (7)

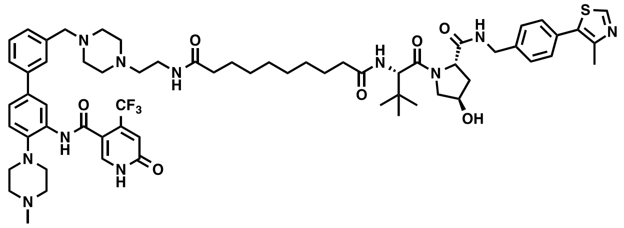

Compound **7** was synthesized following the standard procedures for preparing compound **1** from intermediate **21** (13.0 mg, 0.018 mmol), linker **28** (11.1 mg, 0.018 mmol, 1.0 equiv). Compound **7** was obtained as white solid in TFA salt form (7.2 mg, yield 34%). ^{1}H NMR (600 MHz, CD_3OD) δ 8.91 (d, *J* = 1.9 Hz, 1H), 8.28 – 8.24 (m, 1H), 8.03 (d, *J* = 1.8 Hz, 1H), 7.75 – 7.70 (m, 1H), 7.68 (d, *J* = 7.7 Hz, 1H), 7.57 – 7.48 (m, 2H), 7.48 – 7.34 (m, 6H), 6.94 (d, *J* = 3.9 Hz, 1H), 4.63 (d, *J* = 1.8 Hz, 1H), 4.59 – 4.46 (m, 3H), 4.35 (d, *J* = 15.8 Hz, 1H), 4.13 (s, 2H), 3.92 – 3.86 (m, 1H), 3.82 – 3.77 (m, 1H), 3.61 (d, *J* = 11.6 Hz, 2H), 3.46 – 3.41 (m, 2H), 3.34 (t, *J* = 1.6 Hz, 1H), 3.28 (s, 3H), 3.25 – 3.03 (m, 10H), 2.98 – 2.92 (m, 5H), 2.47 (dd, *J* = 3.8, 1.6 Hz, 3H), 2.32 – 2.16 (m, 5H), 2.12 – 2.04 (m, 1H), 1.58 (s, 4H), 1.31 (s, 8H), 1.03 (s, 9H). t_R = 3.75 min, HRMS (m/z) for $C_{63}H_{83}F_3N_{11}O_7S^+$ $[M + H]^+$: calculated 1194.6144, found 1194.6126.

N^1-((S)-1-((2S,4R)-4-Hydroxy-2-((4-(4-methylthiazol-5-yl)benzyl)carbamoyl)pyrrolidin-1-yl)-3,3-dimethyl-1-oxobutan-2-yl)-N^{11}-(2-(4-((4'-(4-methylpiperazin-1-yl)-3'-(6-oxo-4-(trifluoromethyl)-1,6-dihydropyridine-3-carboxamido)-[1,1'-biphenyl]-3-yl)methyl)piperazin-1-yl)ethyl)undecanediamide (8, MS33)

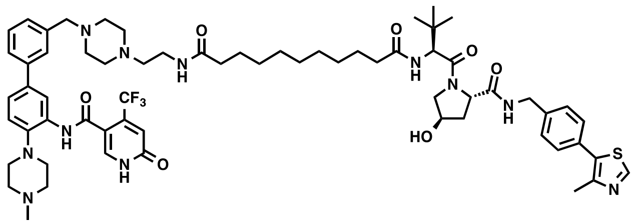

Compound **8** was synthesized following the standard procedures for preparing compound **1** from intermediate **21** (13.0 mg, 0.018 mmol), linker **29** (11.3 mg, 0.018 mmol, 1.0 equiv). Compound **8** was obtained as white solid in TFA salt form (7.7 mg, yield 35%). ^{1}H NMR (800 MHz, CD_3OD) δ 8.99 (s, 1H), 8.29 (s, 1H), 8.06 (s, 1H), 7.78 (s, 1H), 7.74 (s, 1H), 7.59 – 7.54 (m, 2H), 7.49 (t, J = 8.9 Hz, 3H), 7.44 (d, J = 7.7 Hz, 1H), 7.41 (d, J = 8.2 Hz, 2H), 6.96 (s, 1H), 4.66 (s, 1H), 4.59 (t, J = 8.3 Hz, 1H), 4.56 (d, J = 15.4 Hz, 1H), 4.52 (s, 1H), 4.39 (d, J = 15.2 Hz, 1H), 4.25 (s, 2H), 3.93 (d, J = 10.9 Hz, 1H), 3.83 (dd, J = 10.9, 3.9 Hz, 1H), 3.64 (d, J = 11.7 Hz, 2H), 3.50 (t, J = 6.2 Hz, 2H), 3.33 (dd, J = 22.9, 15.6 Hz, 12H), 3.20 (t, J = 12.5 Hz, 2H), 3.08 (t, J = 6.1 Hz, 2H), 2.99 (s, 3H), 2.50 (s, 3H), 2.36 – 2.29 (m, 1H), 2.29 – 2.19 (m, 4H), 2.14 – 2.06 (m, 1H), 1.67 – 1.54 (m, 4H), 1.33 (s, 10H), 1.06 (s, 9H). ^{13}C NMR (201 MHz, CD_3OD) δ 176.00, 174.66, 173.09, 170.97, 163.92, 162.53, 151.76, 147.12, 142.30, 140.96, 140.07 (q, $^{2}J_{C\text{-}F}$ = 34.2 Hz, 1C, -*C-CF_3), 139.07, 138.34, 137.57, 132.63, 132.37, 131.96, 129.83, 129.37, 128.97 (2C), 128.73, 127.60 (2C), 124.45, 122.13, 122.04 (q, $^{1}J_{C\text{-}F}$ = 275.4 Hz, 1C, -$^{*}CF_3$), 120.98, 119.23, 117.12, 115.68, 113.05, 69.68, 60.33, 59.46, 57.58, 56.62, 56.24, 53.60 (2C), 50.11, 49.68, 48.93, 42.29 (2C), 37.55, 35.50, 35.26, 35.19, 34.61, 29.01, 28.96 (2C), 28.93 (2C), 28.87 (2C), 25.64 (3C), 25.60 (2C), 25.29, 14.21. t_R = 4.06 min, HRMS (m/z) for $C_{64}H_{85}F_3N_{11}O_7S^+$ [M + H]$^+$: calculated 1208.6301, found 1208.6313.

N^{1}-((S)-1-((2S,4R)-4-hydroxy-2-((4-(4-methylthiazol-5-yl)benzyl)carbamoyl)pyrrolidin-1-yl)-3,3-dimethyl-1-oxobutan-2-yl)-N^{12}-(2-(4-((4'-(4-methylpiperazin-1-yl)-3'-(6-oxo-4-

(trifluoromethyl)-1,6-dihydropyridine-3-carboxamido)-[1,1'-biphenyl]-3-yl)methyl)piperazin-1-yl)ethyl)dodecanediamide (9).

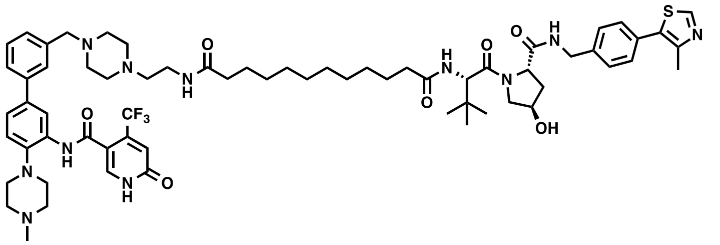

Compound **9** was synthesized following the standard procedures for preparing compound **1** from intermediate **21** (10 mg, 0.017 mmol), linker **30** (11 mg, 0.017 mmol, 1.0 equiv). Compound **9** was obtained as white solid in TFA salt form (11.6 mg, yield 56%). ^{1}H NMR (600 MHz, CD_3OD) δ 8.96 (s, 1H), 8.26 (d, J = 2.2 Hz, 1H), 8.03 (s, 1H), 7.74 (t, J = 1.9 Hz, 1H), 7.70 – 7.66 (m, 1H), 7.55 – 7.51 (m, 2H), 7.48 – 7.37 (m, 6H), 6.94 (s, 1H), 4.63 (s, 1H), 4.60 – 4.46 (m, 3H), 4.36 (d, J = 15.4 Hz, 1H), 4.18 (s, 2H), 3.92 – 3.86 (m, 1H), 3.80 (dd, J = 11.0, 3.9 Hz, 1H), 3.64 – 3.60 (m, 2H), 3.45 (t, J = 6.1 Hz, 2H), 3.34 – 3.11 (m, 14H), 3.01 (t, J = 6.2 Hz, 2H), 2.96 (s, 3H), 2.48 (s, 3H), 2.32 – 2.15 (m, 5H), 2.11 – 2.04 (m, 1H), 1.64 – 1.54 (m, 4H), 1.34 – 1.26 (m, 12H), 1.03 (s, 9H). t_R = 3.78 min, HRMS (m/z) for $C_{65}H_{87}F_3N_{11}O_7S^+$ $[M + H]^+$: calculated 1222.6457, found 1222.6482.

N-(3'-((4-(2-(5-((2-(2,6-dioxopiperidin-3-yl)-1,3-dioxoisoindolin-4-yl)amino)pentanamido)ethyl)piperazin-1-yl)methyl)-4-(4-methylpiperazin-1-yl)-[1,1'-biphenyl]-3-yl)-6-oxo-4-(trifluoromethyl)-1,6-dihydropyridine-3-carboxamide (10).

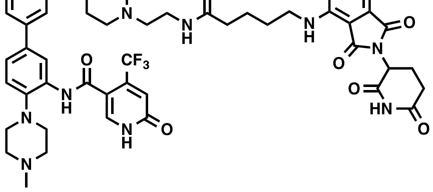

Compound **10** was synthesized following the standard procedures for preparing compound **1** from intermediate **21** (13.0 mg, 0.018 mmol), linker **31** (6.7 mg, 0.018 mmol, 1.0 equiv). Compound **10** was obtained as yellow solid in TFA salt form (12.2 mg, yield 71%). ^{1}H NMR (600 MHz, CD_3OD) δ 8.25 (d, *J* = 2.2 Hz, 1H), 8.02 (s, 1H), 7.72 (d, *J* = 1.8 Hz, 1H), 7.70 – 7.68 (m, 1H), 7.55 – 7.49 (m, 3H), 7.44 – 7.39 (m, 1H), 7.36 (d, *J* = 8.4 Hz, 1H), 7.02 (dd, *J* = 7.8, 4.2 Hz, 2H), 6.93 (s, 1H), 5.02 (dd, *J* = 12.5, 5.5 Hz, 1H), 4.15 (s, 2H), 3.61 (d, *J* = 11.7 Hz, 2H), 3.42 (t, *J* = 6.0 Hz, 2H), 3.36 – 3.31 (m, 3H), 3.27 (s, 3H), 3.22 – 3.00 (m, 9H), 2.96 (s, 3H), 2.89 (t, *J* = 5.9 Hz, 2H), 2.84 – 2.73 (m, 1H), 2.73 – 2.61 (m, 3H), 2.26 (t, *J* = 7.1 Hz, 2H), 2.10 – 2.02 (m, 1H), 1.76 – 1.61 (m, 4H). t_R = 3.56 min, HRMS (m/z) for $C_{49}H_{56}F_3N_{10}O_7^+$ $[M + H]^+$: calculated 953.4280, found 953.4276.

N-(3'-((4-(1-((2-(2,6-dioxopiperidin-3-yl)-1,3-dioxoisoindolin-4-yl)amino)-15-oxo-3,6,9,12-tetraoxa-16-azaoctadecan-18-yl)piperazin-1-yl)methyl)-4-(4-methylpiperazin-1-yl)-[1,1'-biphenyl]-3-yl)-6-oxo-4-(trifluoromethyl)-1,6-dihydropyridine-3-carboxamide (11).

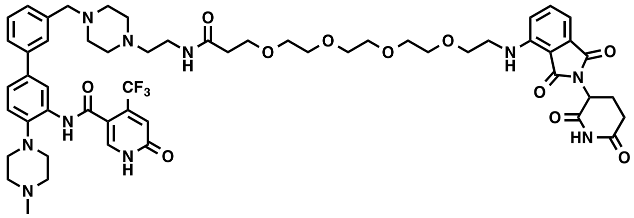

Compound **11** was synthesized following the standard procedures for preparing compound **1** from intermediate **21** (13.0 mg, 0.018 mmol), linker **32** (9.4 mg, 0.018 mmol, 1.0 equiv). Compound **11** was obtained as yellow solid in TFA salt form (14.4 mg, yield 73%). ^{1}H NMR (600 MHz, CD_3OD) δ 8.23 (d, *J* = 2.2 Hz, 1H), 8.03 (s, 1H), 7.72 (d, *J* = 1.8 Hz, 1H), 7.70 – 7.64 (m, 1H), 7.53 – 7.46 (m, 3H), 7.43 – 7.39 (m, 1H), 7.35 (d, *J* = 8.4 Hz, 1H), 7.01 (dd, *J* = 7.8, 4.8 Hz, 2H), 6.93 (s, 1H), 5.04 (dd, *J* = 12.8, 5.4 Hz, 1H), 4.19 – 4.11 (m, 2H), 3.74 – 3.65

(m, 4H), 3.64 – 3.53 (m, 14H), 3.52 – 3.46 (m, 2H), 3.42 (t, *J* = 5.2 Hz, 2H), 3.30 – 3.24 (m, 7H), 3.24 – 3.11 (m, 6H), 3.08 (t, *J* = 5.7 Hz, 2H), 2.96 (s, 3H), 2.87 – 2.78 (m, 1H), 2.74 – 2.64 (m, 3H), 2.45 (t, *J* = 5.8 Hz, 2H), 2.11 – 2.04 (m, 1H). t_R = 3.59 min, HRMS (m/z) for $C_{55}H_{68}F_3N_{10}O_{11}^+$ $[M + H]^+$: calculated 1101.5016, found 1101.5022.

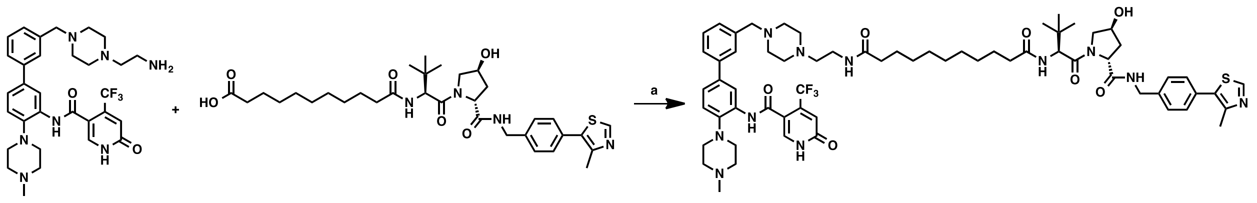

21 33 MS33N

Scheme S2. Reagents and Conditions: (a) EDCI, HOAt, NMM, DMSO, rt, 12 h.

N^1^-((S)-1-((2R,4S)-4-Hydroxy-2-((4-(4-methylthiazol-5-yl)benzyl)carbamoyl)pyrrolidin-1-yl)-3,3-dimethyl-1-oxobutan-2-yl)-N^11^-(2-(4-((4'-(4-methylpiperazin-1-yl)-3'-(6-oxo-4-(trifluoromethyl)-1,6-dihydropyridine-3-carboxamido)-[1,1'-biphenyl]-3-yl)methyl)piperazin-1-yl)ethyl)undecanediamide (MS33N). **MS33N** was synthesized following the standard procedures for preparing compound **1** from compound **21** (12 mg, 0.02 mmol) and linker **33**(*48*) (12.5 mg, 0.02 mmol, 1.0 equiv). **MS33N** was obtained as white solid in TFA salt form (20.5 mg, yield 85%). ^{1}H NMR (600 MHz, CD_3OD) δ 8.99 (s, 1H), 8.26 (d, *J* = 2.1 Hz, 1H), 8.02 (s, 1H), 7.76 (s, 1H), 7.71 (d, *J* = 7.8 Hz, 1H), 7.57 – 7.49 (m, 2H), 7.48 – 7.41 (m, 3H), 7.37 (dd, *J* = 13.5, 8.2 Hz, 3H), 6.93 (s, 1H), 4.62 – 4.54 (m, 1H), 4.54 – 4.46 (m, 2H), 4.44 – 4.39 (m, 1H), 4.34 (d, *J* = 15.7 Hz, 1H), 4.26 (s, 2H), 4.01 – 3.95 (m, 1H), 3.73 (dd, *J* = 10.9, 3.4 Hz, 1H), 3.61 (d, *J* = 11.8 Hz, 2H), 3.48 (t, *J* = 6.0 Hz, 2H), 3.43 – 3.29 (m, 12H), 3.20 – 3.07 (m, 4H), 2.96 (s, 3H), 2.49 (s, 3H), 2.30 – 2.22 (m, 1H), 2.22 – 2.10 (m, 4H), 2.06 – 1.96 (m, 1H), 1.60 – 1.48 (m, 2H), 1.47 – 1.28 (m, 2H), 1.27 – 1.12 (m, 10H), 1.07 (s, 9H).^{13}C NMR (201 MHz, CD_3OD) δ

176.08, 175.58, 173.15, 171.24, 163.89, 162.52, 151.87, 146.83, 142.32, 141.0, 140.08 (q, ${}^{2}J_{C\text{-}F}$ = 34.2 Hz, 1C, -*C-CF_3), 139.09, 138.33, 137.47, 132.65, 129.64, 129.53, 129.45, 129.00 (2C), 128.92, 127.83, 127.30 (2C), 124.45, 122.11, 121.02 (q, ${}^{1}J_{C\text{-}F}$ = 275.4 Hz, 1C, -*CF_3), 121.00, 119.23, 116.93, 115.49, 113.05, 69.12, 60.20, 59.63, 58.72, 56.29, 55.44, 53.60 (2C), 49.75, 49.21, 48.92 (2C), 42.31, 42.10, 37.72, 35.48, 34.89, 34.41, 33.94, 29.00 (2C), 28.93 (3C), 28.88 (2C), 25.67 (3C), 25.38, 25.23, 14.26. t_R = 3.68 min, HRMS (m/z) for $C_{64}H_{85}F_3N_{11}O_7S^+$ [M + H]$^+$: calculated 1208.6301, found 1208.6318.

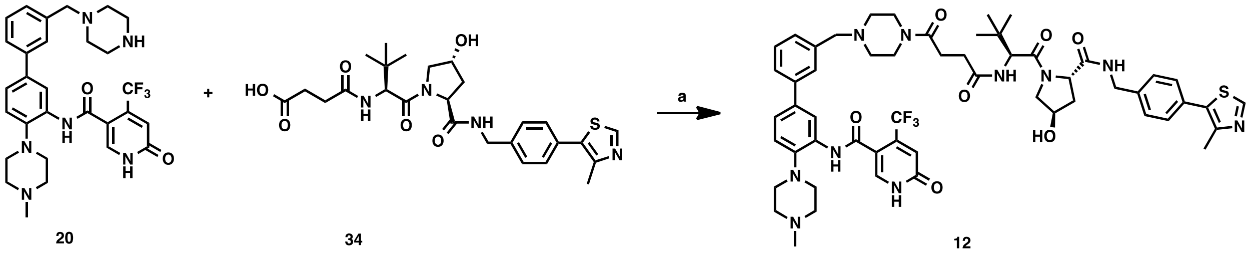

Scheme S3. Reagents and Conditions: (a) EDCI, HOAt, NMM, DMSO, rt, 12 h.

N-(3'-((4-(4-(((S)-1-((2S,4R)-4-hydroxy-2-((4-(4-methylthiazol-5-yl)benzyl)carbamoyl)pyrrolidin-1-yl)-3,3-dimethyl-1-oxobutan-2-yl)amino)-4-oxobutanoyl)piperazin-1-yl)methyl)-4-(4-methylpiperazin-1-yl)-[1,1'-biphenyl]-3-yl)-6-oxo-4-(trifluoromethyl)-1,6-dihydropyridine-3-carboxamide (12).

Compound **12** was synthesized following the standard procedures for preparing compound **1** from intermediate **20** (22.2 mg, 0.04 mmol), linker **34**(*48*) (21.2 mg, 0.04 mmol, 1.0 equiv). Compound **12** was obtained as white solid in TFA salt form (27.7 mg, yield 65%). ^{1}H NMR (600 MHz, CD_3OD) δ 9.06 (s, 1H), 8.31 (d, *J* = 2.2 Hz, 1H), 8.05 (s, 1H), 7.85 – 7.78 (m, 2H), 7.64 – 7.55 (m, 2H), 7.55-7.51 (m, 1H), 7.51 – 7.46 (m, 2H), 7.46 – 7.40 (m, 3H), 6.96 (s, 1H), 4.60 (s, 1H), 4.59 – 4.54 (m, 1H), 4.53 – 4.46 (m, 4H), 4.38 (d, *J* = 15.5 Hz, 1H), 3.93 – 3.86 (m, 1H),

3.80 (dd, J = 11.0, 3.9 Hz, 1H), 3.64 (d, J = 11.8 Hz, 2H), 3.55 – 3.34 (m, 6H), 3.32 – 3.25 (m, 6H), 3.24 – 3.15 (m, 2H), 2.98 (s, 3H), 2.79 – 2.55 (m, 4H), 2.50 (s, 3H), 2.27 – 2.20 (m, 1H), 2.13 – 2.06 (m, 1H), 1.04 (s, 9H). t_R = 3.64 min, HRMS (m/z) for $C_{55}H_{66}F_3N_{10}O_7S^+$ $[M + H]^+$: calculated 1067.4783, found 1067.4792.

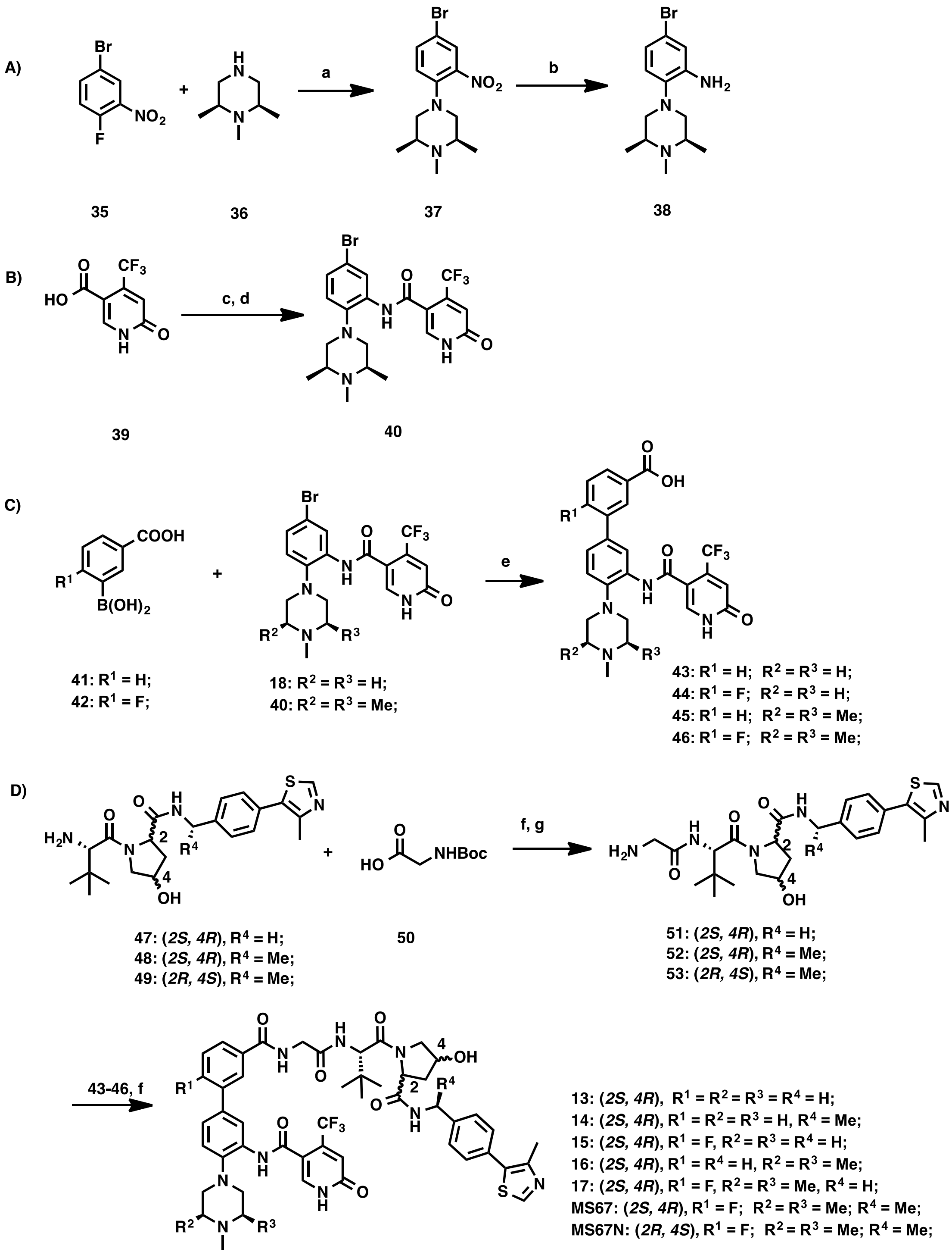

Scheme S4. Reagents and Conditions: (a) Et_3N, EtOH, 80 °C, 2 h; (b) $SnCl_2$, EtOH, reflux, 12 h; (c) $SOCl_2$, 60 °C, 3 h; (d) **38**, pyridine, DCM, 0 °C to rt, 12 h; (e) XPhos Pd G2, XPhos, Na_2CO_3,

dioxane/H_2O (5 : 3), MW, 120 °C, 1 h; (f) EDCI, HOAt, NMM, DMSO, rt, 12 h; (g) TFA/DCM, rt, 1 h.

5-bromo-2-((3S,5R)-3,4,5-trimethylpiperazin-1-yl)aniline (38)

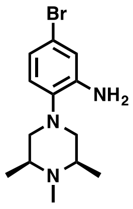

To a solution of 4-bromo-1-fluoro-2-nitrobenzene (**35**, 1.72 g, 7.81 mmol) and (2*S*,6*R*)-1,2,6-trimethylpiperazine (**36**, 1 g, 7.81 mmol) in EtOH (30 mL) was added triethylamine (946 mg, 9.37 mmol, 3 equiv). The reaction was stirring at 80 °C for 2 h and solvent was removed under reduced pressure. The crude product was purified by flash column chromatography (DCM/MeOH = 9/1) to get product (2*S*,6*R*)-4-(4-bromo-2-nitrophenyl)-1,2,6-trimethylpiperidine (**37**) as red oil (2.56 g, yield 99%). Compound **37** (2.56 g, 7.81 mmol) was dissolved in EtOH (50 mL). Tin chloride (3.52, 15.56 mmol, 2 equiv) was added and the reaction was refluxing overnight till compound **37** completely consumed. 6 N NaOH aqueous solution was added and extracted with EtOAc (3 x 50 mL). Combined organic layers were washed with saturated NaCl, dried over Na_2SO_4, filtered and evaporated. The resulting mixture was purified with flash column chromatography (DCM/MeOH = 9/1 to 4/1) to yield title compound **38** as orange solid (2.2 g, yield 95%). ^{1}H NMR (600 MHz, DMSO-d_6) δ 6.86 (d, J = 2.4 Hz, 1H), 6.83 (d, J = 8.4 Hz, 1H), 6.68 (dd, J = 8.3, 2.4 Hz, 1H), 3.56 – 3.48 (m, 2H), 3.21 – 3.14 (m, 2H), 2.86 (d, J = 4.6 Hz, 3H), 2.74 – 2.64 (m, 2H), 1.32 (d, J = 6.4 Hz, 6H). t_R = 3.60 min, HRMS (m/z) for $C_{13}H_{21}BrN_3^+$ $[M + H]^+$: calculated 298.0913, found 298.09 41.

N*-(5-bromo-2-((3*S*,5*R*)-3,4,5-trimethylpiperazin-1-yl)phenyl)-6-oxo-4-(trifluoromethyl)-1,6-dihydropyridine-3-carboxamide *(40)

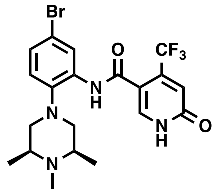

6-oxo-4-(trifluoromethyl)-1,6-dihydropyridine-3-carboxylic acid (**39**, 1 g, 4.8 mmol) was dissolved in $SOCl_2$ (10 mL) and the mixture was heated to 60 °C for 3 h. The solvent was removed and dried in vacuum to yield white intermediate. This intermediate was dissolved in DCM (10 mL) and the resulting mixture was added slowly to the suspension of compound **38** (2.1 g, 7.2 mmol, 1.5 equiv) and pyridine (1.1 g, 14.4 mmol, 3 equiv) in DCM (60 mL) at 0 °C. The reaction was warmed to room temperature slowly and stirred overnight. The solvent was removed and the mixture was purified by reverse phase column chromatography to afford title compound **40** as white solid (1.6 g, yield 68%). ^{1}H NMR (600 MHz, DMSO-d_6) δ 9.61 (s, 1H), 9.45 (s, 1H), 8.19 (d, *J* = 2.4 Hz, 1H), 7.98 (s, 1H), 7.37 (dd, *J* = 8.5, 2.4 Hz, 1H), 7.18 (d, *J* = 8.6 Hz, 1H), 6.84 (s, 1H), 3.54 – 3.44 (m, 2H), 3.30 – 3.22 (m, 2H), 2.86 (d, *J* = 4.2 Hz, 3H), 2.84 – 2.79 (m, 2H), 1.31 (d, *J* = 6.4 Hz, 6H). t_R = 3.80 min, HRMS (m/z) for $C_{20}H_{23}BrF_3N_4O_2^+$ $[M + H]^+$: calculated 487.0951, found 487.0972.

4'-(4-methylpiperazin-1-yl)-3'-(6-oxo-4-(trifluoromethyl)-1,6-dihydropyridine-3-carboxamido)-[1,1'-biphenyl]-3-carboxylic acid (43).

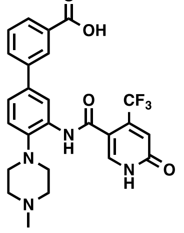

To a solution of compound **18** (230 mg, 0.5 mmol) and commercially available 3-boronobenzoic acid (**41**, 249 mg, 1.5 mmol, 3.0 euqiv) in 8 mL of 1,4-dioxane / H_2O (5 : 3) were added sodium carbonate (530 mg, 5 mmol, 10 equiv), XPhos (48 mg, 0.1 mmol, 0.2 equiv), and XPhos Pd G2 (79 mg, 0.1 mmol, 0.2 equiv). The reaction was heated to 120 °C for 1 h under microwave. The solvent was removed and purified by reverse phase ISCO (10% – 100% methanol / 0.1% TFA in H_2O) to afford compound **43** as white solid in TFA salt form (162 mg, yield 65%). ^{1}H NMR (600 MHz, DMSO-d_6) δ 12.63 (s, 1H), 9.94 (s, 1H), 9.60 (s, 1H), 8.23 (d, *J* = 2.2 Hz, 1H), 8.15 (d, *J* = 1.9 Hz, 1H), 8.05 (s, 1H), 7.95 (d, *J* = 7.7 Hz, 1H), 7.90 – 7.84 (m, 1H), 7.62 (t, *J* = 7.7 Hz, 1H), 7.55 (dd, *J* = 8.3, 2.3 Hz, 1H), 7.34 (d, *J* = 8.3 Hz, 1H), 6.84 (s, 1H), 3.59 – 3.48 (m, 2H), 3.32 – 3.17 (m, 4H), 3.05 (t, *J* = 12.1 Hz, 2H), 2.87 (s, 3H). t_R = 3.60 min, HRMS (m/z) for $C_{25}H_{24}F_3N_4O_4^+$ $[M + H]^+$: calculated 501.1744, found 501.1768.

6-fluoro-4'-(4-methylpiperazin-1-yl)-3'-(6-oxo-4-(trifluoromethyl)-1,6-dihydropyridine-3-carboxamido)-[1,1'-biphenyl]-3-carboxylic acid (44).

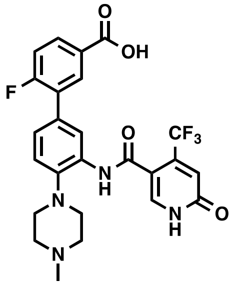

Compound **44** was synthesized following the standard procedures for preparing compound **43** from intermediate **18** (230 mg, 0.5 mmol), commercially available 3-borono-4-fluorobenzoic acid (**42**, 276 mg, 1.5 mmol, 3.0 equiv). Compound **44** was obtained as white solid in TFA salt form (142 mg, yield 55%). ^{1}H NMR (600 MHz, DMSO-d_6) δ 12.52 (s, 1H), 9.51 (s, 1H), 8.04 (d, *J* = 2.1 Hz, 1H), 7.99 – 7.94 (m, 2H), 7.94 – 7.89 (m, 1H), 7.42 – 7.33 (m, 3H), 7.27 (s, 1H),

6.76 (s, 1H), 3.51 – 2.95 (m, 8H), 2.73 (s, 3H). t_R = 3.70 min, HRMS (m/z) for $C_{25}H_{23}F_4N_4O_4^+$ $[M + H]^+$: calculated 519.1650, found 519.1643.

3'-(6-oxo-4-(trifluoromethyl)-1,6-dihydropyridine-3-carboxamido)-4'-((3S,5R)-3,4,5-trimethylpiperazin-1-yl)-[1,1'-biphenyl]-3-carboxylic acid (45)

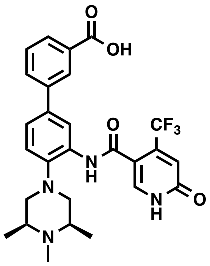

Compound **45** was synthesized following the standard procedures for preparing compound **43** from intermediate **40** (244 mg, 0.5 mmol), 3-boronobenzoic acid (**41**, 249 mg, 1.5 mmol, 3.0 equiv). Compound **45** was obtained as white solid in TFA salt form (104 mg, yield 39%). ^1H NMR (600 MHz, DMSO-d_6) δ 12.58 (s, 1H), 9.52 (s, 1H), 9.33 (s, 1H), 8.20 (d, J = 2.2 Hz, 1H), 8.08 (d, J = 2.0 Hz, 1H), 7.95 (s, 1H), 7.87 (d, J = 7.7 Hz, 1H), 7.83 – 7.76 (m, 1H), 7.55 (t, J = 7.7 Hz, 1H), 7.47 (dd, J = 8.3, 2.3 Hz, 1H), 7.25 (d, J = 8.3 Hz, 1H), 6.77 (s, 1H), 3.26 (d, J = 12.7 Hz, 2H), 2.86 – 2.77 (m, 4H), 2.43 (s, 3H), 1.26 (d, J = 6.4 Hz, 6H). t_R = 3.68 min, HRMS (m/z) for $C_{27}H_{28}F_3N_4O_4^+$ $[M + H]^+$: calculated 529.2057, found 529.2034.

6-Fluoro-3'-(6-oxo-4-(trifluoromethyl)-1,6-dihydropyridine-3-carboxamido)-4'-((3S,5R)-3,4,5-trimethylpiperazin-1-yl)-[1,1'-biphenyl]-3-carboxylic acid (46)

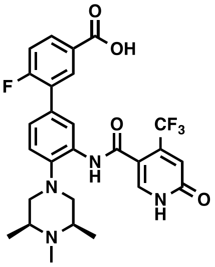

Compound **46** was synthesized following the standard procedures for preparing compound **43** from intermediate **40** (294 mg, 0.6 mmol), 3-borono-4-fluorobenzoic acid (**42**, 333 mg, 1.8 mmol, 3.0 equiv). Compound **46** was obtained as white solid in TFA salt form (161 mg, yield 63%). ^{1}H NMR (600 MHz, CD_3OD) δ 8.15 (dd, *J* = 7.6, 2.3 Hz, 2H), 8.06 – 8.03 (m, 1H), 8.02 (s, 1H), 7.47 – 7.43 (m, 1H), 7.38 (d, *J* = 8.3 Hz, 1H), 7.30 (dd, *J* = 10.3, 8.6 Hz, 1H), 6.91 (s, 1H), 3.61 – 3.49 (m, 2H), 3.36 – 3.31 (m, 3H), 3.03 – 2.99 (m, 1H), 2.98 (s, 3H), 1.44 (d, *J* = 6.5 Hz, 6H). t_R = 4.13 min, HRMS (m/z) for $C_{27}H_{27}F_4N_4O_4^+$ $[M + H]^+$: calculated 547.1963, found 547.1989.

(2S,4R)-1-((S)-2-(2-aminoacetamido)-3,3-dimethylbutanoyl)-4-hydroxy-N-(4-(4-methylthiazol-5-yl)benzyl)pyrrolidine-2-carboxamide (51).

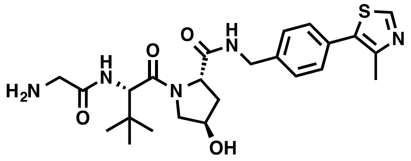

To the solution of commercially available (2*S*,4*R*)-1-((*S*)-2-amino-3,3-dimethylbutanoyl)-4-hydroxy-*N*-(4-(4-methylthiazol-5-yl)benzyl)pyrrolidine-2-carboxamide (**47**, 860 mg, 2 mmol) in DMSO (10 mL) were added commercially available (*tert*-butoxycarbonyl)glycine (**50**, 350 mg, 2 mmol, 2 equiv), EDCI (580 mg, 3 mmol, 1.5 equiv), HOAt (410 mg, 3 mmol, 1.5 equiv), and NMM (610 mg, 6 mmol, 3 equiv). After stirring overnight at room temperature, the resulting mixture was purified by reverse phase ISCO (10% – 100% methanol / 0.1% TFA in H_2O) to afford an intermediate. This intermediate was dissolved in TFA (10 mL) and DCM (10 mL) and

the resulting mixture was stirring for 1 h. the solvent was removed and the mixture was purified by reverse phase ISCO (10% – 100% methanol / 0.1% TFA in H_2O) to afford compound **51** as white solid in TFA salt form (564 mg, 58% yield for two steps). ^{1}H NMR (600 MHz, CD_3OD) δ 8.95 (s, 1H), 7.53 – 7.39 (m, 4H), 4.67 (s, 1H), 4.61 – 4.50 (m, 3H), 4.38 (d, *J* = 15.4 Hz, 1H), 3.98 – 3.92 (m, 1H), 3.86 – 3.69 (m, 3H), 2.50 (s, 3H), 2.28-2.19 (m, 1H), 2.14-2.08 (m, 1H), 1.08 (s, 9H). t_R = 3.47 min, HRMS *m/z* $[M+H]^+$ calculated for $C_{24}H_{34}N_5O_4S^+$ 488.2326, found 488.2358.

(2S,4R)-1-((S)-2-(2-Aminoacetamido)-3,3-dimethylbutanoyl)-4-hydroxy-N-((S)-1-(4-(4-methylthiazol-5-yl)phenyl)ethyl)pyrrolidine-2-carboxamide (52)

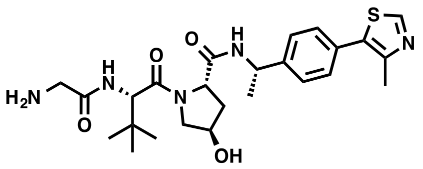

Compound **52** was synthesized following the standard procedures for preparing compound **51** from (2*S*,4*R*)-1-((*S*)-2-amino-3,3-dimethylbutanoyl)-4-hydroxy-*N*-((*S*)-1-(4-(4-methylthiazol-5-yl)phenyl)ethyl)pyrrolidine-2-carboxamide (**48**, 86 mg, 0.2 mmol, prepared according to the published procedures (*37*)), (*tert*-butoxycarbonyl)glycine (**50**, 35 mg, 0.2 mmol, 1 equiv), EDCI (58 mg, 0.3 mmol, 1.5 equiv), HOAt (41 mg, 0.3 mmol, 1.5 equiv), and NMM (61 mg, 0.6 mmol, 3 equiv) in DMSO (1 mL). Compound **52** was obtained as white solid in TFA salt form (30.5 mg, 61% yield for two steps). ^{1}H NMR (600 MHz, CD_3OD) δ 9.03 (s, 1H), 7.46 – 7.41 (m, 4H), 5.02 – 4.98 (m, 1H), 4.95 (s, 1H), 4.89 (s, 1H), 4.65 (s, 1H), 4.60 – 4.52 (m, 1H), 4.44 (dd, *J* = 4.0, 2.2 Hz, 1H), 3.93 – 3.87 (m, 1H), 3.77 – 3.72 (m, 1H), 2.49 (s, 3H), 2.24 – 2.15 (m, 1H), 1.97 – 1.90 (m, 1H), 1.50 (d, *J* = 7.1 Hz, 3H), 1.06 (s, 9H). t_R = 4.13 min, HRMS (m/z) for $C_{25}H_{36}N_5O_4S^+$ $[M + H]^+$: calculated 502.2483, found 502.2444.

(2R,4S)-1-((S)-2-(2-Aminoacetamido)-3,3-dimethylbutanoyl)-4-hydroxy-N-((S)-1-(4-(4-methylthiazol-5-yl)phenyl)ethyl)pyrrolidine-2-carboxamide (53)

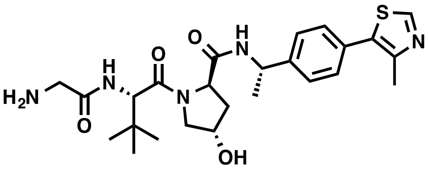

Compound **53** was synthesized following the standard procedures for preparing compound **51** from (2*R*,4*S*)-1-((*S*)-2-amino-3,3-dimethylbutanoyl)-4-hydroxy-*N*-((*S*)-1-(4-(4-methylthiazol-5-yl)phenyl)ethyl)pyrrolidine-2-carboxamide (**49**, 22.2 mg, 0.05 mmol, prepared according to the published procedures(*40*)), compound **50** (8.8 mg, 0.05 mmol, 1.0 equiv), EDCI (14.4 mg, 0.075 mmol, 1.5 equiv), HOAt (10.2 mg, 0.075 mmol, 1.5 equiv), and NMM (30.3 mg, 0.3 mmol, 3.0 equiv) in DMSO (1 mL). Compound **53** was obtained as white solid in TFA salt form (17.9 mg, 72% yield for two steps). ^{1}H NMR (600 MHz, CD_3OD) δ 9.23 (s, 1H), 7.61 – 7.42 (m, 4H), 4.67 (s, 1H), 4.55 (t, J = 7.7 Hz, 1H), 4.52 – 4.47 (m, 1H), 3.97 (s, 1H), 3.89 (dd, J = 10.8, 4.6 Hz, 1H), 3.73 – 3.65 (m, 3H), 2.52 (s, 3H), 2.31 – 2.15 (m, 1H), 2.14 – 2.00 (m, 1H), 1.48 (d, J = 7.1 Hz, 3H), 1.04 (s, 9H). t_R = 4.18 min, HRMS (m/z) for $C_{25}H_{36}N_5O_4S^+$ $[M + H]^+$: calculated 502.2483, found 502.2495**.**

N-(3'-((2-(((S)-1-((2S,4R)-4-hydroxy-2-((4-(4-methylthiazol-5-yl)benzyl)carbamoyl)pyrrolidin-1-yl)-3,3-dimethyl-1-oxobutan-2-yl)amino)-2-oxoethyl)carbamoyl)-4-(4-methylpiperazin-1-yl)-[1,1'-biphenyl]-3-yl)-6-oxo-4-(trifluoromethyl)-1,6-dihydropyridine-3-carboxamide (13).

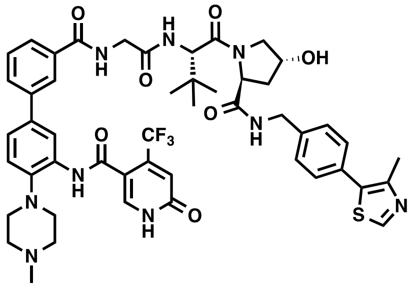

Compound **13** was synthesized following the standard procedures for preparing compound **1** from intermediate **43** (17.5 mg, 0.035 mmol), linker **51** (17 mg, 0.035 mmol, 1.0 equiv). Compound **13** was obtained as white solid in TFA salt form (20 mg, yield 59%). ^{1}H NMR (600 MHz, CD_3OD) δ 8.90 (s, 1H), 8.27 (d, *J* = 2.2 Hz, 1H), 8.17 (t, *J* = 1.8 Hz, 1H), 8.05 (s, 1H), 7.89-7.87 (m, 1H), 7.84-7.82 (m, 1H), 7.62 – 7.55 (m, 2H), 7.47 (d, *J* = 8.0 Hz, 2H), 7.42 – 7.37 (m, 3H), 6.96 (s, 1H), 4.69 (s, 1H), 4.63 – 4.48 (m, 3H), 4.35 (d, *J* = 15.5 Hz, 1H), 4.14 (d, *J* = 4.9 Hz, 2H), 3.92 (d, *J* = 11.0 Hz, 1H), 3.82 (dd, *J* = 11.0, 3.8 Hz, 1H), 3.64 (d, *J* = 11.9 Hz, 2H), 3.39 – 3.34 (m, 2H), 3.31 – 3.26 (m, 2H), 3.15 (t, *J* = 12.5 Hz, 2H), 2.99 (s, 3H), 2.46 (s, 3H), 2.28 – 2.18 (m, 1H), 2.13 – 2.05 (m, 1H), 1.06 (s, 9H). t_R = 3.80 min, HRMS (m/z) for $C_{49}H_{55}F_3N_9O_7S^+$ $[M + H]^+$: calculated 970.3892, found 970.3877.

N-(3'-((2-(((S)-1-((2S,4R)-4-hydroxy-2-(((S)-1-(4-(4-methylthiazol-5-yl)phenyl)ethyl)carbamoyl)pyrrolidin-1-yl)-3,3-dimethyl-1-oxobutan-2-yl)amino)-2-oxoethyl)carbamoyl)-4-(4-methylpiperazin-1-yl)-[1,1'-biphenyl]-3-yl)-6-oxo-4-(trifluoromethyl)-1,6-dihydropyridine-3-carboxamide (14).

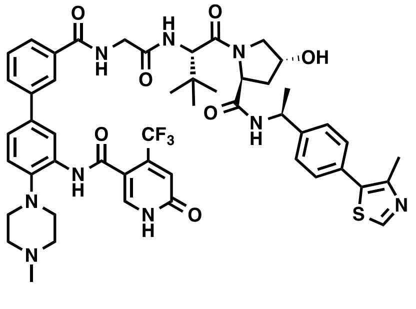

Compound **14** was synthesized following the standard procedures for preparing compound **1** from intermediate **43** (17.5 mg, 0.035 mmol), linker **52** (17.7 mg, 0.035 mmol, 1.0 equiv). Compound **14** was obtained as white solid in TFA salt form (28.6 mg, yield 83%). ^{1}H NMR (600 MHz, CD_3OD) δ 8.92 (s, 1H), 8.27 (d, *J* = 2.2 Hz, 1H), 8.17 (t, *J* = 1.8 Hz, 1H), 8.05 (s, 1H), 7.90-7.85 (m, 2H), 7.65 – 7.55 (m, 2H), 7.50 – 7.37 (m, 5H), 6.96 (s, 1H), 5.05 – 4.98 (m, 1H), 4.69 (s, 1H), 4.62 – 4.54 (m, 1H), 4.45 (s, 1H), 4.23 – 4.08 (m, 2H), 3.89 (d, *J* = 11.1 Hz, 1H), 3.77 (dd, *J* = 11.0, 3.9 Hz, 1H), 3.64 (d, *J* = 11.9 Hz, 2H), 3.39 – 3.35 (m, 2H), 3.32 – 3.27 (m, 2H), 3.16 (t, *J* = 12.0 Hz, 2H), 2.99 (s, 3H), 2.50 (s, 3H), 2.21 (dd, *J* = 13.2, 7.8 Hz, 1H), 2.02 – 1.93 (m, 1H), 1.51 (d, *J* = 7.0 Hz, 3H), 1.08 (s, 9H). t_R = 3.81 min, HRMS (m/z) for $C_{50}H_{57}F_3N_9O_7S^+$ $[M + H]^+$: calculated 984.4048, found 984.4067.

N-(2'-fluoro-5'-((2-(((S)-1-((2S,4R)-4-hydroxy-2-((4-(4-methylthiazol-5-yl)benzyl)carbamoyl)pyrrolidin-1-yl)-3,3-dimethyl-1-oxobutan-2-yl)amino)-2-oxoethyl)carbamoyl)-4-(4-methylpiperazin-1-yl)-[1,1'-biphenyl]-3-yl)-6-oxo-4-(trifluoromethyl)-1,6-dihydropyridine-3-carboxamide (15).

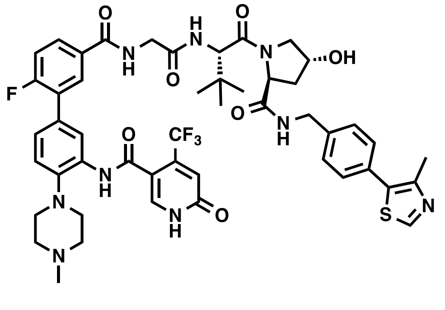

Compound **15** was synthesized following the standard procedures for preparing compound **1** from intermediate **44** (18.4 mg, 0.035 mmol), linker **51** (17 mg, 0.035 mmol, 1.0 equiv). Compound **15** was obtained as white solid in TFA salt form (26.5 mg, yield 77%). ^{1}H NMR (600 MHz, CD_3OD) δ 8.78 (s, 1H), 8.05 (s, 1H), 7.95 (dd, *J* = 7.4, 2.4 Hz, 1H), 7.92 (s, 1H), 7.85-

7.79 (m, 1H), 7.42-7.32 (m, 3H), 7.31 – 7.26 (m, 3H), 7.21 (dd, J = 10.3, 8.6 Hz, 1H), 6.83 (s, 1H), 4.57 (s, 1H), 4.51 – 4.36 (m, 3H), 4.24 (d, J = 15.5 Hz, 1H), 4.06 – 3.96 (m, 2H), 3.82 – 3.76 (m, 1H), 3.72 – 3.68 (m, 1H), 3.56 – 3.48 (m, 2H), 3.26 – 3.22 (m, 2H), 3.20 – 3.15 (m, 2H), 3.11 – 2.98 (m, 2H), 2.87 (s, 3H), 2.35 (s, 3H), 2.16 – 2.06 (m, 1H), 2.02 – 1.92 (m, 1H), 0.94 (s, 9H). t_R = 3.79 min, HRMS (m/z) for $C_{49}H_{54}F_4N_9O_7S^+$ $[M + H]^+$: calculated 988.3798, found 988.3808.

N-(3'-((2-(((S)-1-((2S,4R)-4-hydroxy-2-((4-(4-methylthiazol-5-yl)benzyl)carbamoyl)pyrrolidin-1-yl)-3,3-dimethyl-1-oxobutan-2-yl)amino)-2-oxoethyl)carbamoyl)-4-((3S,5R)-3,4,5-trimethylpiperazin-1-yl)-[1,1'-biphenyl]-3-yl)-6-oxo-4-(trifluoromethyl)-1,6-dihydropyridine-3-carboxamide (16).

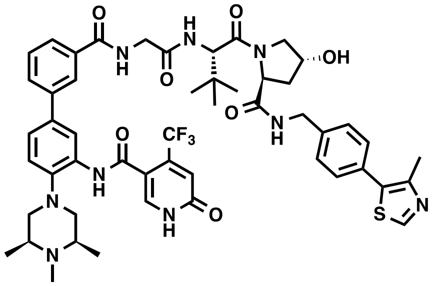

Compound **16** was synthesized following the standard procedures for preparing compound **1** from intermediate **45** (18.5 mg, 0.035 mmol), linker **51** (17 mg, 0.035 mmol, 1.0 equiv). Compound **16** was obtained as white solid in TFA salt form (21.2 mg, yield 89%). ^{1}H NMR (600 MHz, CD_3OD) δ 8.91 (s, 1H), 8.27 (d, J = 2.2 Hz, 1H), 8.17 (t, J = 1.8 Hz, 1H), 8.04 (s, 1H), 7.90 – 7.86 (m, 1H), 7.86 – 7.81 (m, 1H), 7.63 – 7.55 (m, 2H), 7.47 (d, J = 8.1 Hz, 2H), 7.43 – 7.36 (m, 3H), 6.96 (s, 1H), 4.69 (s, 1H), 4.62 – 4.48 (m, 3H), 4.35 (d, J = 15.5 Hz, 1H), 4.19 – 4.10 (m, 2H), 3.92 (d, J = 11.0 Hz, 1H), 3.82 (dd, J = 11.0, 3.8 Hz, 1H), 3.58 – 3.49 (m, 2H), 3.39 – 3.34 (m, 3H), 3.02 (s, 3H), 3.00 – 2.91 (m, 1H), 2.46 (s, 3H), 2.27 – 2.19 (m, 1H), 2.14 –

2.05 (m, 1H), 1.47 (dd, *J* = 6.5, 1.2 Hz, 6H), 1.06 (s, 9H). t_R = 3.81 min, HRMS (m/z) for $C_{51}H_{59}F_3N_9O_7S^+$ $[M + H]^+$: calculated 998.4205, found 998.4228.

N-(2'-fluoro-5'-((2-(((S)-1-((2S,4R)-4-hydroxy-2-((4-(4-methylthiazol-5-yl)benzyl)carbamoyl)pyrrolidin-1-yl)-3,3-dimethyl-1-oxobutan-2-yl)amino)-2-oxoethyl)carbamoyl)-4-((3S,5R)-3,4,5-trimethylpiperazin-1-yl)-[1,1'-biphenyl]-3-yl)-6-oxo-4-(trifluoromethyl)-1,6-dihydropyridine-3-carboxamide (17).

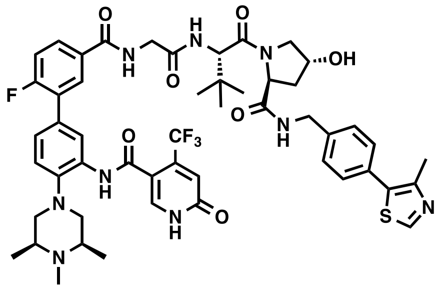

Compound **17** was synthesized following the standard procedures for preparing compound **1** from intermediate **46** (13 mg, 0.024 mmol), linker **51** (17.2 mg, 0.024 mmol, 1.0 equiv). Compound **17** was obtained as white solid in TFA salt form (21.2 mg, yield 89%). ^{1}H NMR (600 MHz, CD_3OD) δ 8.99 (s, 1H), 8.15 (s, 1H), 8.04 (dd, *J* = 7.4, 2.4 Hz, 1H), 8.00 (s, 1H), 7.93 – 7.87 (m, 1H), 7.46 (dd, *J* = 10.0, 7.3 Hz, 3H), 7.41 – 7.34 (m, 3H), 7.29 (dd, *J* = 10.3, 8.6 Hz, 1H), 6.91 (s, 1H), 4.66 (s, 1H), 4.60 – 4.47 (m, 3H), 4.34 (d, *J* = 15.5 Hz, 1H), 4.10 (d, *J* = 11.7 Hz, 2H), 3.89 (d, *J* = 11.2 Hz, 1H), 3.79 (dd, *J* = 11.0, 3.8 Hz, 1H), 3.56 – 3.48 (m, 2H), 3.32 – 3.27 (m, 4H), 2.98 (s, 3H), 2.46 (s, 3H), 2.25 – 2.17 (m, 1H), 2.10 – 2.01 (m, 1H), 1.44 (d, *J* = 6.5 Hz, 6H), 1.04 (s, 9H). t_R = 4.16 min, HRMS (m/z) for $C_{51}H_{58}F_4N_9O_7S^+$ $[M + H]^+$: calculated 1016.4111, found 1016.4124.

N*-(2'-fluoro-5'-((2-(((*S*)-1-((2*S*,4*R*)-4-hydroxy-2-(((*S*)-1-(4-(4-methylthiazol-5-yl)phenyl)ethyl)carbamoyl)pyrrolidin-1-yl)-3,3dimethyl-1-oxobutan-2-yl)amino)-2-oxoethyl)carbamoyl)-4-((3*S*,5*R*)-3,4,5-trimethylpiperazin-1-yl)-[1,1'-biphenyl]-3-yl)-6-oxo-4-(trifluoromethyl)-1,6-dihydropyridine-3-carboxamide *(MS67)

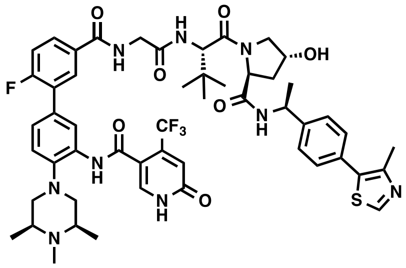

MS67 was synthesized following the standard procedures for preparing compound **1** from compound **46** (11 mg, 0.02 mmol), linker **52** (10 mg, 0.02 mmol, 1.0 equiv). **MS67** was obtained as white solid in TFA salt form (12.8 mg, yield 62%). ^{1}H NMR (600 MHz, CD_3OD) δ 8.88 (s, 1H), 8.18 (s, 1H), 8.06 (dd, *J* = 7.4, 2.4 Hz, 1H), 8.01 (s, 1H), 7.92 – 7.87 (m, 1H), 7.46 – 7.39 (m, 5H), 7.34 – 7.26 (m, 2H), 6.91 (s, 1H), 5.00 (q, *J* = 7.0 Hz, 1H), 4.68 (s, 1H), 4.62 – 4.56 (m, 1H), 4.47 – 4.42 (m, 1H), 4.20 – 4.05 (m, 2H), 3.90-3.88 (m, 1H), 3.76 (dd, *J* = 11.0, 3.8 Hz, 1H), 3.03 (dd, *J* = 11.9, 2.3 Hz, 2H), 2.70-2.66 (m, 2H), 2.63 – 2.54 (m, 2H), 2.48 (s, 3H), 2.40 (s, 3H), 2.25 – 2.16 (m, 1H), 2.00 – 1.93 (m, 1H), 1.49 (d, *J* = 7.0 Hz, 3H), 1.18 (d, *J* = 6.2 Hz, 6H), 1.07 (s, 9H). ^{13}C NMR (151 MHz, CD_3OD) δ 171.79, 170.62, 170.01, 167.83, 163.40, 163.17, 161.71 (d, $^{1}J_{C-F}$ = 253.7 Hz, 1C, -*C-F), 151.45, 147.66, 144.21, 143.64, 139.90 (q, $^{2}J_{C-F}$ = 33.2 Hz, 1C), 138.83, 132.01, 131.95, 130.94, 130.25 (d, $^{3}J_{C-F}$ = 3.0 Hz, 1C), 130.10, 129.99 (d, $^{3}J_{C-F}$ = 4.5 Hz, 1C), 129.08 (2C), 128.45, 128.43 (d, $^{2}J_{C-F}$ = 24.2 Hz, 1C, -*C-CF), 126.36, 126.24 (2C), 123.48, 122.04 (q, $^{1}J_{C-F}$ = 276.3 Hz, 1C, -*CF_3), 120.13, 118.81 (d, $^{3}J_{C-F}$ = 6.0 Hz, 1C), 116.09 (d, $^{2}J_{C-F}$ = 22.7 Hz, 1C, -*C-CF), 113.15, 69.60, 68.07, 59.25, 58.51, 58.26, 57.72, 56.65,

48.74, 42.73, 37.38, 36.53, 35.46, 29.76 (3C), 25.65, 25.59, 21.04, 16.14, 14.45. t_R = 3.95 min, HRMS (m/z) for $C_{52}H_{60}F_4N_9O_7S^+$ [M + H]$^+$: calculated 1030.4267, found 1030.4278.

N-(2'-Fluoro-5'-((2-(((S)-1-((2R,4S)-4-hydroxy-2-(((S)-1-(4-(4-methylthiazol-5-yl)phenyl)ethyl)carbamoyl)pyrrolidin-1-yl)-3,3-dimethyl-1-oxobutan-2-yl)amino)-2-oxoethyl)carbamoyl)-4-((3S,5R)-3,4,5-trimethylpiperazin-1-yl)-[1,1'-biphenyl]-3-yl)-6-oxo-4-(trifluoromethyl)-1,6-dihydropyridine-3-carboxamide (MS67N)

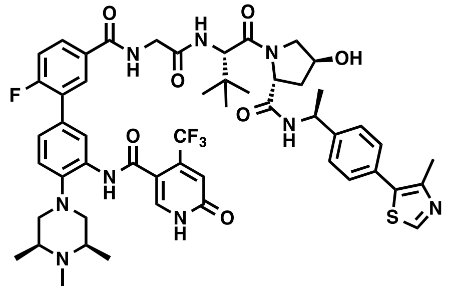

MS67N was synthesized following the standard procedures for preparing compound **1** from compound **46** (11 mg, 0.02 mmol), linker **53** (10 mg, 0.02 mmol, 1.0 equiv), EDCI (5.8 mg, 0.03 mmol, 1.5 equiv), HOAt (4.1 mg, 0.03 mmol, 1.5 equiv), and NMM (6.1 mg, 0.06 mmol, 3.0 equiv) in DMSO (1 mL). **MS67N** was obtained as white solid in TFA salt form (15.4 mg, yield 75%). ^{1}H NMR (600 MHz, CD_3OD) δ 9.05 (s, 1H), 8.12 (d, J = 2.0 Hz, 1H), 8.03 – 7.98 (m, 2H), 7.88 – 7.84 (m, 1H), 7.51 – 7.45 (m, 2H), 7.38 – 7.28 (m, 4H), 7.23 (dd, J = 10.3, 8.6 Hz, 1H), 6.93 (s, 1H), 5.02 (q, J = 7.0 Hz, 1H), 4.56 (dd, J = 8.3, 6.6 Hz, 1H), 4.51 (s, 1H), 4.46 – 4.40 (m, 1H), 4.18 – 4.07 (m, 2H), 3.96 (dd, J = 10.9, 5.0 Hz, 1H), 3.71 (dd, J = 10.7, 3.5 Hz, 1H), 3.58 – 3.48 (m, 2H), 3.32 – 3.30 (m, 1H), 3.29 – 3.28 (m, 1H), 3.00 (s, 3H), 2.97 (d, J = 10.0 Hz, 2H), 2.44 (s, 3H), 2.25-2.22 (m, 1H), 2.12-2.08 (m, 1H), 1.48 – 1.40 (m, 9H), 1.10 (s, 9H). ^{13}C NMR (151 MHz, CD_3OD) δ 171.94, 170.69, 170.41, 167.54, 163.80, 162.44, 161.64 (d, $^1J_{C-F}$ = 265.8 Hz, 1C, -*C-F), 152.01, 146.37, 144.25, 142.24, 140.03 (q, $^2J_{C-F}$ = 33.2 Hz, 1C, -*C-CF_3),

138.38, 132.71, 132.22, 132.00, 130.33, 130.05 (d, $^3J_{C-F}$ = 4.5 Hz, 1C), 129.26, 128.92 (2C), 128.55 (d, $^3J_{C-F}$ = 9.1 Hz, 1C), 128.05 (d, $^2J_{C-F}$ = 23.6 Hz, 1C, *C-CF), 126.95, 126.54 (2C), 124.27, 122. 01 (q, $^1J_{C-F}$ = 289.9 Hz, 1C, -$^*CF_3$), 120.54, 119.23 (d, $^3J_{C-F}$ = 4.5 Hz, 1C), 116.04 (d, $^2J_{C-F}$ = 24.2 Hz, 1C, *C-CF), 112.98, 69.06, 60.97, 59.46, 58.49, 56.18, 56.09, 55.52, 48.44, 42.40, 37.65, 35.98, 34.40, 25.61 (3C), 25.44, 21.30, 14.01, 13.74, 13.40. t_R = 3.85 min, HRMS (m/z) for $C_{52}H_{60}F_4N_9O_7S^+$ $[M + H]^+$: calculated 1030.4267, found 1030.4253.

HPLC-HRMS spectrum of compound 1.

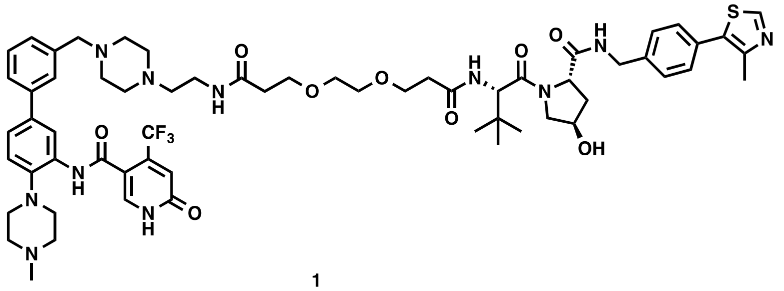

1

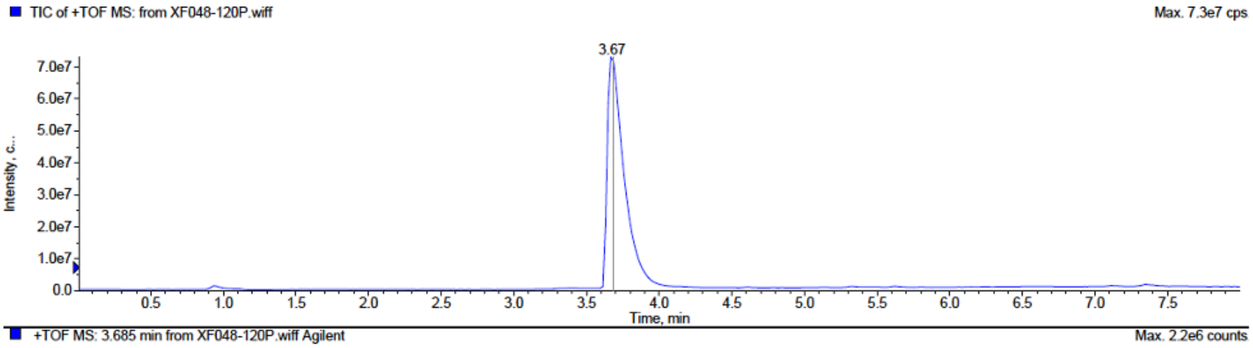

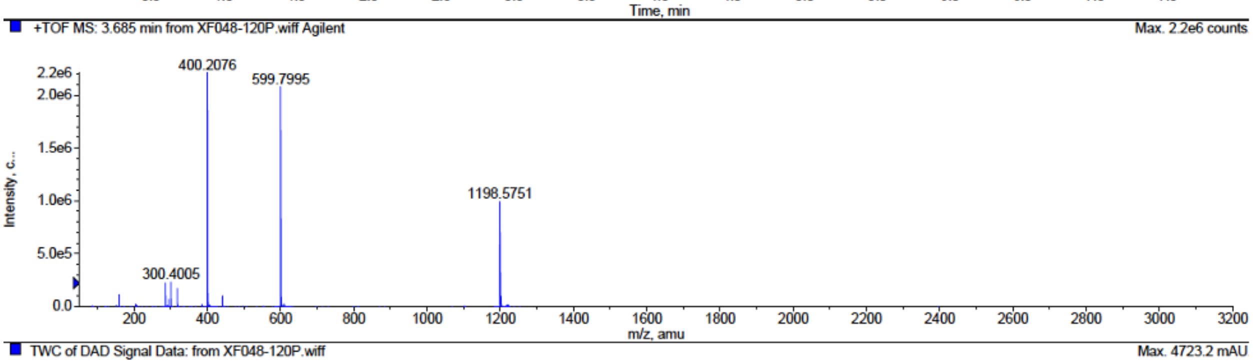

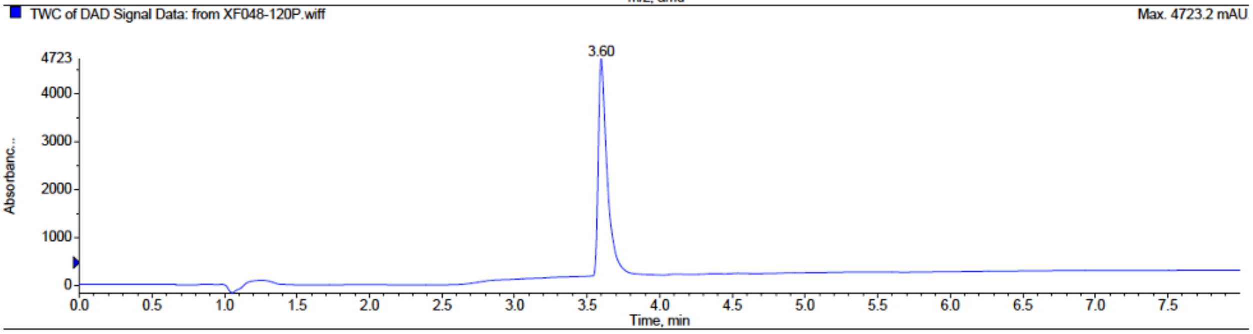

HPLC-HRMS spectrum of compound 2.

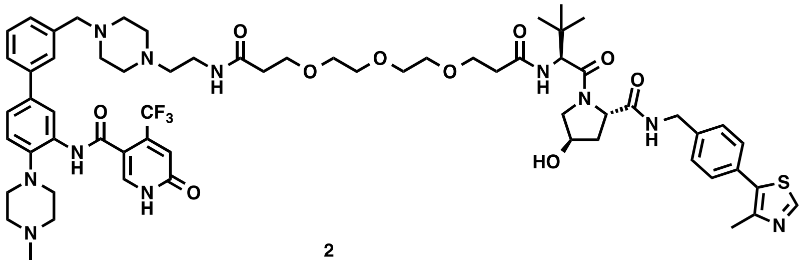

2

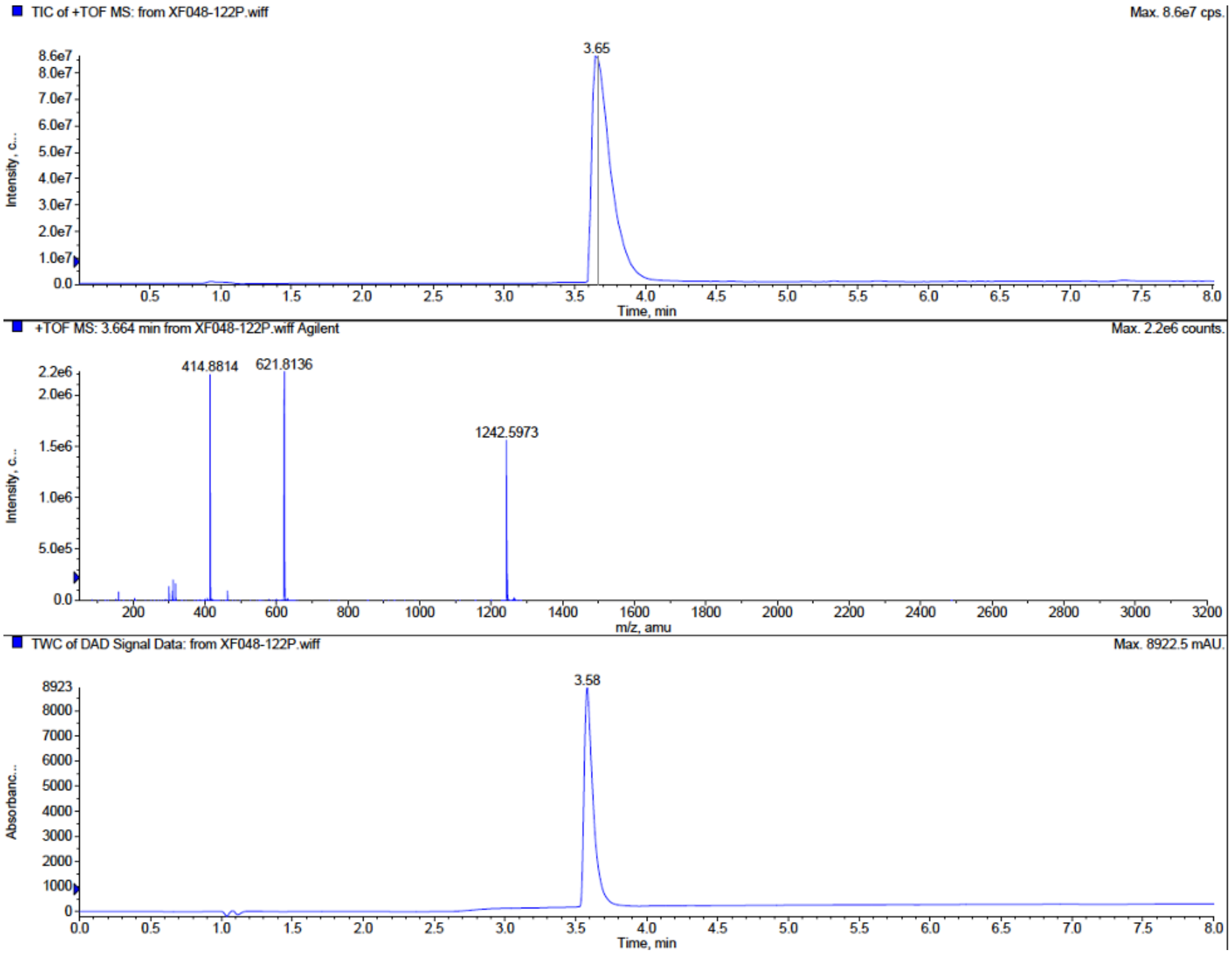

HPLC-HRMS spectrum of compound 3.

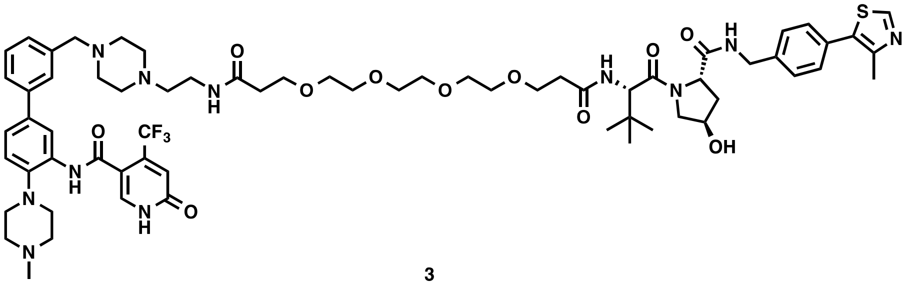

3

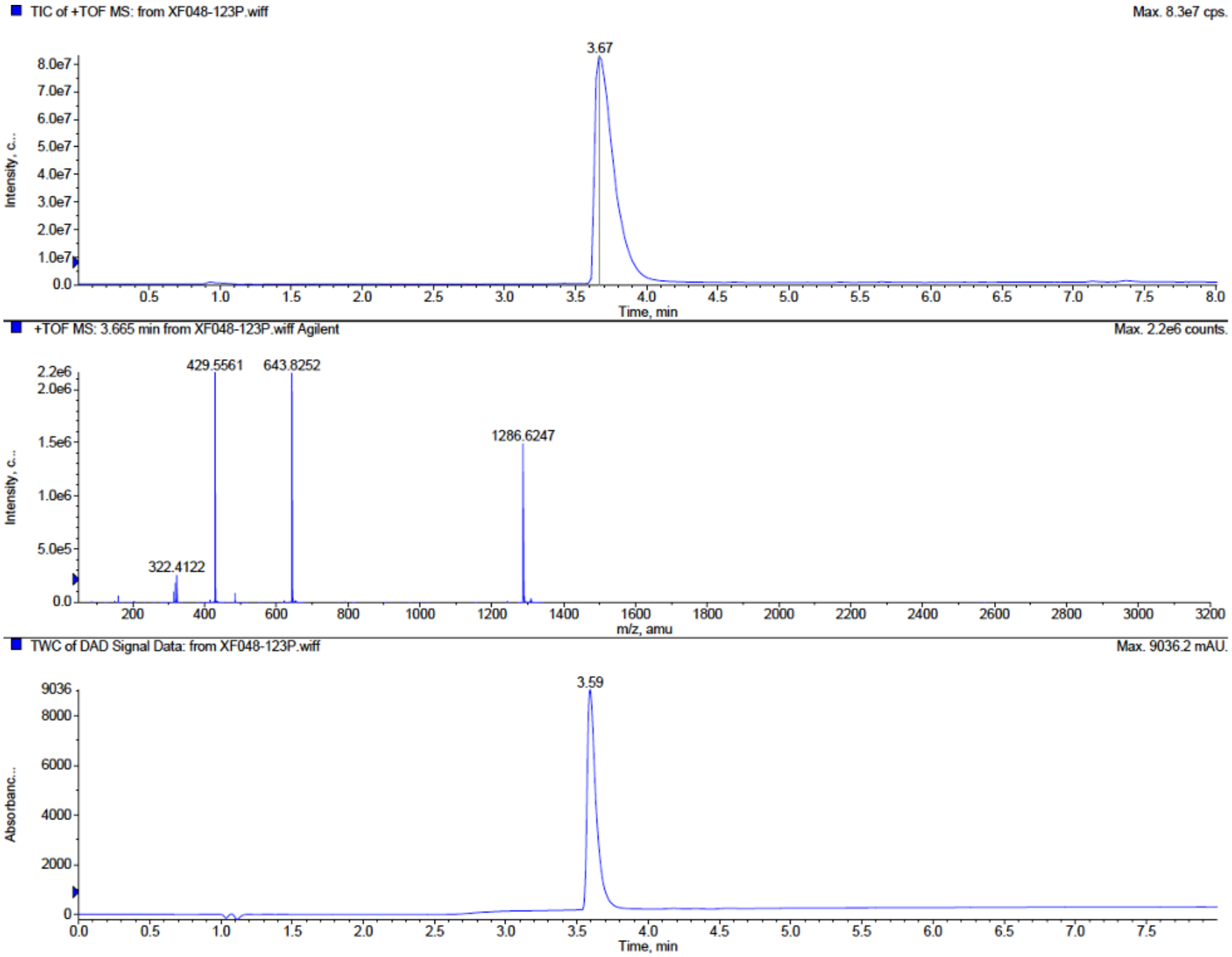

HPLC-HRMS spectrum of compound 4.

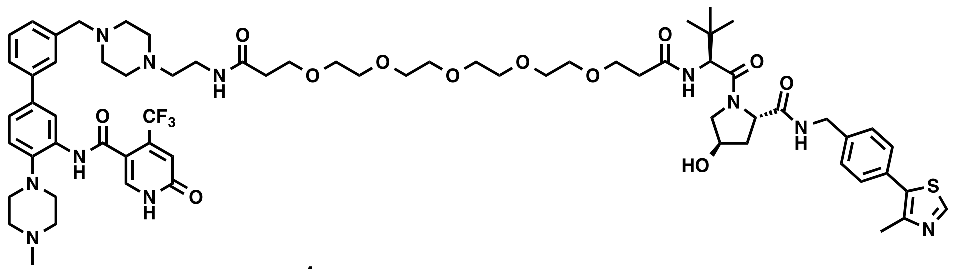

4

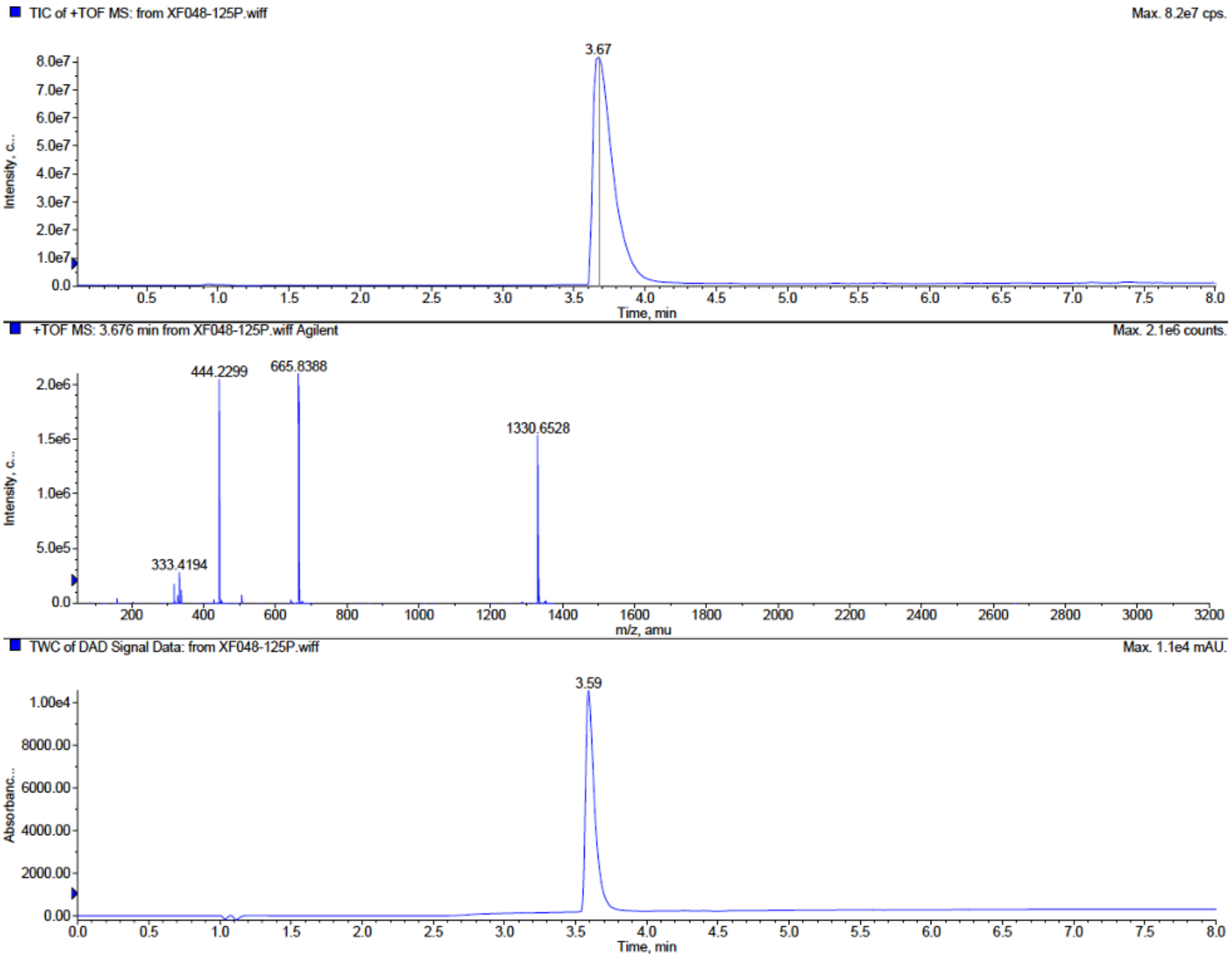

HPLC-HRMS spectrum of compound 5.

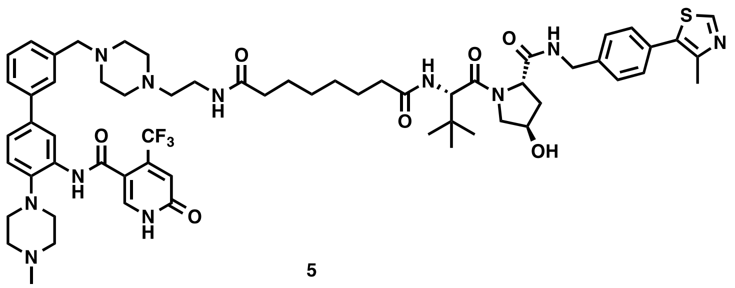

5

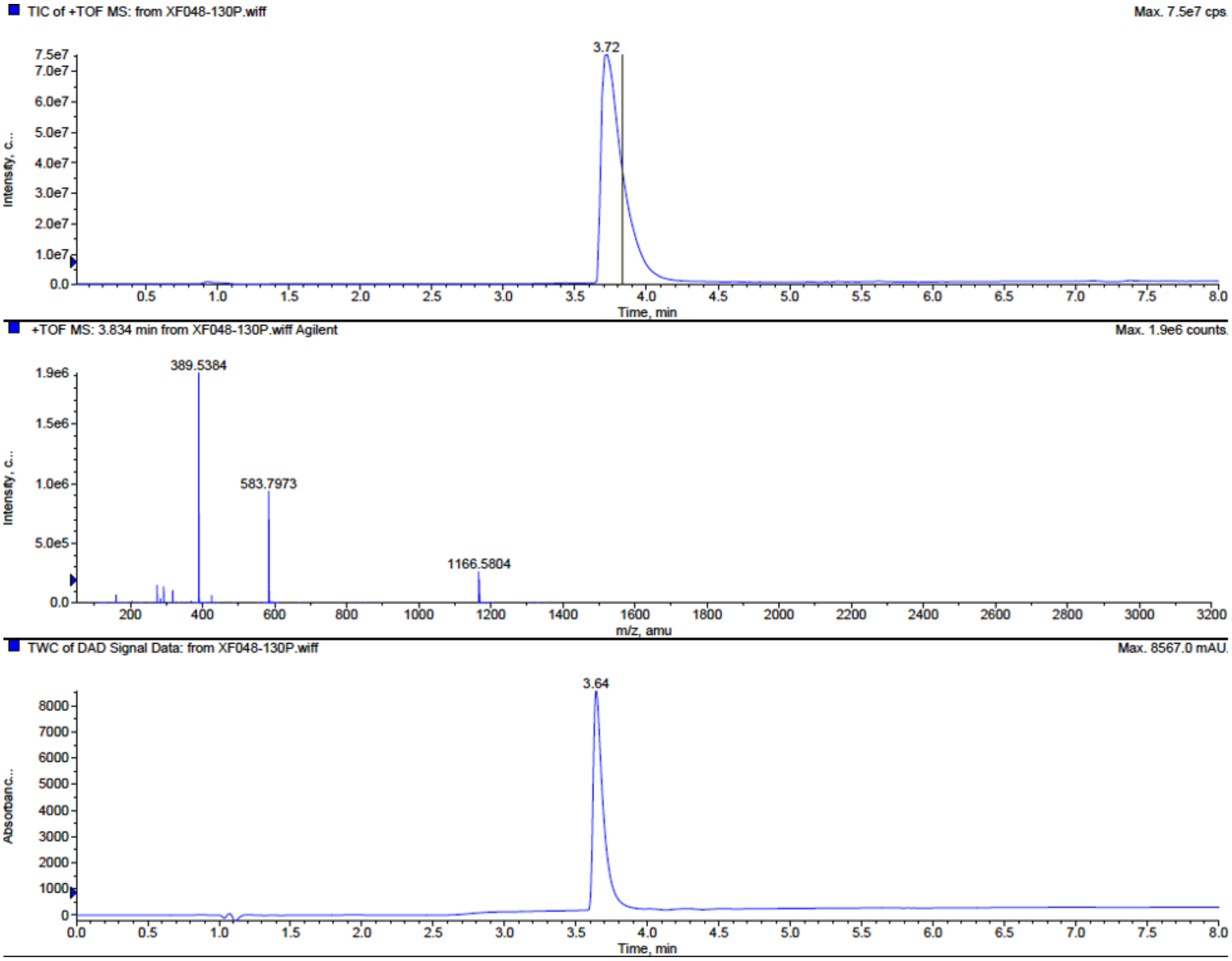

HPLC-HRMS spectrum of compound 6.

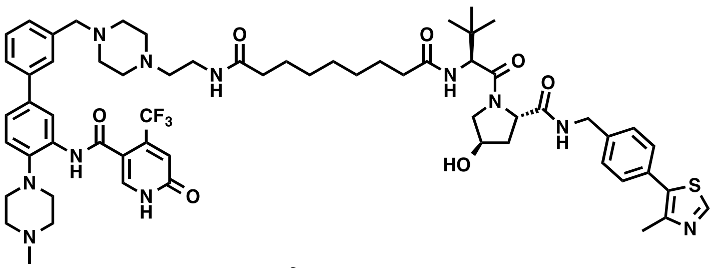

6

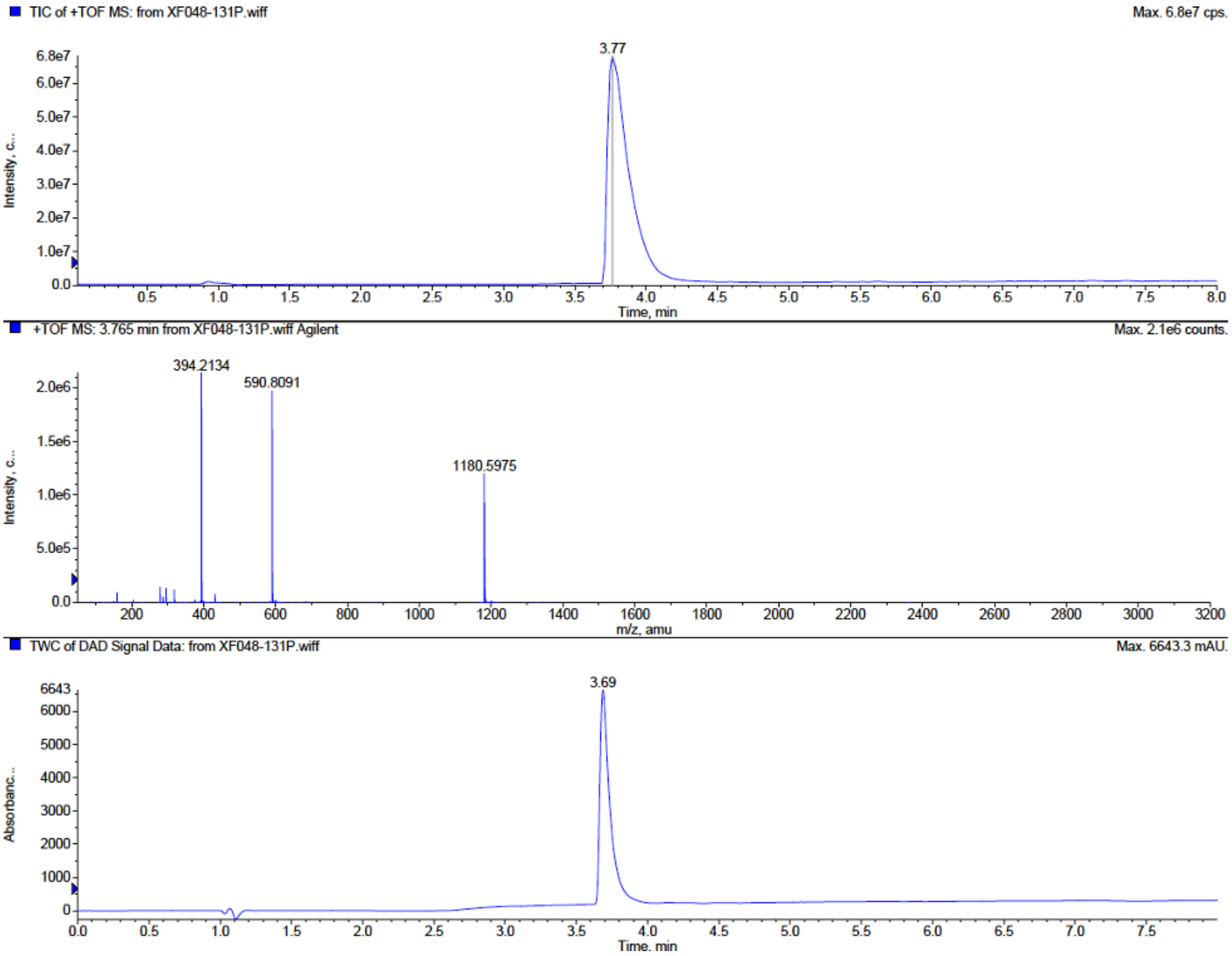

HPLC-HRMS spectrum of compound 7.

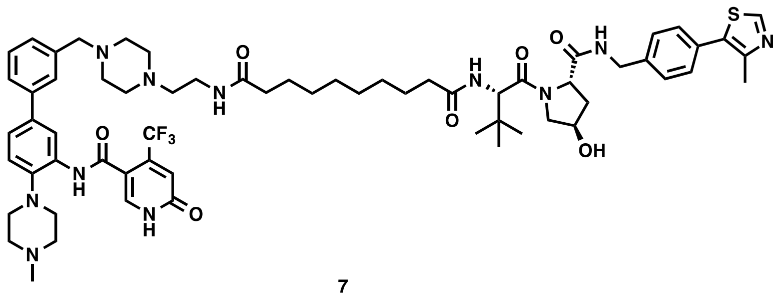

7

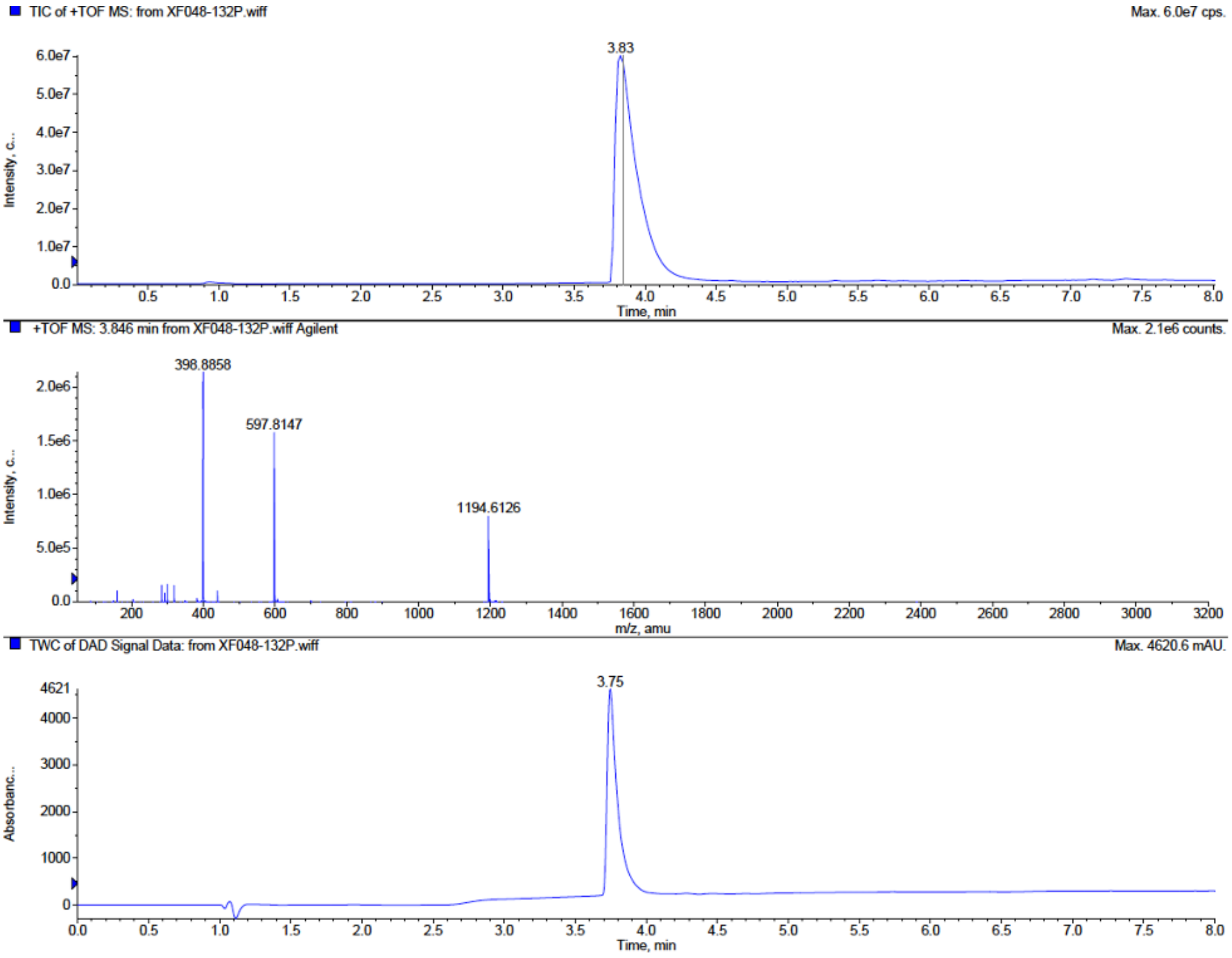

HPLC-HRMS spectrum of compound 8 (MS33).

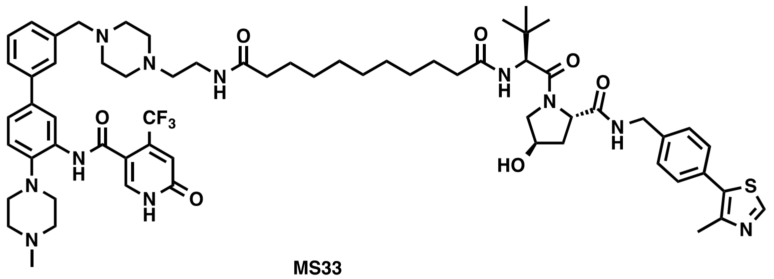

MS33

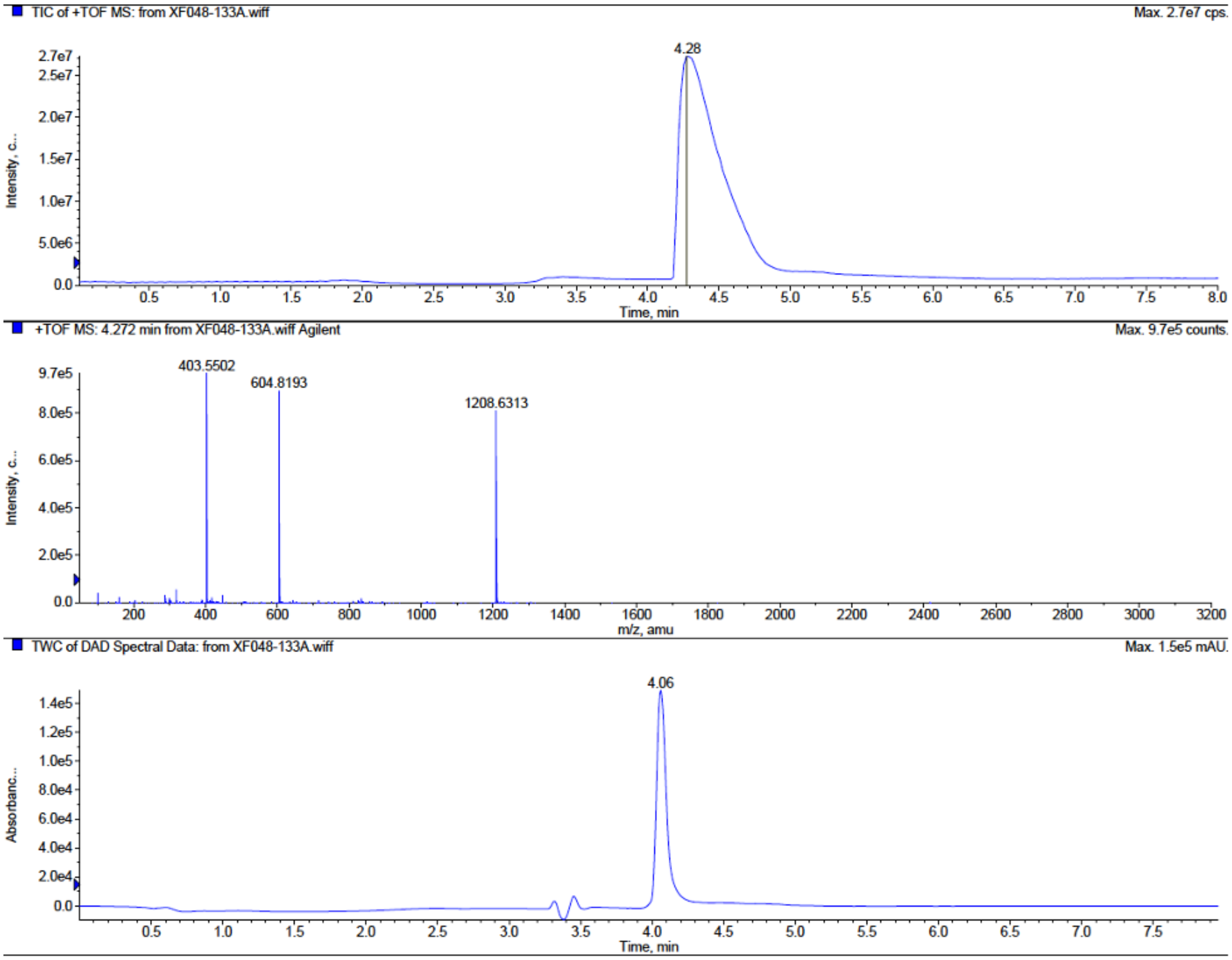

HPLC-HRMS spectrum of compound 9.

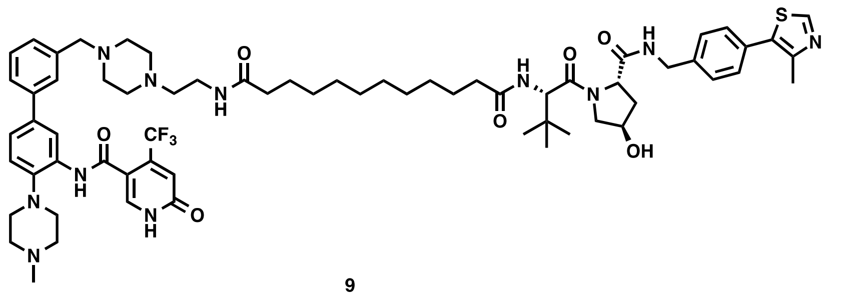

9

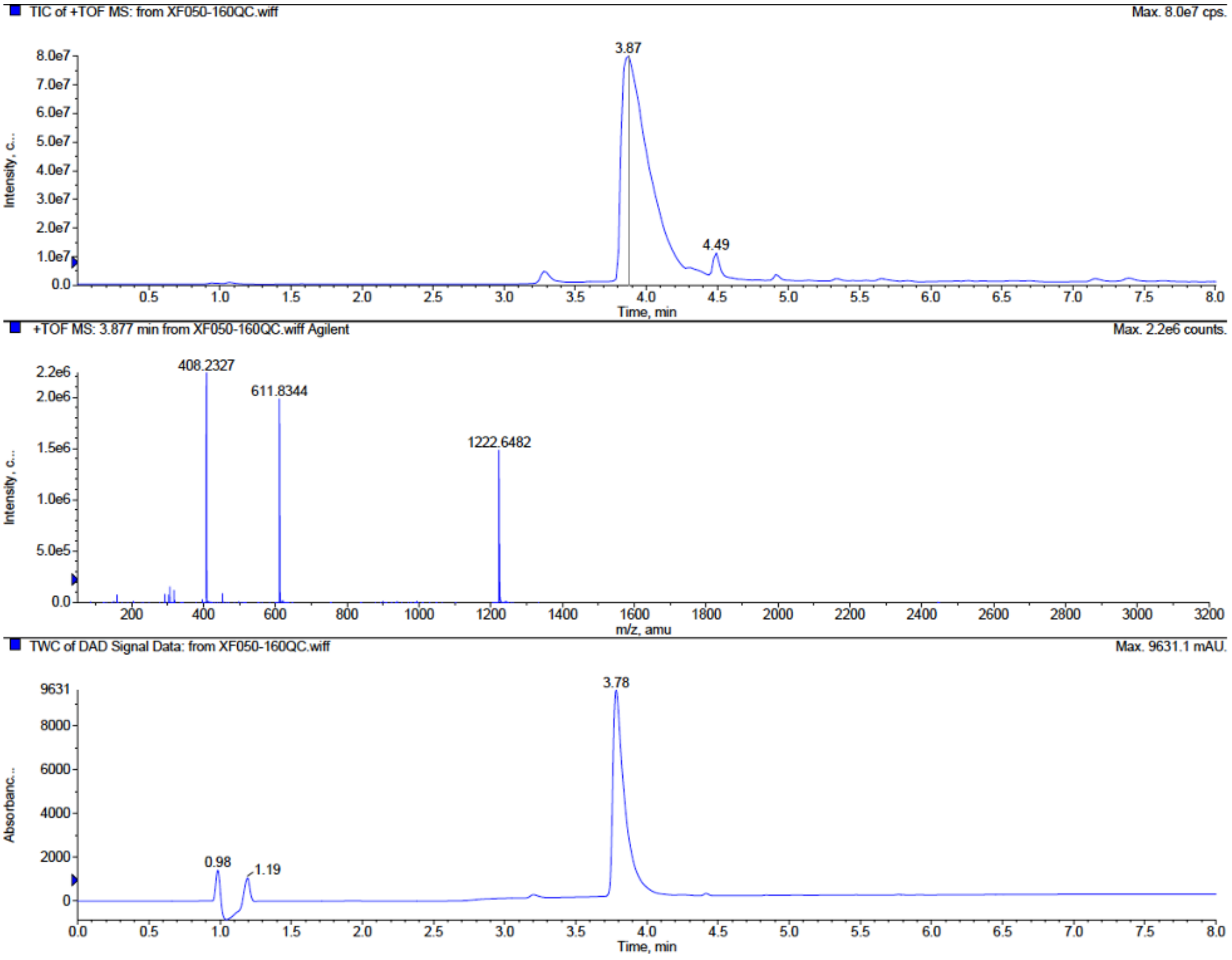

HPLC-HRMS spectrum of compound 10.

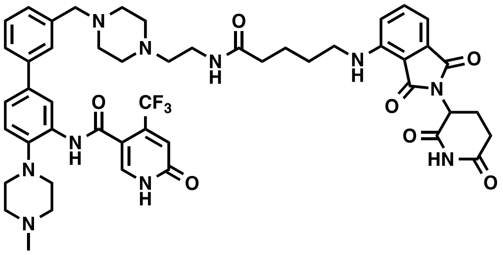

10

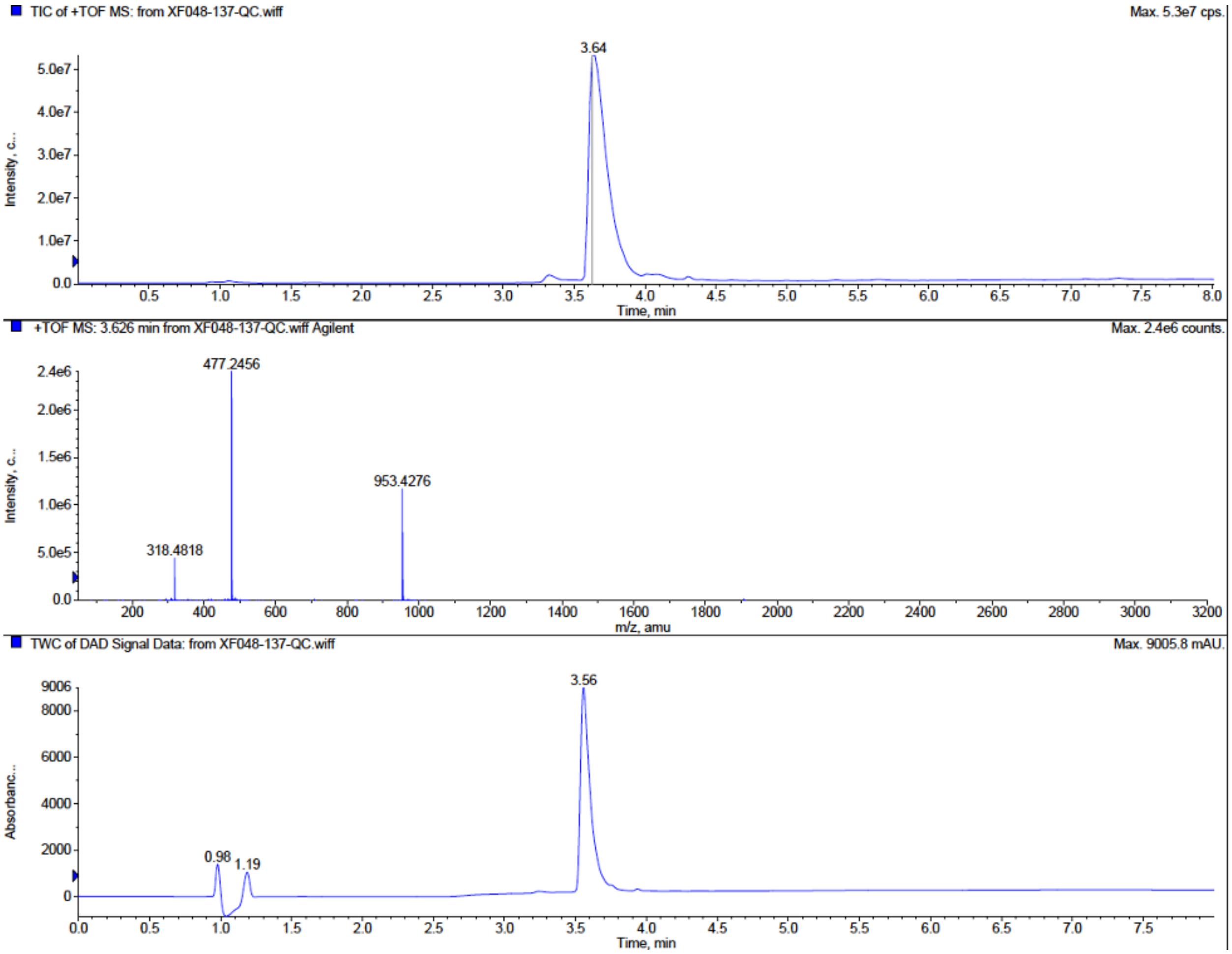

HPLC-HRMS spectrum of compound 11.

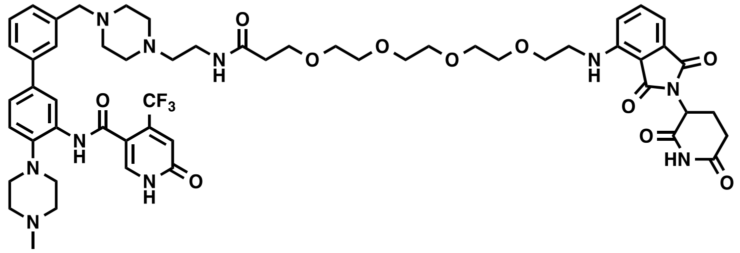

11

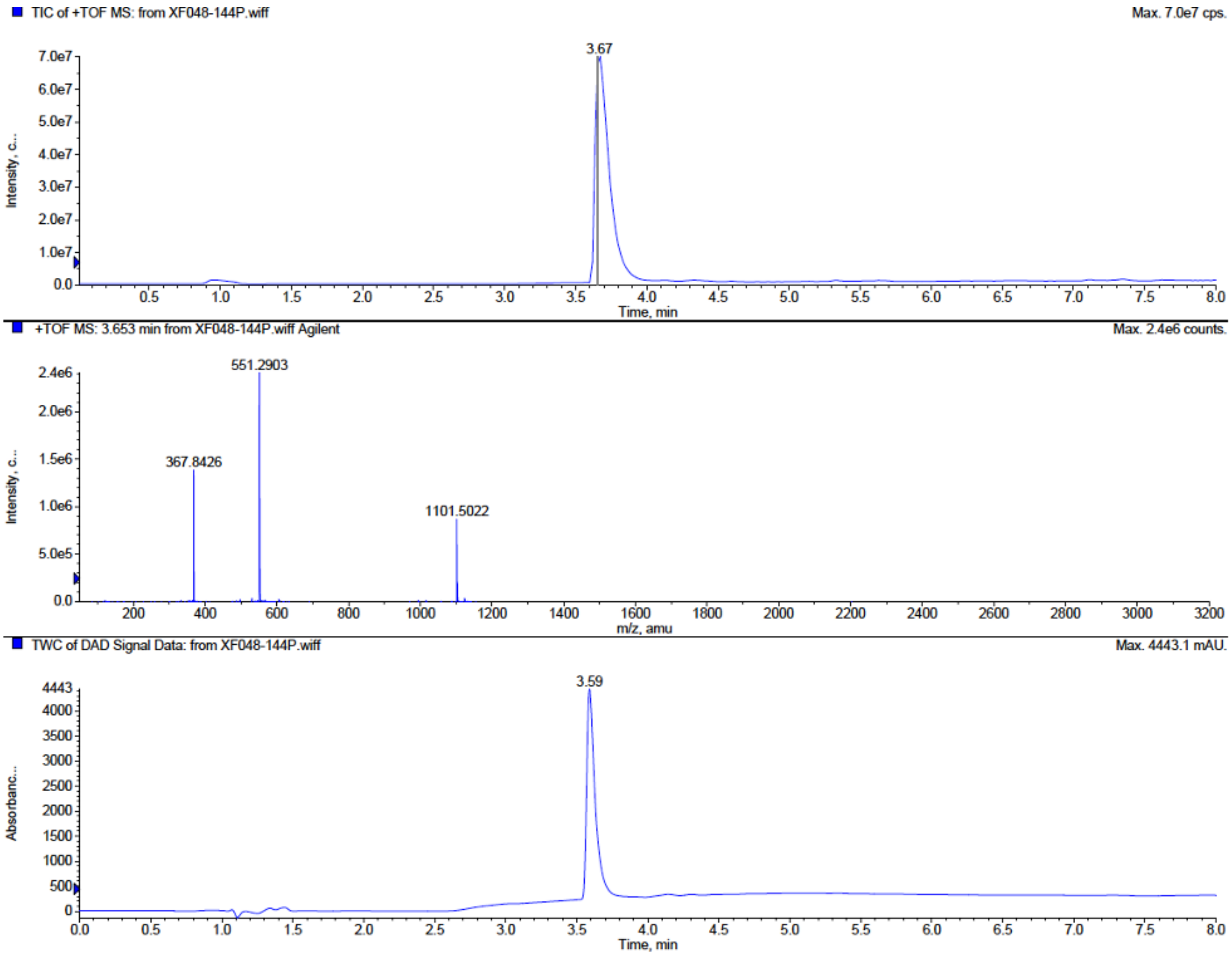

HPLC-HRMS spectrum of MS33N.

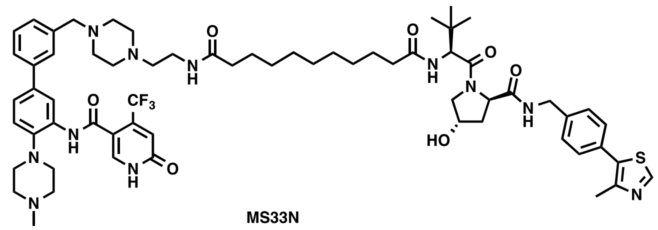

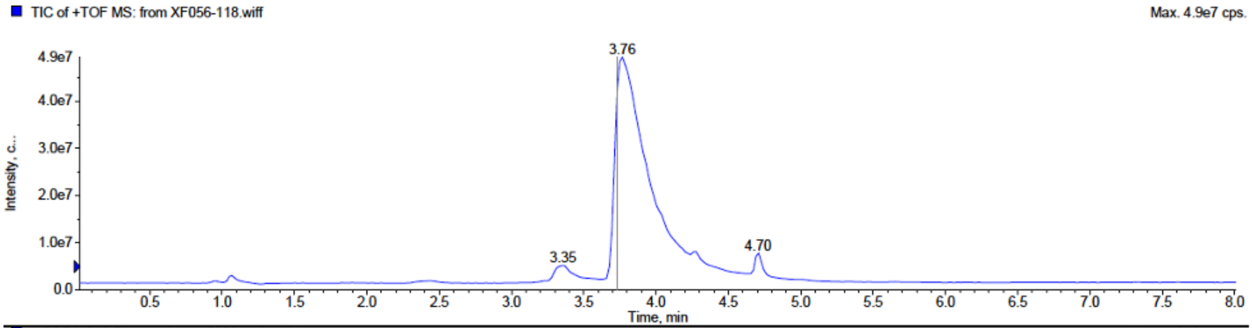

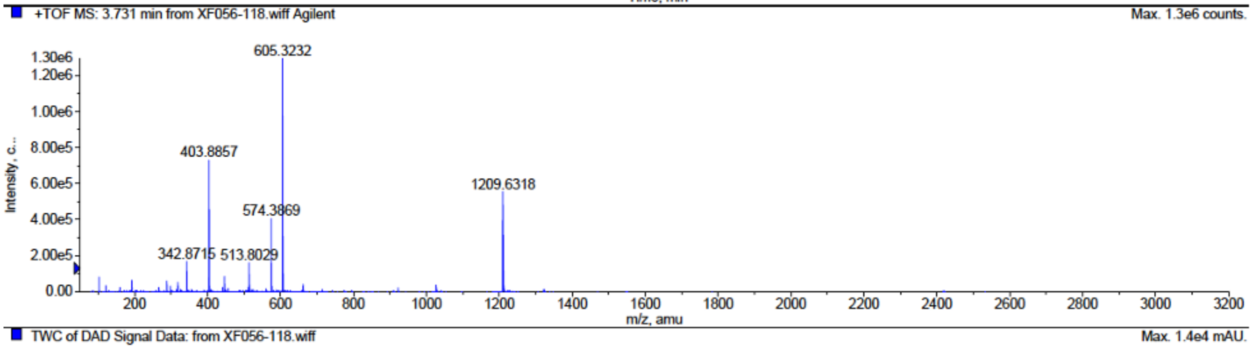

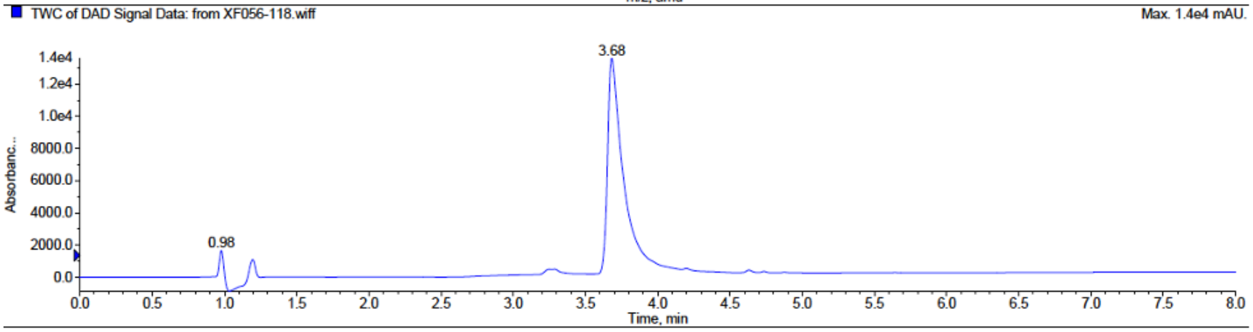

HPLC-HRMS spectrum of compound 12.

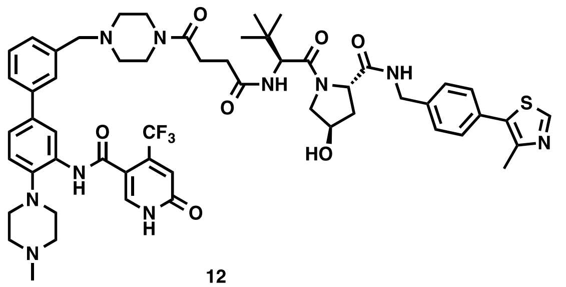

12

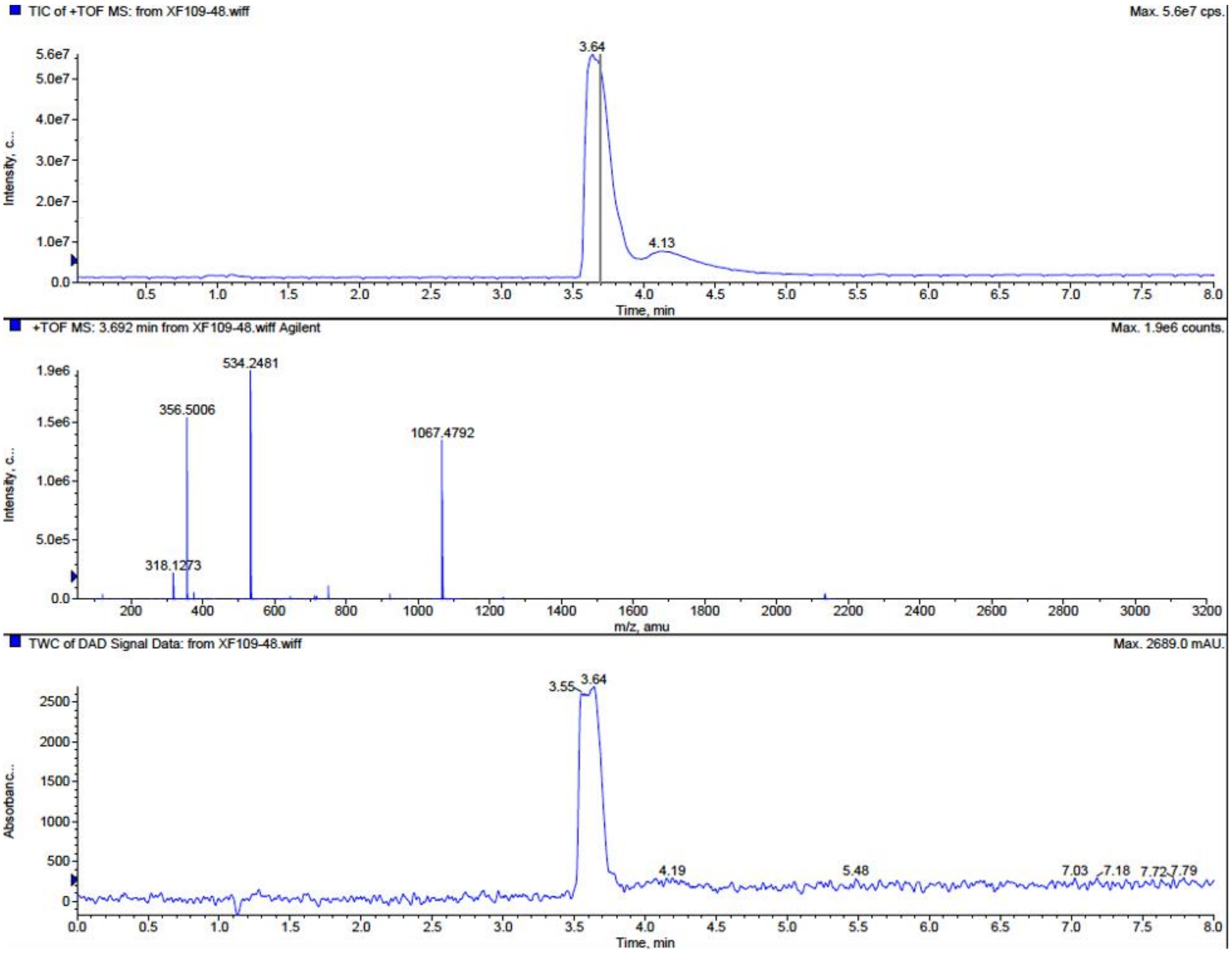

HPLC-HRMS spectrum of compound 13.

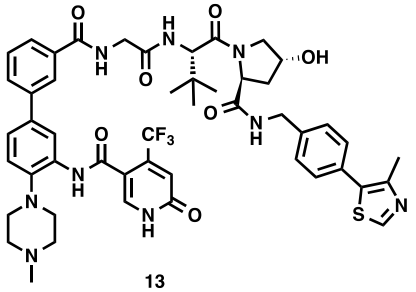

13

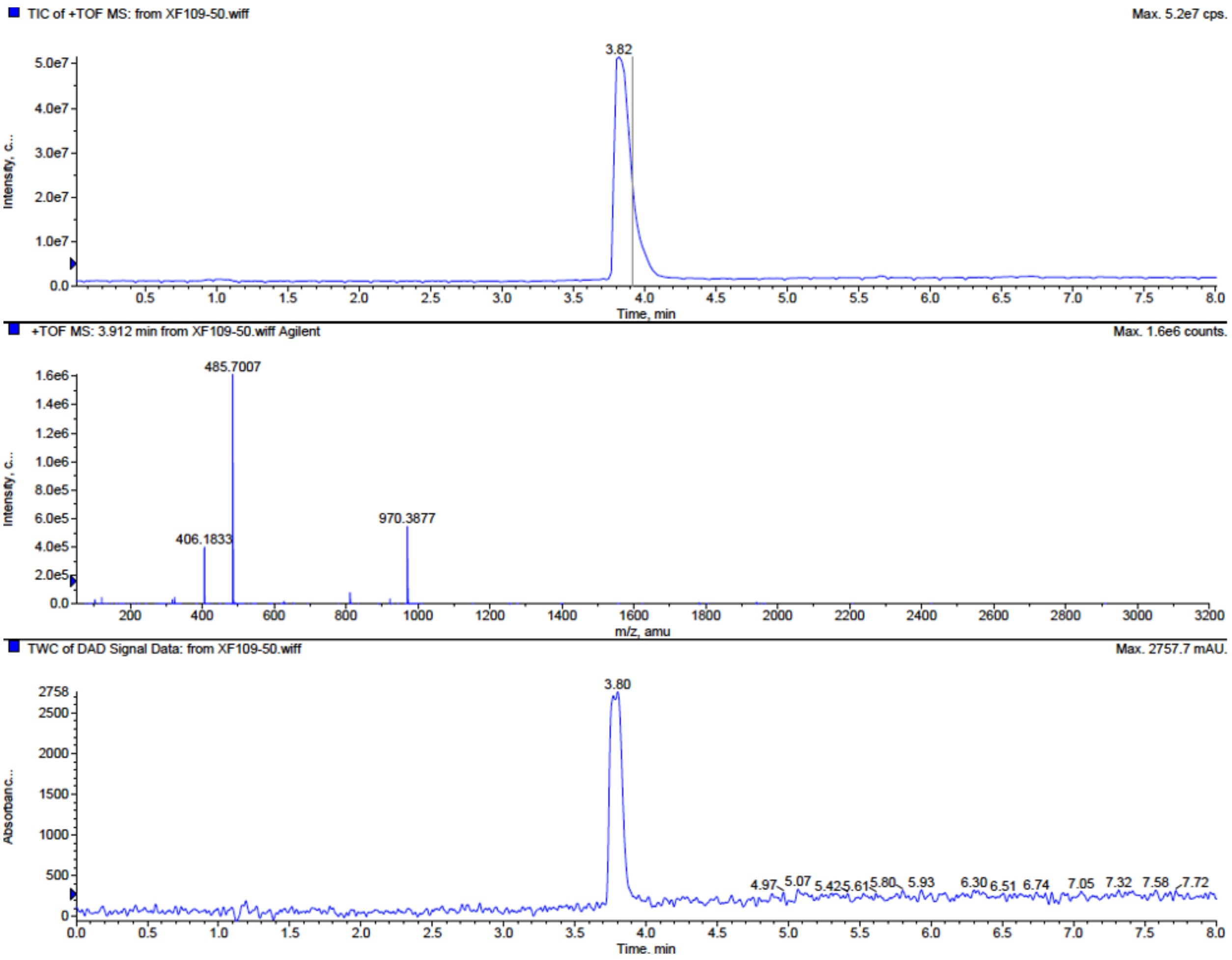

HPLC-HRMS spectrum of compound 14.

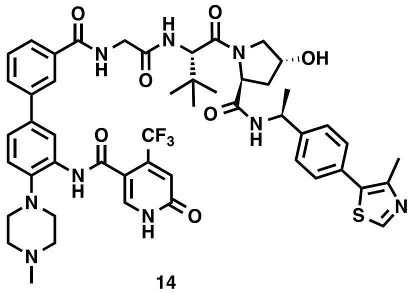

14

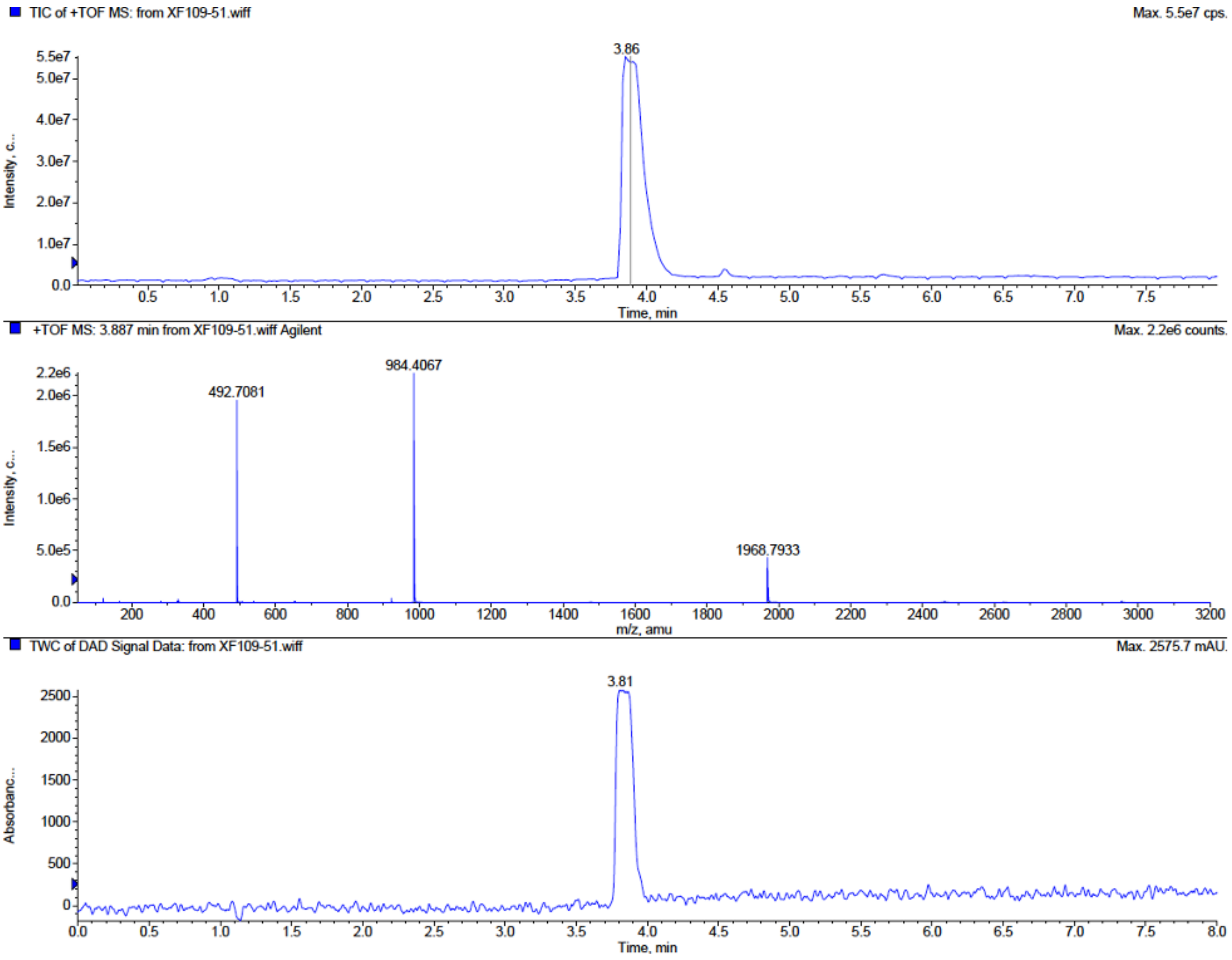

HPLC-HRMS spectrum of compound 15.

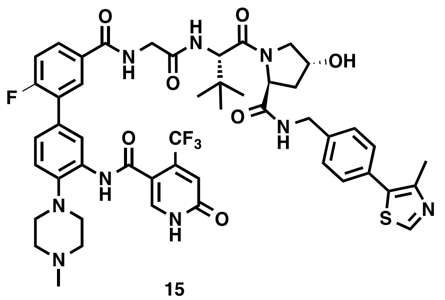

15

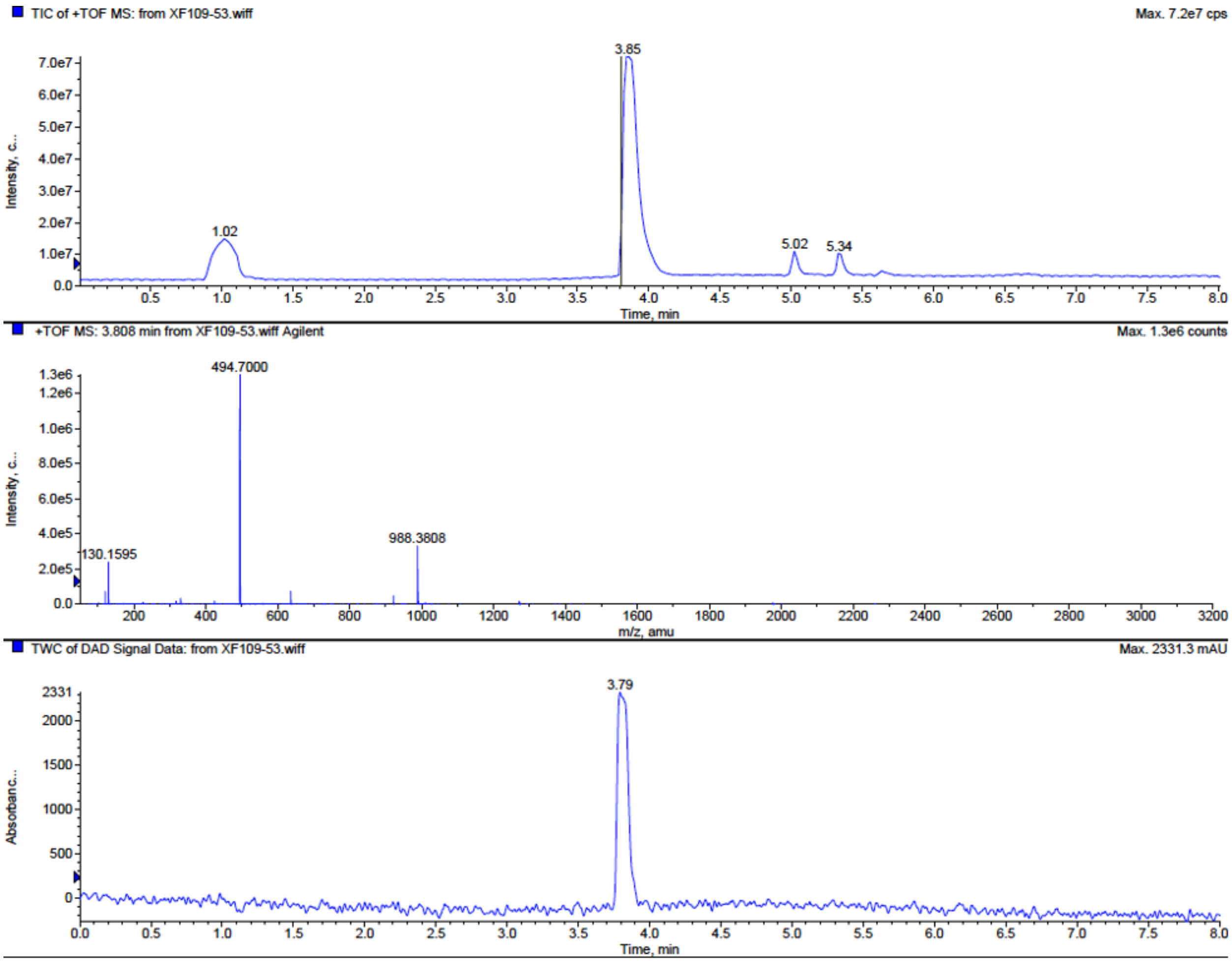

HPLC-HRMS spectrum of compound 16.

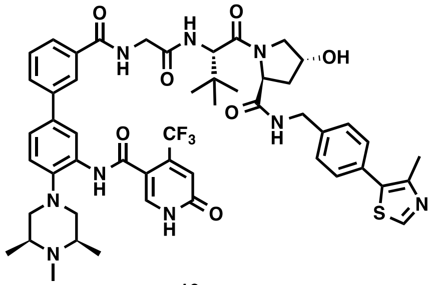

16

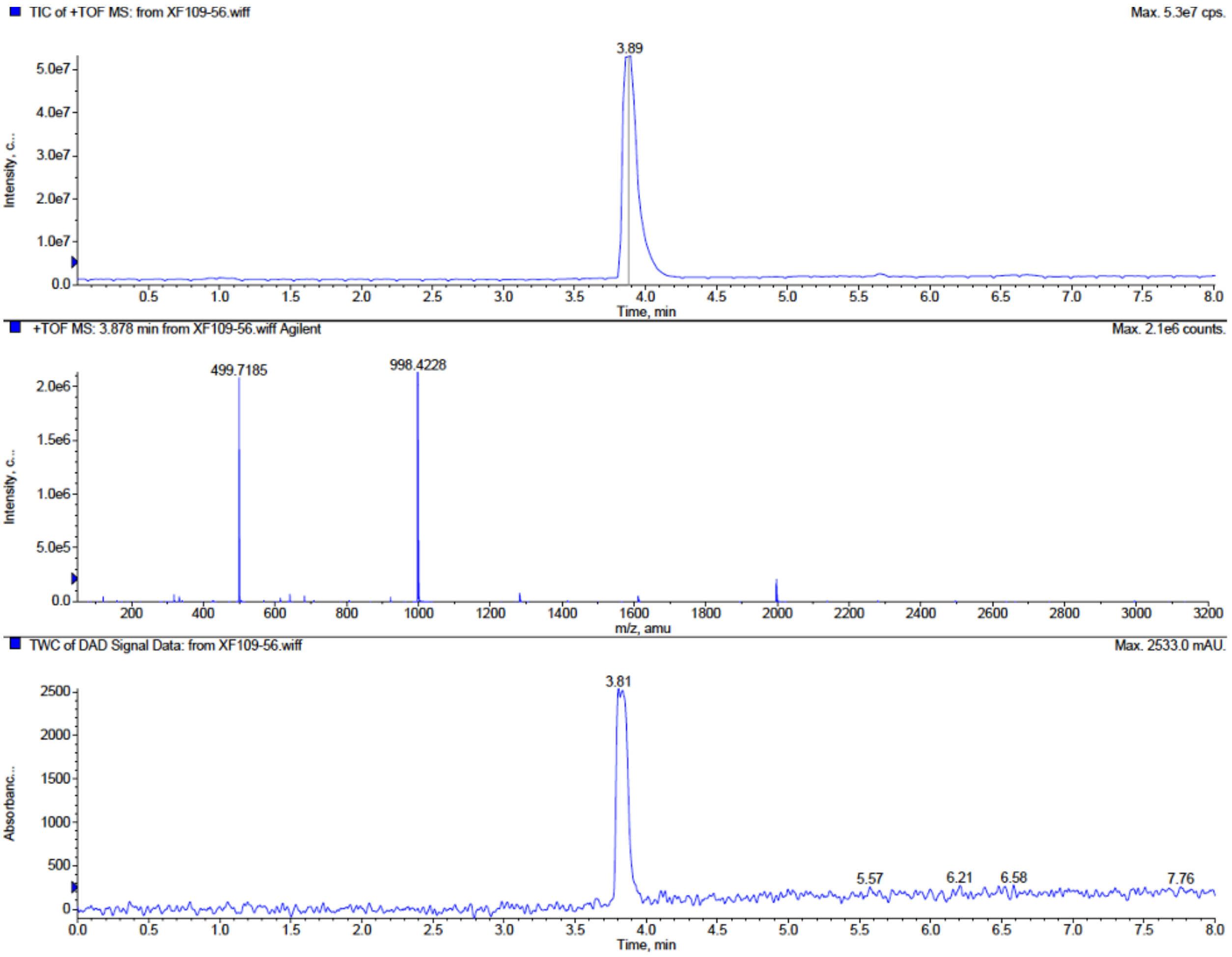

HPLC-HRMS spectrum of compound 17.

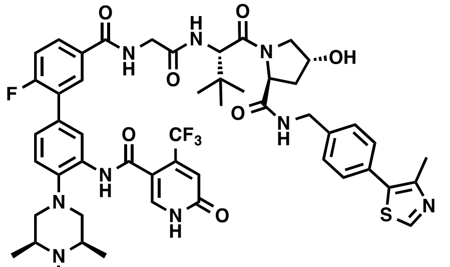

17

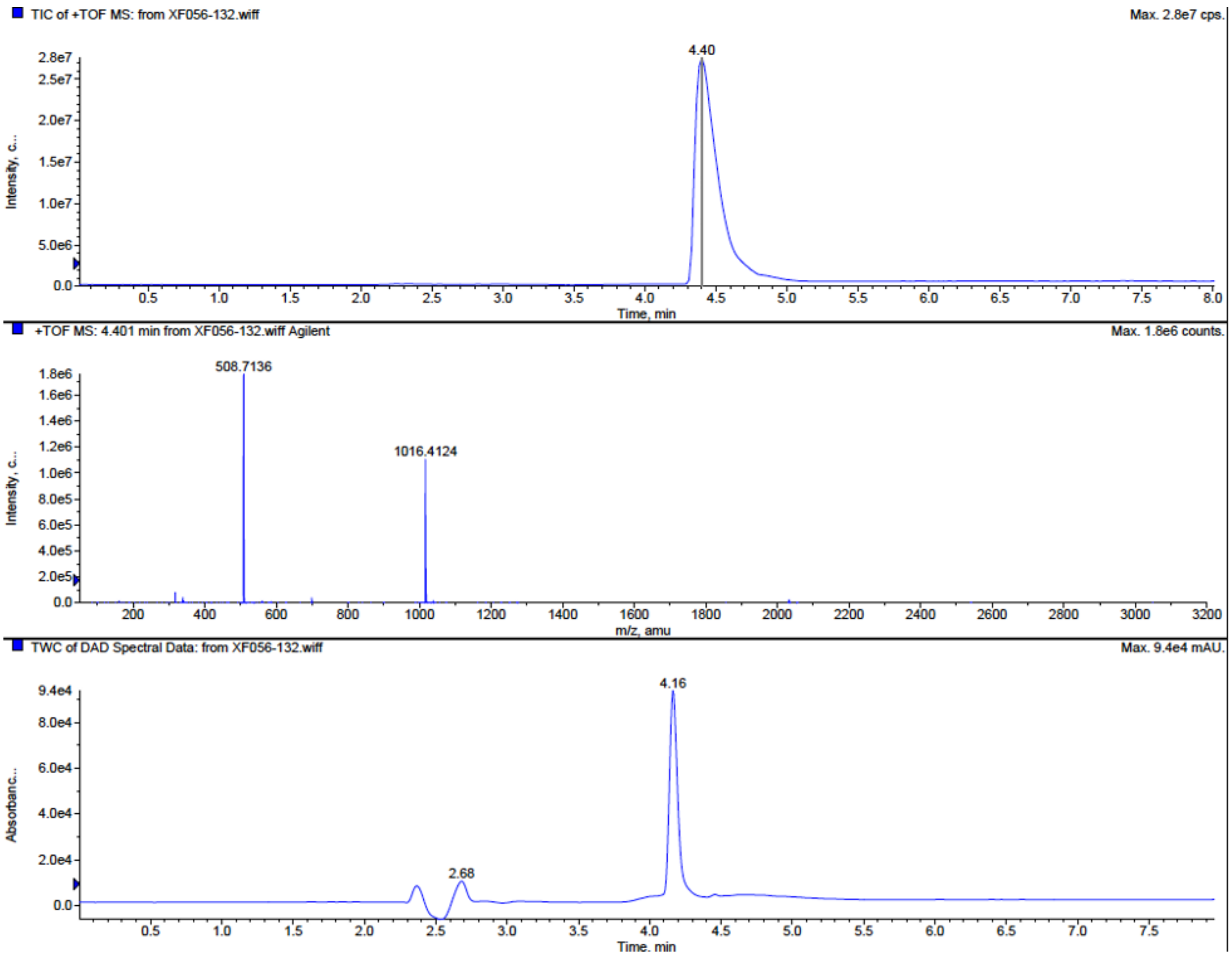

HPLC-HRMS spectrum of MS67.

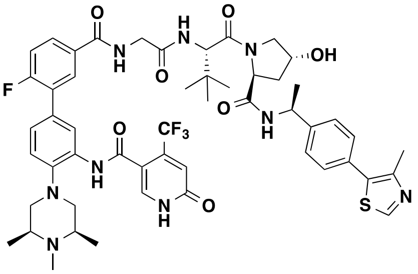
O
H
N
O
N
N
O
OH
F
O
N
H
O
CF3
O
N
H
N
H
O
N
N
S
N

MS67

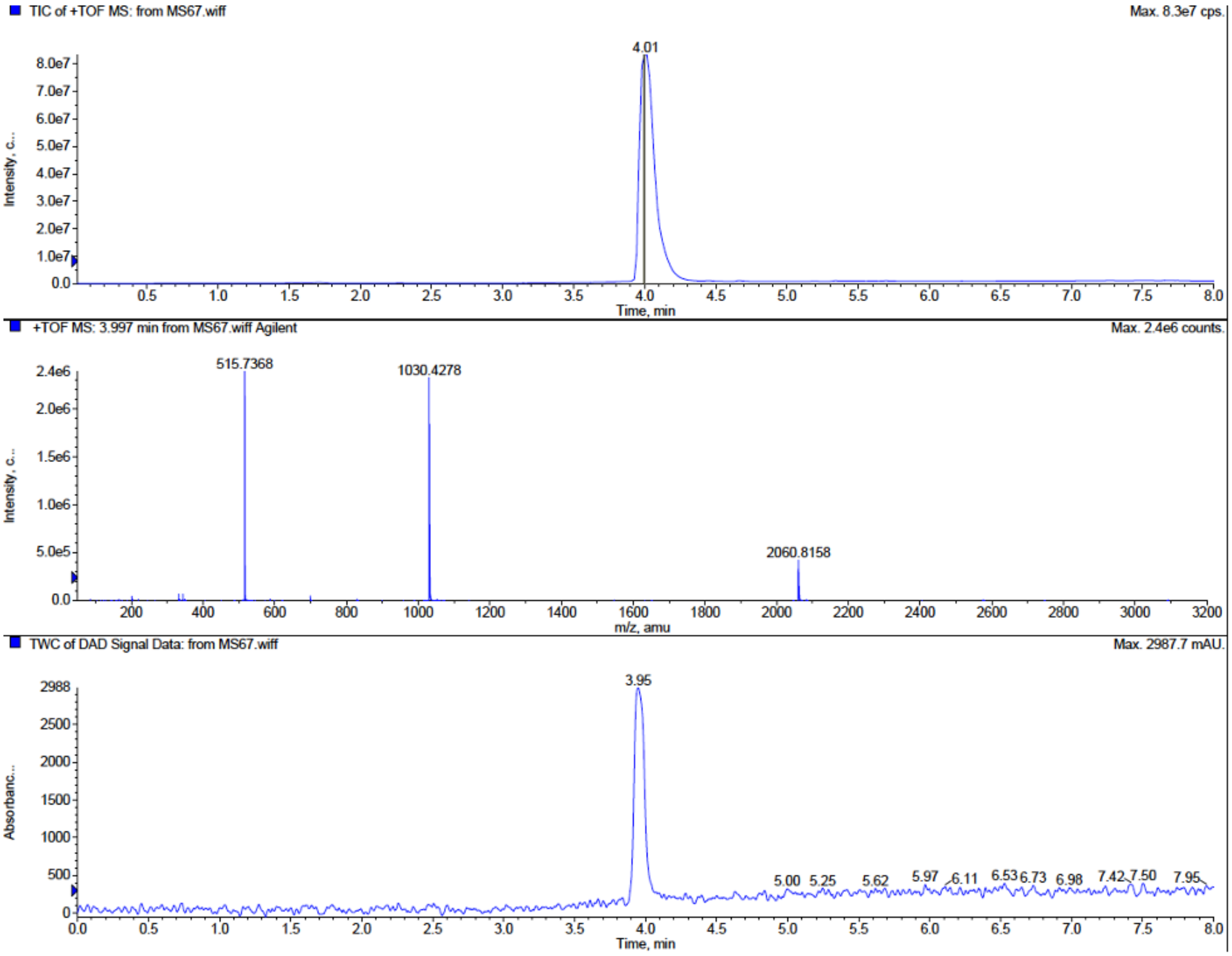
TIC of +TOF MS: from MS67.wiff
Max. 8.3e7 cps.
4.01
Intensity, c...
Time, min
+TOF MS: 3.997 min from MS67.wiff Agilent
Max. 2.4e6 counts.
515.7368
1030.4278
2060.8158
m/z, amu
TWC of DAD Signal Data: from MS67.wiff
Max. 2987.7 mAU.
3.95
Absorbanc...
5.00 5.25 5.62 5.97 6.11 6.53 6.73 6.98 7.42 7.50 7.95
Time, min

HPLC-HRMS spectrum of MS67N.

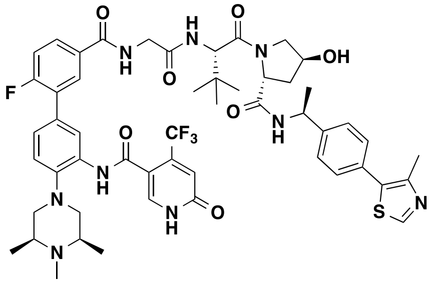

MS67N

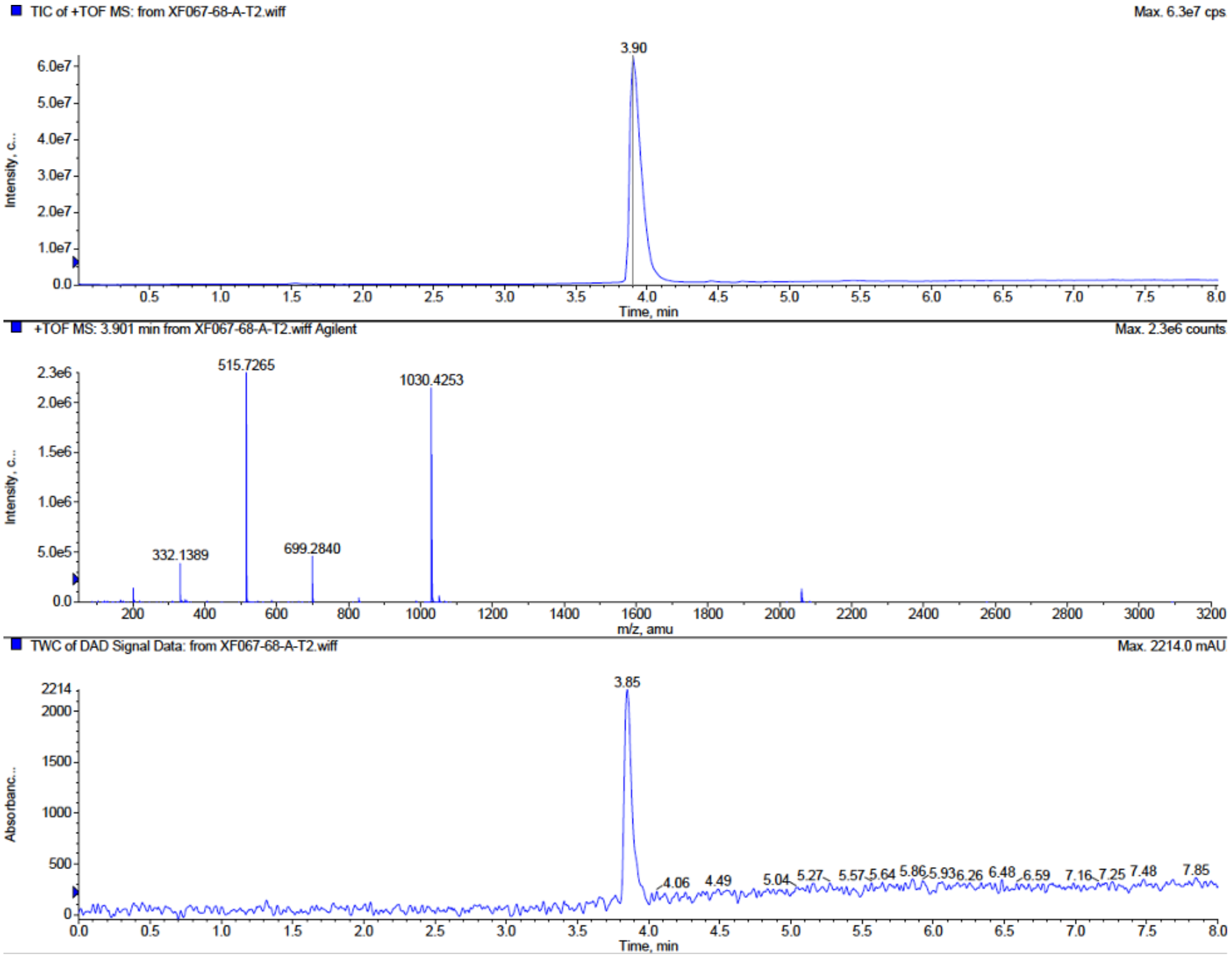

Cell lines and tissue culture

Human leukemia cells used, which include MV4;11 (American Tissue Culture Collection [ATCC], CRL-9591), RS4;11 (ATCC, CRL-1873), MOLM13 (Deutsche Sammlung von Mikroorganismen und Zellkulturen [DSMZ], ACC-554), KOPN8 (DSMZ, ACC-552), EOL-1 (DSMZ, ACC-386), K562 (ATCC, CCL-243) and HL60 (ATCC, CCL-240), were cultured in the RPMI 1640 base medium supplemented with 10% FBS and 1% Penicillin/Streptomycin. Human PDAC cells used in the work include BxPC-3 (ATCC, CRL-1687), HPAF-II (ATCC, CRL-1997) and Panc 10.05 (ATCC, CRL-2547) cells, which were cultured in RPMI 1640 supplemented with 10% FBS and 1% Penicillin/Streptomycin. The MIA PaCa-2 (ATCC, CRL-1420) PDAC cells and 293FT (Thermo Fisher Scientific, R70007) cells were cultured in DMEM supplemented with 10% FBS and 1% Penicillin/Streptomycin. Murine cell lines, transformed by human oncogene of MLL-AF9 (expressed from a biocistronic Ires-GFP vector), Flag-HA-tagged MLL-ENL (F-H-MLL-ENL) or co-expressed Hoxa9 plus Meis1 (Hoxa9/Meis1), were established as described previously (*49-51*). These cell lines were maintained in the Opti-MEM medium supplemented with 15% FBS (16000-044, Invitrogen), 1% Penicillin/Streptomycin, 50 μM of β-mercaptoethanol and supernatants from an mSCF-producer cell line (mSCF-CHO cells, gift of MP Kamps, UCSD).

Authentication of cell line identities, including those of parental and derived lines, was ensured by the Tissue Culture Facility affiliated to UNC Lineberger Comprehensive Cancer Center with the genetic signature profiling and fingerprinting analysis. Every 1-2 months, a routine examination of cell lines in culture for any possible mycoplasma contamination was performed using commercially available detection kits (Lonza).

CRISPR/Cas9-mediated VHL knockout (KO) in 293FT cells

The sgRNA sequence (5'-CGCGCGTCGTGCTGCCCGTA-3') targeting the human VHL gene was designed based on a CRISPR sgRNA database (GenScript; https://www.genscript.com/gRNA-database.html) and cloned into the pX458 vector (Addgene #48138) (*52*) as detailed in the F. Zhang Lab protocol (*53*, *54*). Then, the sgRNA-containing pX458 plasmid was transfected to 293FT cells using Lipofectamine 3000 (Thermo Fisher Scientific), followed by sorting of single GFP-positive 293FT cells two days post-transfection. Once the clonal 293FT lines were grown out, the VHL KO was examined by immunoblotting with anti-VHL antibody (Santa Cruz, sc-135657). The obtained clonal cells showing the best VHL-KO efficiency were used in the following experiments.

Preparation of proteins for ITC studies

For the expression of full-length N-terminal His-tagged WDR5 (Uniprot Accession Number P61964), pET28-LIC-WDR5 vector was obtained from Addgene (plasmid number 25489). The plasmid was transformed into *Escherichia coli* BL21 (DE3) cells and grown at 37°C until the culture reached an OD_{600} of ~ 2.0. The temperature was then reduced to 18°C and expression induced by the addition of 0.5 mM IPTG, followed by incubation for 16 h. The cells were then resuspended in a lysis buffer (50 mM Tris pH 7.5, 500 mM NaCl, 5% glycerol, 0.01% IGEPAL, 25 mM imidazole and 5 mM 2-mercaptoethanol) in the presence of Pierce Protease Inhibitor tablets, EDTA-free (Thermofisher), and 1 mM PMSF. The cells were lysed by sonication, clarified by centrifugation, the filtered supernatant loaded onto HisTrap HP affinity column (GE Healthcare), and the protein eluted using an imidazole gradient ranging from 25-250 mM. The fractions containing WDR5 protein were concentrated and subjected to size exclusion chromatography by HiLoad 26/600 Superdex 200 (GE Healthcare), preequilibrated with 20 mM HEPES pH 7.5, 150 mM NaCl and 2 mM TCEP.

For the expression of His-tagged VCB complex, a plasmid containing $pVHL_{54-213}$ (P40337) with an N-terminal His tag and a TEV protease cleavable site was co-transformed with a pCDF Duet plasmid containing $EloB_{1-104}$ (Q15370) and $EloC_{1-112}$ (Q15369) into *Escherichia coli* BL21 (DE3) cells. The cells were grown at 37°C until OD_{600} reached 2.0, and then the temperature was reduced to 18°C and IPTG added to a final concentration of 0.4 mM. The cells were harvested at 16 h post induction and resuspended in a lysis buffer (50 mM Tris pH 7.5, 500 mM NaCl, 5% glycerol, 0.01% IGEPAL, 25 mM imidazole and 5 mM 2-mercaptoethanol) in the presence of Pierce Protease Inhibitor Tablets, EDTA-free (Thermofisher) and 1 mM PMSF. The clarified lysate after centrifugation was loaded onto a 5 mL HisTrap HP affinity column and purified with an imidazole gradient. The protein was further purified by gel filtration using HiLoad 26/600 Superdex 200. The final purified protein was flash frozen in liquid nitrogen and stored at -80°C in 50 mM Tris pH 7.5, 150 mM NaCl and 2 mM TCEP.

Preparation of proteins for crystallization

For crystallization, an N-terminal 31-residues deleted construct of WDR5 (WDR5Δ31) was sub-cloned into pET28a-MHL vector with an N-terminal 6xHis tag. The protein was purified as described above and the 6xHis tag was cleaved using a TEV protease by dialyzing overnight in a buffer containing 50 mM Tris pH 7.5, 150 mM NaCl, 5% glycerol, 0.01% IGEPAL, 25 mM imidazole and 5 mM 2-mercaptoethanol at 4°C. To remove the cleaved 6xHis-tag, the protein was reapplied to the HisTrap column and the cleaved protein collected as flow through. The protein was further purified by size exclusion chromatography. In the case of VCB, the same plasmids and conditions were employed as described above. Additionally, the 6xHis tag was cleaved by TEV protease and the complex was further purified by size exclusion

chromatography using HiLoad 26/600 Superdex 200. Purified WDR5Δ31 and VCB were flash frozen and stored at -80°C in 50 mM Tris pH 7.5, 150 mM NaCl, and 1% glycerol.

Crystallization and structure determination of the VCB-MS33-WDR5Δ31 complex

VCB, MS33 and WDR5Δ31 were mixed in 1:1.5:1 ratio to form a stoichiometric ternary complex with a final concentration of 8 mg/mL, and screened for crystallization by sitting drop vapor diffusion, using various commercially available screen kits. Initial crystals were obtained at 20°C in a condition containing 0.1 M sodium-HEPES (pH 7.5-8.0), 1.3-1.6 M sodium thiocyanate, and 15-20% PEG 3350. Final diffraction quality crystals were grown by slowing the process of vapor diffusion by using a thin layer of paraffin oil: silicon oil mix (1:1) layered over the reservoir solution. The crystals were cryoprotected with reservoir solution containing 22% glycerol and flash cooled in liquid nitrogen. The X-ray diffraction data were collected at the NSLS-II 17-ID-2 beamline at the Brookhaven National Laboratory (BNL). The data were indexed and scaled using iMosflm and SCALA in the CCP4 suite (*55, 56*). The diffraction data were indexed in the space group $P2_1$ with unit cell parameters of a = 47.4 Å, b = 187.8 Å, c = 49.2 Å and α, γ = 90˚, β = 116.96˚ with one copy of the ternary complex in the asymmetric unit.

The structure was solved by molecular replacement using PHASER MR (*57*) with the search models derived from the coordinates of VCB (PDB: 1VCB (*58*)) and WDR5 (PDB: 2GNQ (*59*)). Subsequent iterative manual building and refinement were performed with programs Coot and PHENIX respectively (*60, 61*). The structure was refined to R_{work} and R_{free} values of 20.0% and 22.6%, respectively. The structure shows good stereochemistry with 96.8% of the residues in the favorable regions and only 3.2% of residues in the allowed regions of the Ramachandran plot. Data collection and refinement statistics of the VCB-MS33-WDR5 complex structure are included in Table S1.

Crystallization and structure determination of the VCB-MS67-WDR5Δ31 complex

VCB, MS67 and WDR5Δ31 were mixed in 1:1.5:1 ratio to form a stoichiometric ternary complex at a final concentration of 10 mg/mL. Diffraction quality crystals were observed from solutions containing 0.1 M Tris pH 8.5, and 30% PEG 300. The crystals were directly flash cooled in liquid nitrogen and X-ray data collected at the NSLS-II 17-ID-2 beamline at the BNL. The data were processed using iMosflm and SCALA in CCP4 suite (*55, 56, 62*). The diffraction data were indexed in the space group $P2_12_12_1$ with unit cell parameters of a = 63.8 Å, b = 98.6 Å, c = 127.6 Å and α, β, γ = 90˚ with one copy of the ternary complex in the asymmetric unit. The structure was solved by molecular replacement as described above. The models were improved by iterative manual building and refinement with programs Coot and PHENIX respectively (*60, 61*). The structure was refined to R_{work} and R_{free} values of 19.1% and 22.4%, respectively, with 96% of the residues in Ramachandran favored regions and none in the disallowed regions.

Coordinate files for degraders were generated using Chembio Draw software suite or PRODRG server (*63*). Ligand restraint files were generated using eLBOW (*64*) from the PHENIX suite. All molecular graphic figures were prepared by PyMOL (Schrödinger LLC). Data collection and refinement statistics of the VCB-MS67-WDR5 complex structure are included in Table S1.

Isothermal titration calorimetry (ITC)

Both VCB and WDR5 proteins were dialyzed overnight in a buffer containing 20 mM Bis-Tris Propane pH 7.4, 150 mM NaCl, 1 mM TCEP, 0.0075% Tween20. Titrations were performed on a Microcal ITC200 instrument in the reverse mode at 25 °C as described previously (*39*). Briefly, WDR5 (150 μM or 200 μM in syringe) was titrated into 15 μM of MS33 or 20 μM of MS67 in the cell. At the end of the titration, the excess of the solution was removed and the syringe was

washed and dried. For the ternary titrations, the VCB complex (126 μM or 168 μM) was loaded in the syringe and titrated into the degrader-WDR5 complex (12.6 μM or 16.8 μM) in the cell. For the VCB-degrader binary titrations, an initial single injection of buffer was added into 15 μM of MS33 or 20 μM of MS67 in the cell followed by titration of VCB complex (126 μM or 168 μM in the syringe). Titrations for negative controls (MS33N and MS67N) were performed in reverse mode on a MicroCal PEAQ-ITC Automated (Malvern Panalytical) instrument. The data were fitted to single binding site model using the Origin 7.0 software, supplied by MicroCal. Cooperativity values (α) were determined by calculation the ratio of the K_d obtained from the binary VCB to degrader titration and K_d of the ternary VCB into degrader:WDR5 complex titration. The reported values represent the mean ± SD from three independent measurements.

Selectivity assays

Selectivity assays against 22 human protein methyltransferases were performed by Reaction Biology Corp using miniaturized radioisotope-based filter binding assay (HotSpot). This biochemical assay monitors the transfer of a ^{3}H-labeled methyl group from the cofactor SAM to the substrate. 22 Methyltransferases (DOT1L, EZH1 complex, EZH2 complex, G9a, GLP, NSD1, NSD2, NSD3, PRMT1, PRMT3, PRMT4, PRMT5/MEP50 complex, PRMT6, PRMT7, PRMT8, SET1b complex, SET7/9, SET8, SETD2, SMYD3, SUV39H1 and SUV39H2) were used as the enzymes. The concentration of the substrate core histone proteins was 5 μM, or 0.05 mg/mL of nucleosomes from HeLa/Chicken or core histones from Chicken. The concentration of the cofactor SAM was 1 μM. MS67 was tested at 10 μM in duplicate.

Selectivity assays against a panel of human kinases were carried out by Eurofins Cerep with the MS67 concentration tested at 1 μM in duplicate. The compound enzyme inhibition activity was calculated as percent of inhibition of control enzyme activity.

Selectivity screening of MS67 against 44 GPCRs, ion channels and transporters was performed by NIMH-PDSP (http://pdsp.med.unc.edu/) in radioligand binding assays. MS67 was tested at 1 μM in quadruplicate. The K_i value of MS67 binding to Sigma 2 receptor was calculated using Sigma 2 ligand haloperidol as control in triplicate.

Inducible WDR5 knockdown (KD)

Two human WDR5-targeting shRNAs (see Data File S1 for sequence information) were ordered and cloned into a doxycycline-inducible Tet-pLKO-puro vector (Addgene #21915) (*65*). Viral particles were produced with Tet-pLKO-puro-shWDR5 and the packaging plasmids, psPAX2 (Addgene #12260) and pMD2.G (Addgene #12259), in 293FT cells. After the virus infection was performed in target cells, the infected cells were selected with 1 μg/mL of puromycin for 7 days.

Antibodies and immunoblotting

Total cell lysate was used for western blots as previously described (*66*). The following primary antibodies were used in the study: WDR5 (Santa Cruz, sc-393080), c-MYC (Cell Signaling Technology, 3873), Menin (Santa Cruz, sc-374371), RBBP5 (Santa cruz, sc-390954), MLL-C (*67*) (a kind gift of Y. Dou), H3 (Abcam, ab1791), H3K4me1 (Abcam, ab8895), H3K4me2 (Millipore, 07-030), H3K4me3 (Active Motif, 39159), H3K9me3 (Abcam, ab8898), H3K27me3 (Millipore, 07-449), H3K36me3 (Abcam, ab9050), Tubulin (Cell Signaling Technology, 2144), and Vinculin (E1E9V)XP (Cell Signaling Technology, 13901S), and Flag M2 antibody (Sigma, F1804). Blots were imaged using fluorescence-labeled secondary antibodies on ChemiDoc™ Imaging Systems.

Mutagenesis on human WDR5 and generation of stable cell lines with WDR5 mutants.

The WDR5 wild type (WT) and mutant nucleotide sequences were modified from p3x Flag-CMV-14-WDR5 (Addgene plasmid #59974). Point mutations were introduced by PCR using Q5 Site-Directed Mutagenesis kit (New England E0554S). The mutagenic primers used are shown in Data File S1. PCRs for single amino acid mutations were run for 25 cycles of 30 s at 98 °C and 1 min at 68 to 74 °C, 2 mins 30 s at 72 °C, followed by 10 mins at 72 °C. The resulting mutant plasmids were verified by Sanger sequencing. The WDR5 WT and mutant PCR fragments with 3X flag-tag were cloned into lentiviral TetO-Puro vector (engineered by Dr. Dung Fang Lee, University of Texas, Health Science Center at Huston). The lentiviral TetO-WDR5-Puro vectors were utilized to transduce HEK293T cells to generate inducible/stable cell lines that overexpress WDR5 WT and mutants. For lentivirus production, HEK293T cells were seeded in DMEM medium supplemented with 10% fetal bovine serum. Plasmid DNA vectors containing human WDR5 WT and its mutants were transfected using Lipofectamine (3000) according to the manufacturer's protocol. After 48 and 72 h transfection, lentivirus packaging cell supernatant were collected and concentrated using Lenti-X concentrator (Takara, 631232). Concentrated lentivirus was quantified using Lenti-GoStix Plus (Takara 631280) and applied to HEK293 cells for 24 h in the presence of 5 μg/mL polybrene (Sigma). WDR5 overexpression was induced by the addition of 1 μg/mL doxycycline to the medium. After puromycin selection for four days, the stable cell lines were utilized for further analytic experiments. Cell lysates were collected at 72 h after the stable cell lines were treated with indicated concentrations of MS67. Supernatants were run on 4-12% gradient SDS-PAGE gels and transferred to nitrocellulose membrane (BioRad) using the Trans-Blot Turbo system (BioRad). Membranes were probed with WDR5 (SantaCruz, G-9) and Vinculin (Cell Signaling, 13901S) antibodies. Western blot images were detected using

LI-COR machine (LI-COR Biosciences). The Images were cropped at specific protein band of interest using Image Studio (LI-COR Biosciences) to improve the clarity of data presentation.

Washout experiments

MV4;11 or MIA PaCa-2 cells were treated with 0.5 μM MS67 for 12 h. The MS67-containing medium were removed and cells were washed twice with 1x PBS. Cells were then re-cultured in normal medium and collected at indicated time points.

Cell extract fractionation

For analysis of chromatin-bound fractions, cells were first washed twice with cold PBS and then pelleted at 300 rpm for 5 min at 4 °C. The cell pellet was resuspended in CSK buffer (10 mM PIPES pH 7.0, 100 mM NaCl, 300 mM sucrose, 3 mM $MgCl_2$, 0.5% Triton X-100) supplemented with TSA and protease and phosphatase inhibitors, and incubated on ice for 10 min followed by centrifugation at 1500 g for 10 min. After removing the supernatant, the remaining cell pellet was used as the chromatin-bound fraction and ready to be resuspended in the protein sample buffer for western blot analysis.

Cell proliferation assay by counting

1 - 5 x 10^5 /mL of cells were seeded in triplicate in the 24-well plates. Compounds were added at a range of concentrations indicated in the study. Medium with fresh compounds were changed every two days. Cells were passaged with dilution to keep cell density under 1 x 10^6 /mL at all time. Cells were counted by an automated cell counter (Biorad, TC10) every two days. 50% of maximal growth inhibition (GI_{50}) values were calculated using GraphPad Prism software using a nonlinear regression analysis and the mean ± SEM calculated from triplicated treatment data.

MTS (3-(4,5-dimethylthiazol-2-yl)-5-(3-carboxymethoxyphenyl)-2-(4-sulfophenyl)-2H-tetrazolium) assay

Pancreatic cancer cells were seeded at a density of 500 or 1000 cells per well in 96-well plates and incubated with indicated compounds at different time-points. Fresh medium with compound was changed every two days. At each time-point, MTS reagent (Promega) was added to the cell culture medium and incubated for 0.5 - 2 h under standard culture conditions. Then, the plates were briefly shaken and subjected to measure absorbance at 490 nm using CYTATION-5 imaging reader (BioTek).

Cell cycle progression and cell apoptosis assay

Cells were collected, washed twice with cold PBS, and fixed in 80% methanol by incubation for at least 3 hours at -20 °C. Cells were then stained with 20 μg/mL of propidium iodide (PI, Sigma) plus 100 μg/mL of DNase-free RNase A (Roche) for 30 mins at room temperature. DNA content was detected using a Thermo Fisher Attune NxT flow cytometer, followed by the analysis with ModFit Software (VeritySoftware House). For apoptosis assay, cells were first washed with cold PBS and then stained with Annexin and PI using the apoptosis kit (BD Biosciences) following the manufacturer's manuals. Apoptosis was detected using a Thermo Fisher Attune NxT flow cytometer and then analyzed by FlowJo Software (BD). All flow cytometric analyses were performed at the Flow Cytometry core in the University of North Carolina at Chapel Hill.

Cell proliferation assay for evaluating MS67 in MCF-7, NCI-H2009, PC3 and SK-ES-1cells.

Cell proliferation assays were performed for MS67 in MCF-7, NCI-H2009, PC3 and SK-ES-1 cells. Compound treatment of cells started one day after seeding with a final DMSO concentration of 0.1% and was performed by nanodrop-dispending using a Tecan Dispenser. 0.1% DMSO (solvent) and Staurosporine (1.0E-05M) served as high control (100% viability) and low control (0% viability), respectively. MS67 was tested at 8 concentrations with 30 μM as the top concentration and 9 nM as the low concentration (three folds dilution).

For the assay, cells were seeded in white cell culture-treated flat and clear bottom multi-well plates and incubated at 37 °C overnight before MS67 was added. After incubation for 72 h at 37 °C at 5% or 10% CO_2 dependent on the medium, cell plates were equilibrated to room temperature for 1 h, CellTiterGlo reagent (Promega) was added and luminescence was measured approximately 1 h later using a luminometer. Raw data were converted into percent cell viability relative to the high and low control, which were set to 100% and 0%, respectively. Each assay was performed in duplicate.

Chromatin immunoprecipitation followed by deep sequencing (ChIP-seq)

ChIP-seq was performed as previously described (*68*) with slight modifications. A fraction of the Drosophila chromatin was used as spike-in control according to published procedures (*69*). For ChIP-seq, the culture medium was removed from the cells, and crosslinking was performed in 1% of formaldehyde (diluted in PBS; added freshly with protease inhibitor PMSF) for 10 minutes with shaking at room temperature. Then, crosslinking was quenched by 125 mM of glycine as final concentration for 10 mins, followed by three times' washes with cold PBS and harvesting. Cells were lysed with the Lysis Buffer (10 mM Tris-HCl, pH 8.0, 10 mM NaCl, 0.2% CA630 plus protease inhibitor cocktail and PMSF) and nuclei were washed once with the Nuclei Wash Buffer (10 mM Tris-HCl, pH 8.0, 200 mM NaCl, 1 mM EDTA and protease inhibitor cocktail). Nuclei were resuspended in the Nuclei Lysis Buffer (50 mM Tris-HCl, pH 7.5, 5 mM EDTA, 0.5% SDS, plus the protease inhibitor cocktail and PMSF), followed by sonication using the Bioruptor 300 machine (Diagenode). SDS in the sonicated chromatin sample was diluted to a final concentration of 0.1% by adding the ChIP Dilution Buffer (50 mM Tris-HCl, pH 7.5, 1 mM EDTA, 150 mM NaCl, 1% TritonX-100). For spike-in control (*69*), same amount of sonicated chromatin from each experimental condition was mixed with 300 ng of the already sonicated

chromatin from the crosslinked Drosophila S2 cells before performing immunoprecipitation. Preclearing of mixed chromatin was done by adding 20 µL of Protein A/G magnetic beads (Thermo Scientific) slurry and incubating at 4 °C for 1 h. Pre-cleared chromatin was centrifuged and supernatant was incubated with both H3K4me2 (Millipore, 07-030) antibody and H2Av (Drosophila-specific; Active Motif, 39715) antibody overnight at 4 °C. Then 50 µL of Protein A/G magnetic beads (Thermo Scientific) were added to capture the specific chromatin complexes and incubated at 4 °C for 2 h. Beads were washed subsequently with the following buffers: one time with Low Salt Buffer (20 mM Tris-HCl pH 8.0, 150 mM NaCl, 2 mM EDTA, 1% Triton X-100, 0.1% SDS); one time with High Salt Buffer (20 mM Tris-HCl pH 8.0, 500 mM NaCl, 2 mM EDTA, 1% Triton X-100, 0.1% SDS), one time with LiCl Buffer (10 mM Tris-HCl pH 8.0, 1 mM EDTA, 500 mM LiCl, 1% CA630, 1% sodium deoxycholate) and two times with TE Buffer (10 mM Tris-HCl pH 8.0, 1 mM EDTA). Beads were resuspended in 200 µL of freshly prepared Elution Buffer (1% SDS, 100 mM $NaHCO_3$) and incubated at 65 °C for 30 mins with shaking. Then, the supernatants were collected and supplemented with 8 µL of NaCl (500 mM) and incubated at 65 °C overnight for reverse crosslinking. 2 µL of RNase A (2 mg/mL) was added and incubated at 37 °C for 1 h, followed by adding 4 µL of Proteinase-K (10 µg/µL) and incubating at 50 °C for 1 h. The released DNA was purified with the QIAquick PCR purification Kit (Qiagen) and processed to quantitative PCR assay or sequencing library preparation. Multiplexed ChIP-seq libraries were prepared with the ChIP DNA matched input DNA by using the NEBNext Ultra II DNA Library Prep Kit (Illumina). The final libraries were checked for quantity and quality and quantified with Qubit and TapeStation (Aglient), and multiplexed libraries were subjected to Illumina Sequencing platforms (available at the UNC HTSF core).

ChIP-seq data analysis

ChIP-seq reads were aligned to both drosophila (dm3) and human (hg19) reference genomes by the BWA (V0.7.12; default parameters) alignment software (*70*). After duplicated reads were removed, MACS2 (v2.1.0; -q 0.1 -, 20 100) (*71*) was used to call peaks with input as controls. Weak peaks with no base covered by at least 10 reads were excluded and peaks overlapping (>= 1 bp) with the "blacklist" regions identified by the ENCODE project (*72*) were also removed. Scaling factors were calculated (*69*) and normalization was done by the bamCompare function of deeptools with bin size 10 and read length 250.

Quantitative reverse transcriptase polymerase chain reaction (RT-qPCR)

RNA was extracted using the RNeasy Mini Kit (Qiagen #74104) according to manufacturer's manual. 1 μg of total RNA was subject to reverse transcription using High Capacity cDNA Reverse Transcription Kits (Applied Biosystems). Then, the real-time PCRs were performed using the Power SYBR Green Master Mix (Thermo Fisher Scientific) and a QuantStudio 7 Flex Real-Time PCR system (Thermo Fisher Scientific). The relative gene expression was calculated with the comparative C_T method by comparing the Ct value of a target gene to that of an internal control such as GAPDH($2^{\triangle\triangle CT}$). The primers used for RT-qPCR are listed in Data File S1.

RNA sequencing (RNA-seq)-based transcriptomic profiling

Transcriptomic profiling was prepared as previously described (*68*). In brief, total RNA was extracted with the RNeasy Mini Kit (Qiagen #74104), and then purified and fragmentized using the commercial kit (NEBNext® Poly(A) mRNA Magnetic Isolation Module, cat# E7490). NEBNext® Ultra™ II RNA Library Prep Kit for Illumina (NEB cat# E7770) was used for cDNA library construction. The final libraries were examined with Qubit and TapeStation (Aglient) and subjected to Illumina Sequencing platforms.

RNA-seq data analysis

RNA-seq data were analyzed as we described previously (*73-75*). In brief, RNA-seq sequencing reads were mapped with MapSplice (*76*) and quantified with RSEM (*77*). Read counts were log2 transformed after upper-quantile normalization. Raw read counts were analyzed by DESeq (*78*) for differential gene expression. Genes with Benjamini-Hochberg (BH) adjusted false discovery rate (FDR) less than 0.05 and the absolute log2 value of fold change greater than 0.58 between the experimental and mock-treated samples (such as gene depletion vs. mock, or drug-treated vs. DMSO) were called as differentially expressed genes (DEGs). GSEA analysis were done using the GESA program (*79*). The ranked list files were generated using the differential expression between the experimental and mock-treated samples. 1000 permutations were applied to determine the enrichment significance for the gene sets.

Proteomics profiling

Four million of MIA PaCa-2 cells were seeded in each one of 10 cm plates and, after the cells became adhered to the dish bottom, they were treated with DMSO or 1.5 μM of MS67 for 2.5 h. Cells were then harvested and washed three times in 1 x PBS, followed by lysis in the Lysis Buffer (8 M urea, 50 mM Tris-HCl, pH 8.0) with usage of the tip probe sonicator (Fisher) to help the lysis process. The resulted lysis samples were centrifuged at maximum speed for 15 mins in a refrigerated microfuge to completely remove cell debris. The protein concentration was measured by Bradford Assay and samples were then frozen at -80 °C until further analysis. For mass spectrometry analysis, 200 μg of each sample was digested with trypsin overnight. After desalting using Waters SepPak C18 cartridges, Pierce Peptide Quantitation assay was performed to monitor the digested peptide concentration. Then 50 μg of each sample was labeled with Tandem Mass Tag (TMT), quenched and combined into a single multiplexed sample. A 100-ug aliquot of the mixed sample was fractionated into 8 fractions using the Pierce High pH Reversed

Phase Fractionation spin columns. Peptide fractions were analyzed on the Easy nLC 1200-QExactive HF for a total of 8 LC/MS/MS runs (carried out in UNC Proteomics Core). Data analysis was performed in Perseus, Excel and GraphPad.

Mouse pharmacokinetic study

MS67 (in its HCl salt form) was dissolved in a solution formulation of 5% NMP, 5% solutol HS-15, and 90% normal saline. Six male Swiss Albino mice were administered intraperitoneally with solution formulation of MS67 at 75 mg/kg. Blood samples (approximately 60 µL) were collected under light isoflurane anesthesia from a set of three mice at 0.5, 1, 2, 4, 8 and 12 h. Plasma was harvested by centrifugation of blood and stored at -70 ± 10 °C until analysis. The plasma concentration-time of MS67 was used for the pharmacokinetic analysis. Plasma samples were quantified by fit-for-purpose LC-MS/MS method (LLOQ: 5.03 ng/mL). Pharmacokinetic analysis was performed using GraphPad Prism software in a way of nonlinear regression analysis. Compound concentrations in plasma at each time point are the average values from 3 test mice. Error bars represent ± SEM.

In vivo efficacy studies

All experiments involving mice were performed according to the Institutional Animal Care and Use Committee (IACUC)-approved protocol. For subcutaneous MV4;11 tumor xenograft experiments, five million of MV4;11 cells, mixed in Matrigel (Corning, 354248), were injected subcutaneously in a volume of 200 µL on both flanks of each 8-week-old mouse (NOD/SCID/gamma(c)(null) mouse, NSG; available from JAX). When the tumor volume reached 100-200 mm^3, mice were randomly grouped as either vehicle or MS67 treatment cohort. Mice were treated with vehicle or MS67 twice daily (BID) by intraperitoneal injections (i.p) at a dose of 75 mg/kg for 5 days per week (from Monday to Friday), and tumor volume was recorded

every 2-3 days via caliper measurement (carried out by the UNC Animal Studies Core). The study was terminated when the tumor size reached the IACUC allowed limit.

For subcutaneous xenograft of AML patient-derived xenograft (PDX) cells, 5 million of MLL-AF9+ AML PDX cells (DFAM-68555-V1; available from PRoXe.org) (*80*), mixed in a volume of 200 μL Matrigel, were injected subcutaneously to both flanks of each 6-8 weeks old mouse (NOD-SCID IL2Rgnull-3/GM/SF mice, NSG-SGM3 mice; available from JAX, stock #013062). When the tumor volume reached to ~60 mm^3, mice were randomly distributed into different treatment group. For the MS67 group, mice were treated with MS67 BID by i.p. at a dose of 100 mg/kg for 5 days per week (from Monday to Friday). For paralleled MS67 and OICR-9429 treatments, the used i.p. dose for MS67 and OICR-9429 were 150 mg/kg and 37.5 mg/kg, respectively, for 5 days per week (BID on Monday, Wednesday and Friday, and SID on Tuesday and Thursday). The matched vehicle was used as a treatment control and tumor volume was recorded every 2-3 days via caliper measurement.

Bioanalysis of MS67 and OICR-9429 in mouse plasma and tumor samples

To determine drug concentrations in plasma and tumor in the in vivo efficacy studies, subcutaneous xenografting of MV4;11 cells (six mice were included in each group) was carried out as described above. Mice were treated with either vehicle or MS67 BID with an i.p. dose of 75 mg/kg for five consecutive days. Tumor and plasma samples were collected from animals at 2 h after the last drug injection. Similar analysis of OICR-9429 was performed with the plasma and tumor samples isolated from the PDX subcutaneous xenograft model, in which mice were treated with OICR-9429 at the i.p. dose of 37.5 mg/kg (BID on Monday, Wednesday and Friday, and SID on Tuesday and Thursday). Tumor and plasma samples were collected at 2 h after the last dose. For plasma preparation, ~200 μL of blood was collected in an Eppendorf tube pre-treated

with EDTA. Samples were centrifuged at 3,000 rpm for 10 min at 4 °C, and the resulting supernatant was transferred to a clean Eppendorf tube and stored at -80 °C. Tumors were harvested immediately after animals were euthanized and then cut into smaller specimens, snap-frozen in liquid nitrogen and stored at -80 °C. All tumor samples were homogenized in 80:20 (vol/vol) water:acetonitrile at a 1:9 (wt/vol) ratio. Total homogenization dilution was 10×. All tumor samples were diluted 5× in plasma and analyzed against plasma calibration curves. MS67 or OICR-9429 concentrations in plasma and tumor samples were analyzed using LC-MS. Waters UPLC BEH C18 column (2.1 × 50 mm, 1.7 μm) was used for LC. Mobile phase A: 95:5:0.1 (vol/vol/vol) water:acetonitrile:formic acid. Mobile phase B: 50:50:0.1 (vol/vol/vol) methanol:acetonitrile:formic acid. API 6500 was used for MS/MS analysis. Electrospray was used for the ionization method (positive ion).

Culture of human patient-derived AML cells

Primary human AML cells were cultured as previously described (*81*). Briefly, the frozen AML cells of de-identified patients (collected by the UNC Tissue Procurement Facility [TPF]; cryopreserved in 10% of DMSO) were quickly thawed, resuspended in 40 mL of DMEM with 20% FBS, and collected after centrifugation at 600 g for 5 mins. Next, cells were seeded at a density of 2-5 x 10^5 cells/mL in 24-well plates that were already plated with irradiated stromal cells (Hs27, ATCC, CRL-1634), and then cultured in the DMEM base medium supplemented with 15% of FBS, 1 x penicillin/streptomycin, 50 μM of β-mercaptoethanol, and a set of human cytokines including 100 ng/mL SCF (PeproTech, 300-07), 10 ng/mL FLT3L (PeproTech, 300-19), 10 ng/mL TPO (PeproTech, 300-18), 10 ng/mL IL3 (PeproTech, 200-03) and 20 ng/mL IL-6 (PeproTech, 200-06).

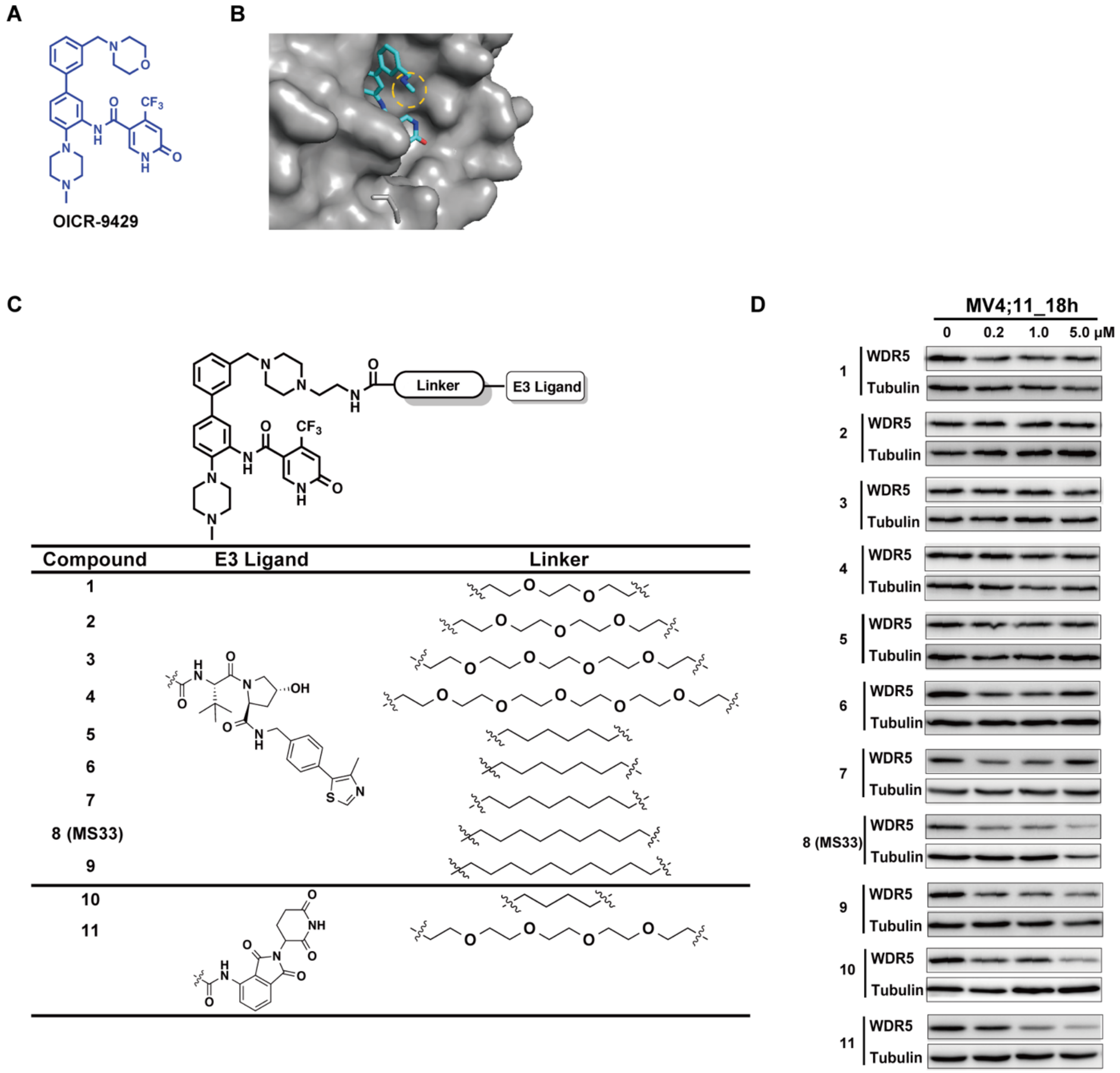

Fig. S1. Design and structure-activity relationship results of initial WDR5 degraders. (**A**) Chemical structure of the WDR5 inhibitor OICR-9429. (**B**) Cocrystal structure of WDR5 in complex with OICR-9429 (PDB ID:4QL1). The yellow dotted circle highlights a small portion of OICR-9429's morpholine group, which is solvent-exposed. (**C**) Chemical structures of initially designed WDR5 degraders **1**-**11**. (**D**) Immunoblots for WDR5 and Tubulin post-treatment of MV4;11 cells with the indicated compounds for 18 h.

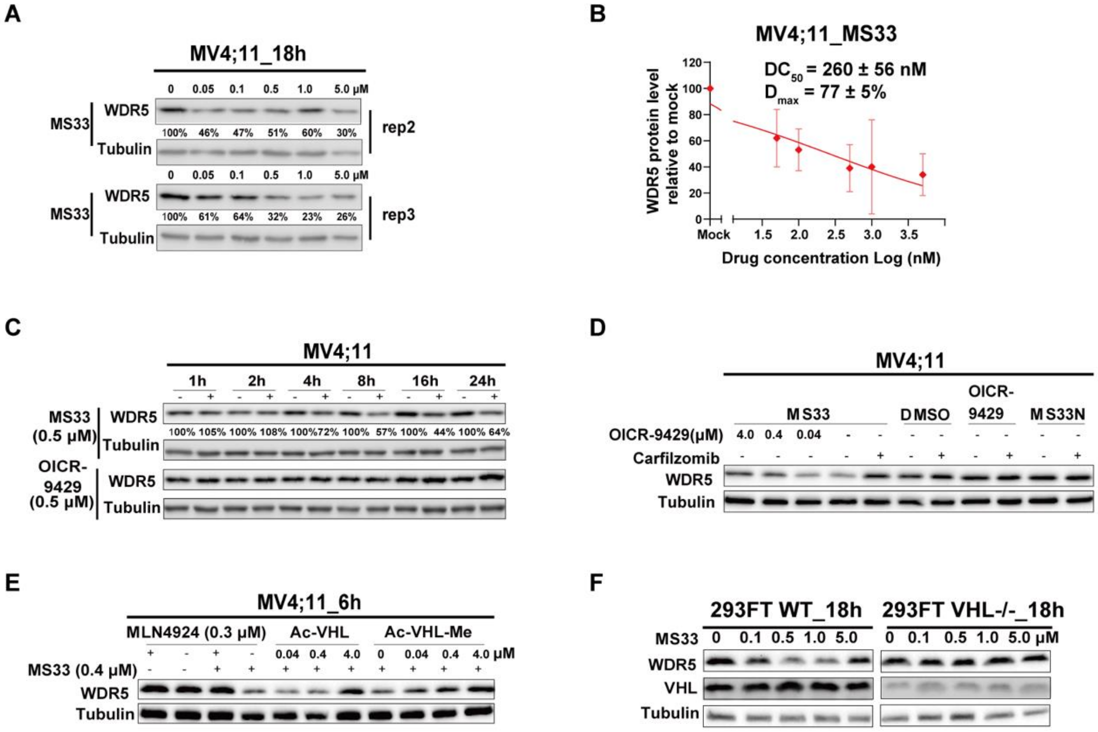

Fig. S2. Biochemical characterization of the WDR5 degrader MS33. (**A**) Two additional immunoblots for WDR5 and Tubulin post-treatment of MV4;11 cells with the indicated concentrations of MS33 for 18 h used for determining the DC_{50} and D_{max} values of MS33. (**B**) DC_{50} and D_{max} values of MS33 in MV4;11 cells are shown as the mean ± SD from three independent experiments. MV4;11 cells were treated with MS33 for 18 h. The band intensity is determined by Image J software. (**C**) Immunoblots for WDR5 and Tubulin post-treatment of MV4;11 cells with 0.5 μM of MS33 or OICR-9429 for the indicated time. (**D**) Immunoblots for WDR5 and Tubulin after a 2-hour pre-treatment with DMSO, carfilzomib (0.4 μM) or OICR-9429 (0.04 μM, 0.4 μM, 4.0 μM), followed by a 6-hour treatment with 0.4 μM of MS33 in MV4;11 cells. (**E**) Immunoblots for WDR5 and Tubulin after a 2-hour pre-treatment with DMSO, MLN4924 (0.3 μM) or Ac-VHL/Ac-VHL-Me (0.04 μM, 0.4 μM, 4 μM), followed by a 6-hour treatment with 0.4 μM of MS33 in MV4;11 cells. (**F**) Immunoblots for WDR5, Tubulin and VHL post-treatment of 293FT, either wildtype (WT) or after the CRISPR/Cas9-mediated knockout (KO) of VHL, with the indicated concentrations of MS33 for 18 h.

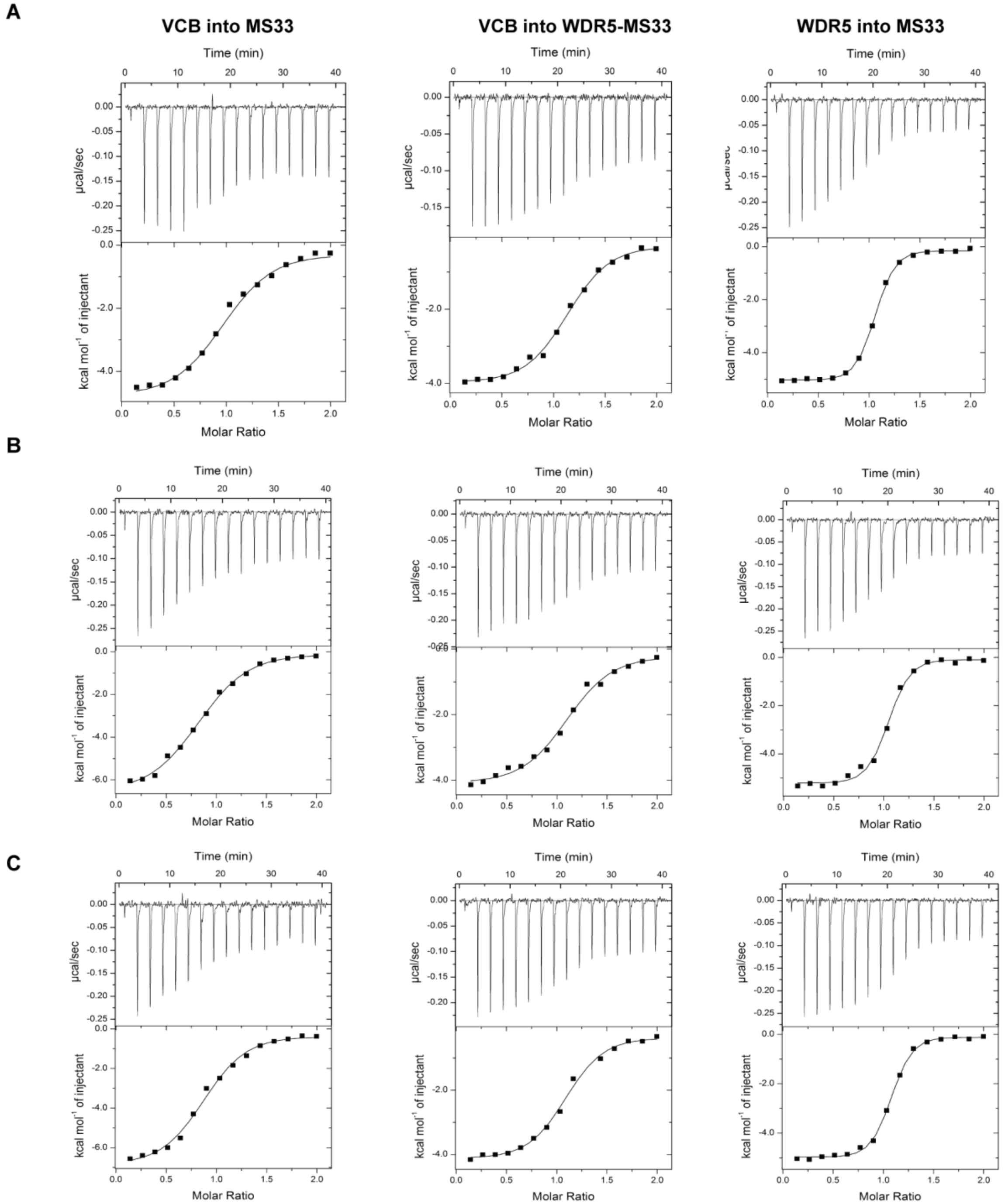

VCB into MS33		VCB into WDR5-MS33		WDR5 into MS33	
N (sites)	1.2 ± 0.09	N (sites)	0.89 ± 0.02	N (sites)	0.99 ± 0.02
K_d (nM)	870 ± 76	K_d (nM)	520 ± 34	K_d (nM)	120 ± 7
ΔH (cal/mol)	-5590 ± 760	ΔH (cal/mol)	-4781 ± 68	ΔH (cal/mol)	-5229 ± 40
ΔS (cal/mol/deg)	9.0 ± 2.6	ΔS (cal/mol/deg)	12.7 ± 0.2	ΔS (cal/mol/deg)	14.0 ± 0.3
		α	1.66		

Fig. S3. Inverse ITC titrations of VCB into MS33 and MS33-WDR5 complex. Three replicates of inverse Isothermal titration calorimetry (ITC) titrations are shown for von Hippel-Lindau Elongin C-Elongin B (VCB) into MS33 (left), VCB into MS33-WDR5 complex (middle) and WDR5 into MS33 (right) for measuring binding kinetic and determining cooperativity (α) for MS33. The calculated values represent the mean ± SD from three independent experiments. First injection has been removed from the fitting.

A

WDR5 into MS33N

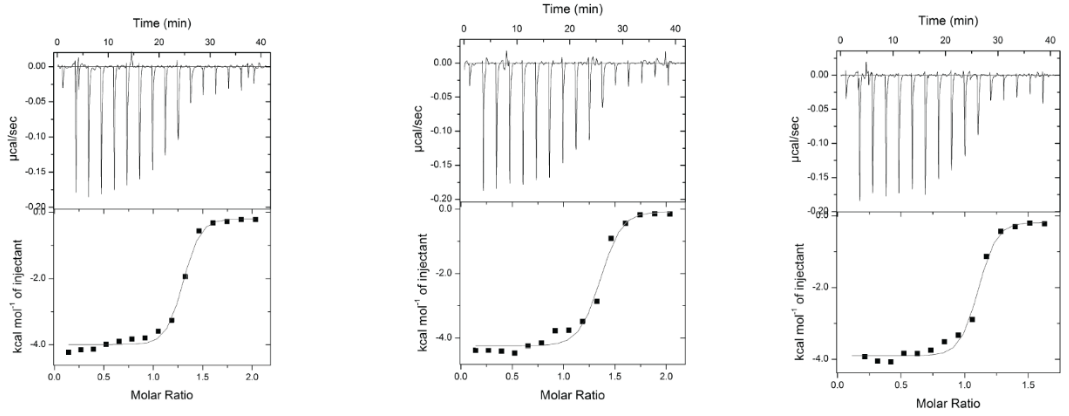

N (sites) **0.88 ± 0.17**
K_d (nM) **86 ± 3.4**
ΔH (cal/mol) **-4905 ± 817**
ΔS (cal/mol/deg) **15.9 ± 2.7**

B

VCB into MS33N

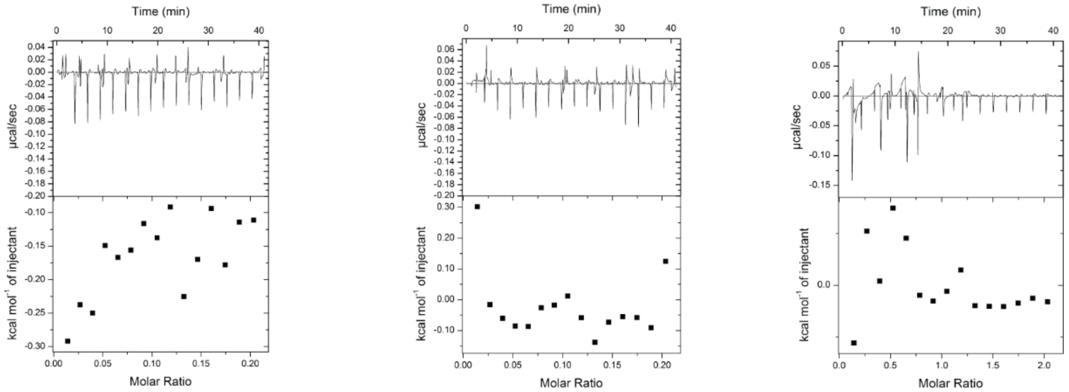

Fig. S4. Inverse ITC titrations of WDR5 and VCB into MS33N. Three replicates of inverse ITC titrations are shown for WDR5 into MS33N (**A**) and VCB into MS33N (**B**) for measuring binding kinetic. The calculated values represent the mean ± SD from three independent experiments. First injection has been removed from the fitting.

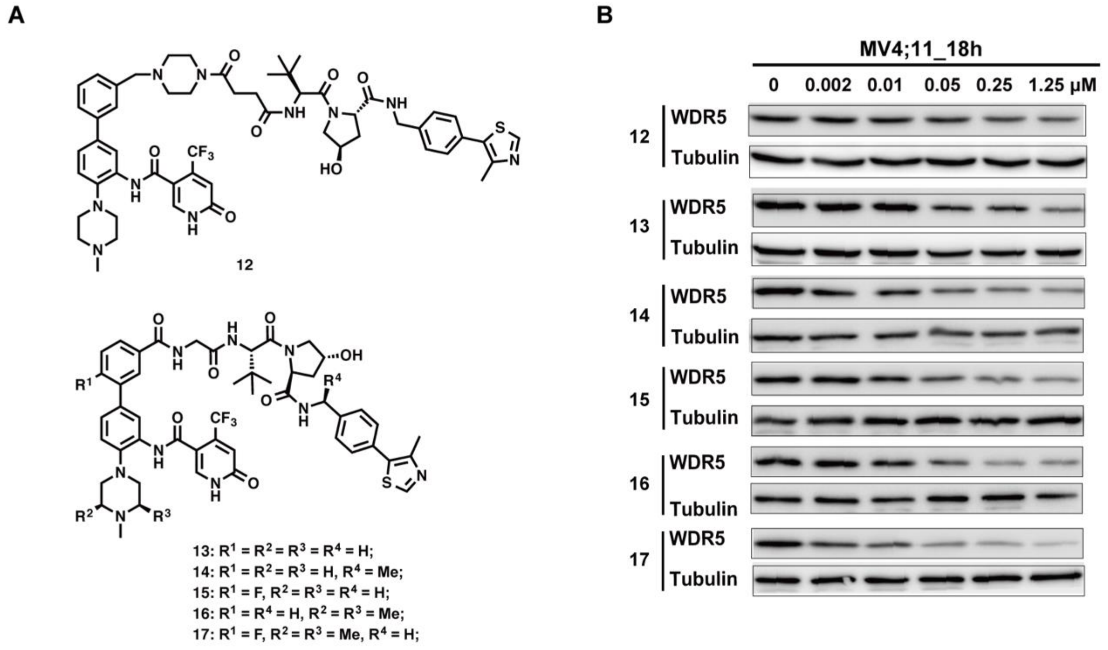

Fig. S5. Structure-activity relationship results of the WDR5 degraders designed based on the WDR5-MS33-VCB ternary complex structure. (**A**) Chemical structures of designed WDR5 degraders **12**-**17**. (**B**) Immunoblots for WDR5 and Tubulin post-treatment of MV4;11 cells with the indicated compounds for 18 h.

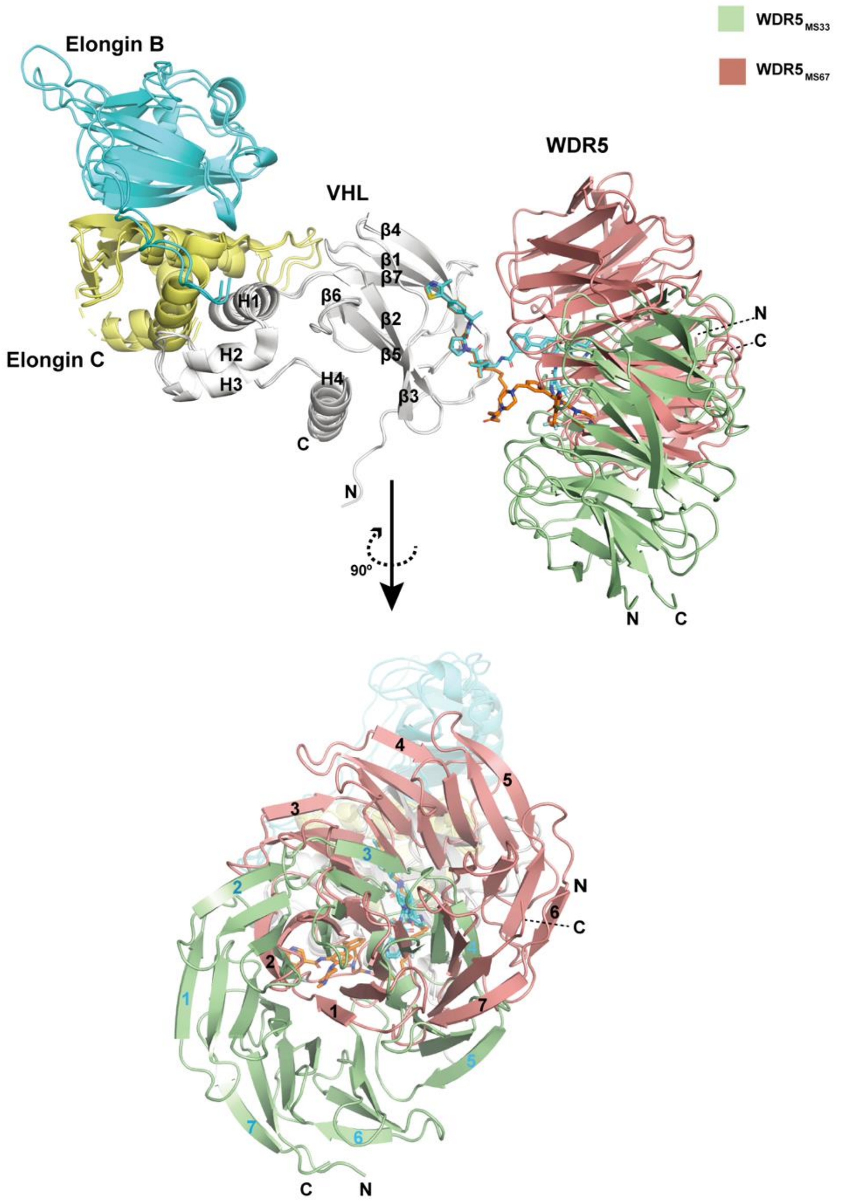

Fig. S6. Alignment of the VCB-MS33-WDR5 complex with the VCB-MS67-WDR5 complex. Superposition of two structures (with respect to VCB) reveals a large difference in the orientation of WDR5. $WDR5_{MS67}$ (pale green) undergoes a large rotation and translation in the direction of the loop between β4 and β5 of VHL, relative to WDR5-MS33 (salmon). $WDR5_{MS33}$ is colored in salmon and $WDR5_{MS67}$ is colored in pale green.

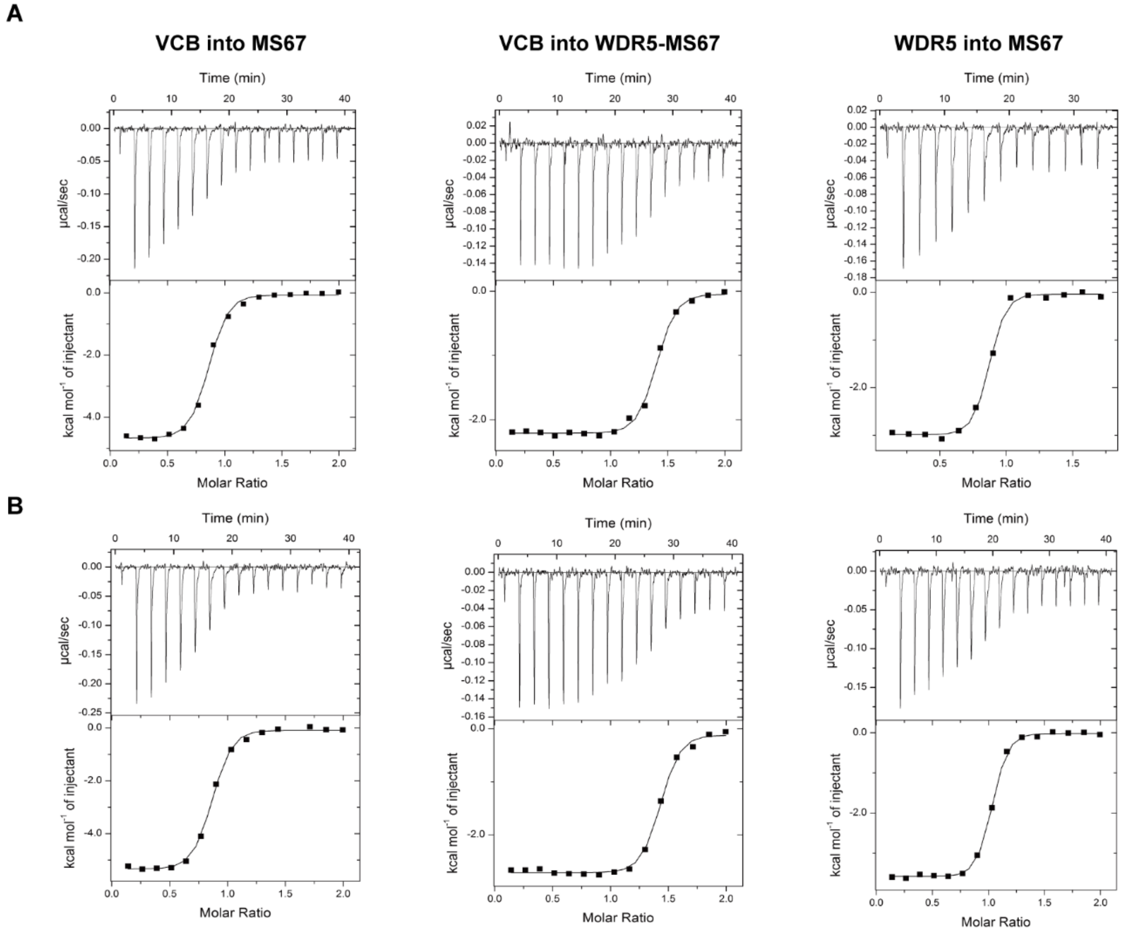

Fig. S7. Inverse ITC titrations of VCB into MS67 and MS67-WDR5 complex. Two additional replicates of inverse ITC titrations are shown for VCB into MS67 (left), VCB into MS67-WDR5 complex (middle) and WDR5 into degrader MS67 (right) for measuring binding kinetic and determining cooperativity (α) for MS67. The calculated values represent the mean ± SD from three independent experiments. First injection has been removed from the fitting.

A

WDR5 into MS67N

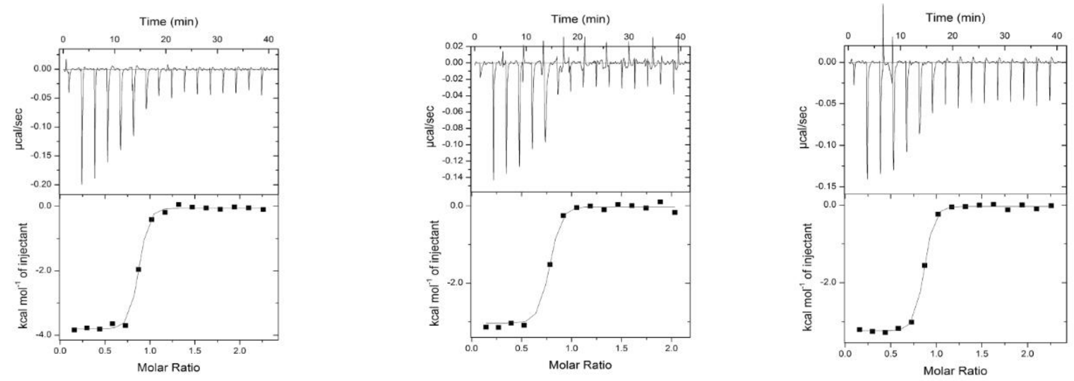

**N (sites)**	**1.25 ± 0.01**
***K*$_d$ (nM)**	**47 ± 3.4**
**ΔH (cal/mol)**	**-2735 ± 318**
**ΔS (cal/mol/deg)**	**24.4 ± 1.1**

B

VCB into MS67N

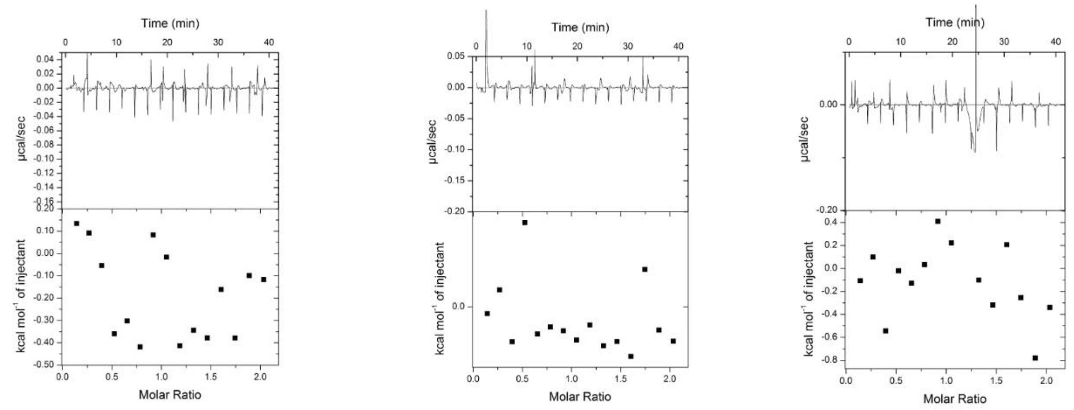

Fig. S8. Inverse ITC titrations of WDR5 and VCB into MS67N. Three replicates of inverse ITC titrations are shown for WDR5 into MS67N (**A**) and VCB into MS67N (**B**) for measuring binding kinetic. The calculated values represent the mean ± SD from three independent experiments. First injection has been removed from the fitting.

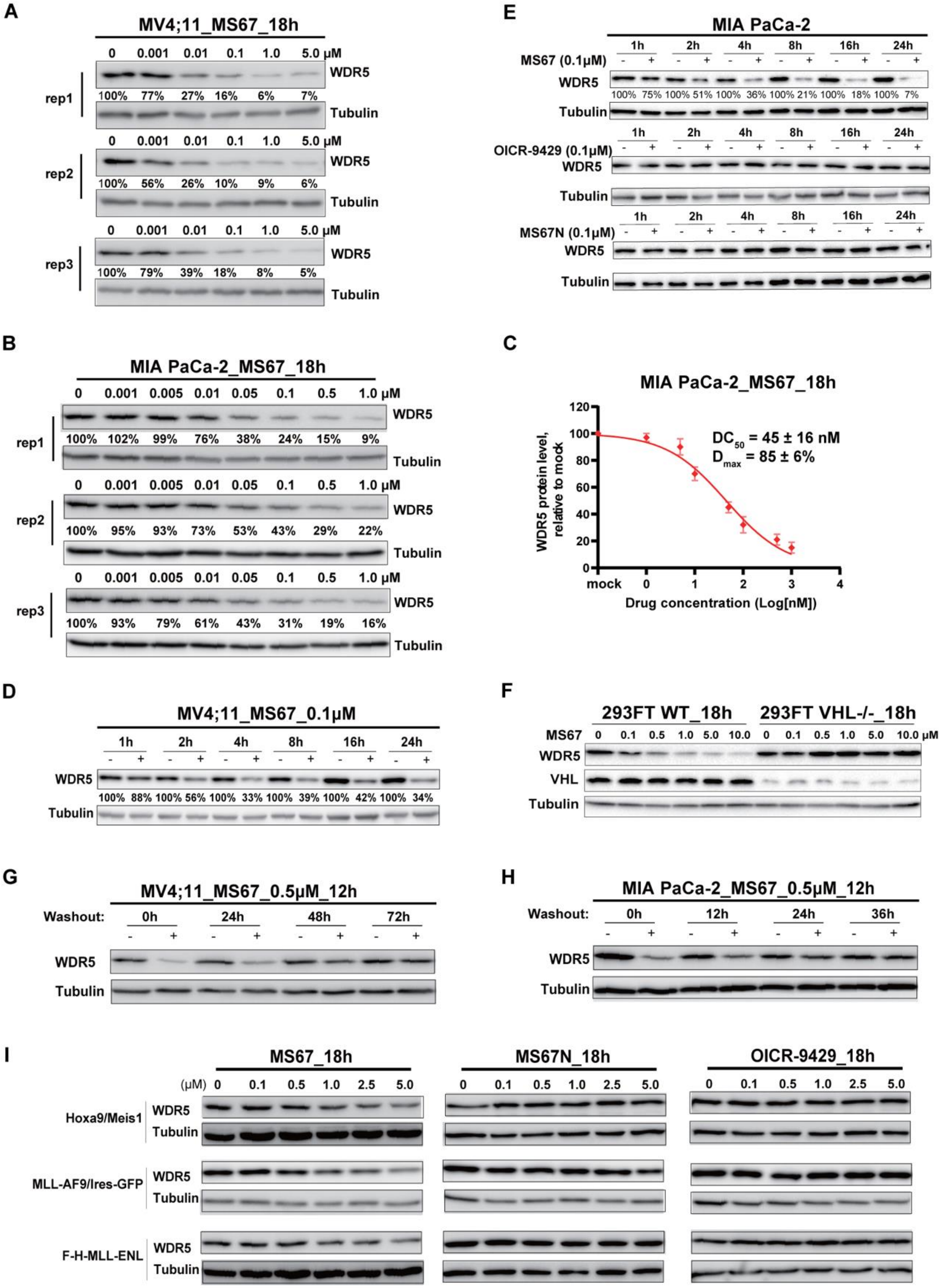

Fig. S9. WDR5 degradation effects of MS67 in vitro. (**A-B**) Representative immunoblots used for determining the DC_{50} and D_{max} values of MS67 in MV4;11 cells (**A**) and MIA PaCa-2 cells (**B**). MV4;11 and MIA PaCa-2 cells were treated with MS67 at the indicated concentrations for 18 h. The band intensity is determined by Image J software. (**C**) DC_{50} and D_{max} values of MS67

in MIA PaCa-2 cells are shown as the mean ± SD from the three independent experiments in (**B**). (**D**) Immunoblots for WDR5 and Tubulin post-treatment of MV4;11 cells with 0.1 μM of MS67 for the indicated time. (**E**) Immunoblots for WDR5 and Tubulin post-treatment of MIA PaCa-2 cells with 0.1 μM of MS67, MS67N or OICR-9429 for the indicated time. (**F**) Immunoblots for WDR5, Tubulin and VHL post-treatment of 293FT, either WT or after CRISPR/Cas9-mediated KO of VHL, with the indicated concentrations of MS67 for 18 h. (**G-H**) Immunoblots for WDR5 and Tubulin post-treatment of MV4;11 cells (**G**) and MIA PaCa-2 cells (**H**) with 0.5 μM of MS67 for 12 h and washed with fresh medium for the indicated time. (**I**) Immunoblots for WDR5 and Tubulin post-treatment of murine AML cells, which were established by Hoxa9 plus Meis1 (Hoxa9/Meis1), MLL-AF9 (in a bicistronic Ires-GFP vector) or Flag-HA-tagged MLL-ENL oncogene, with DMSO or the indicated concentrations of MS67, MS67N or OICR-9429 for 18 h.

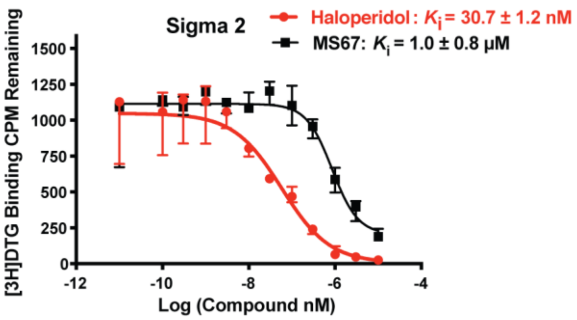

Fig. S10. Concentration response curve of MS67 binding to Sigma 2 receptor. The radioligand binding assay was performed with MS67 at serial-diluted concentrations in triplicate. The K_i value of MS67 binding to Sigma 2 receptor was calculated using the Sigma 2 ligand haloperidol as the control.

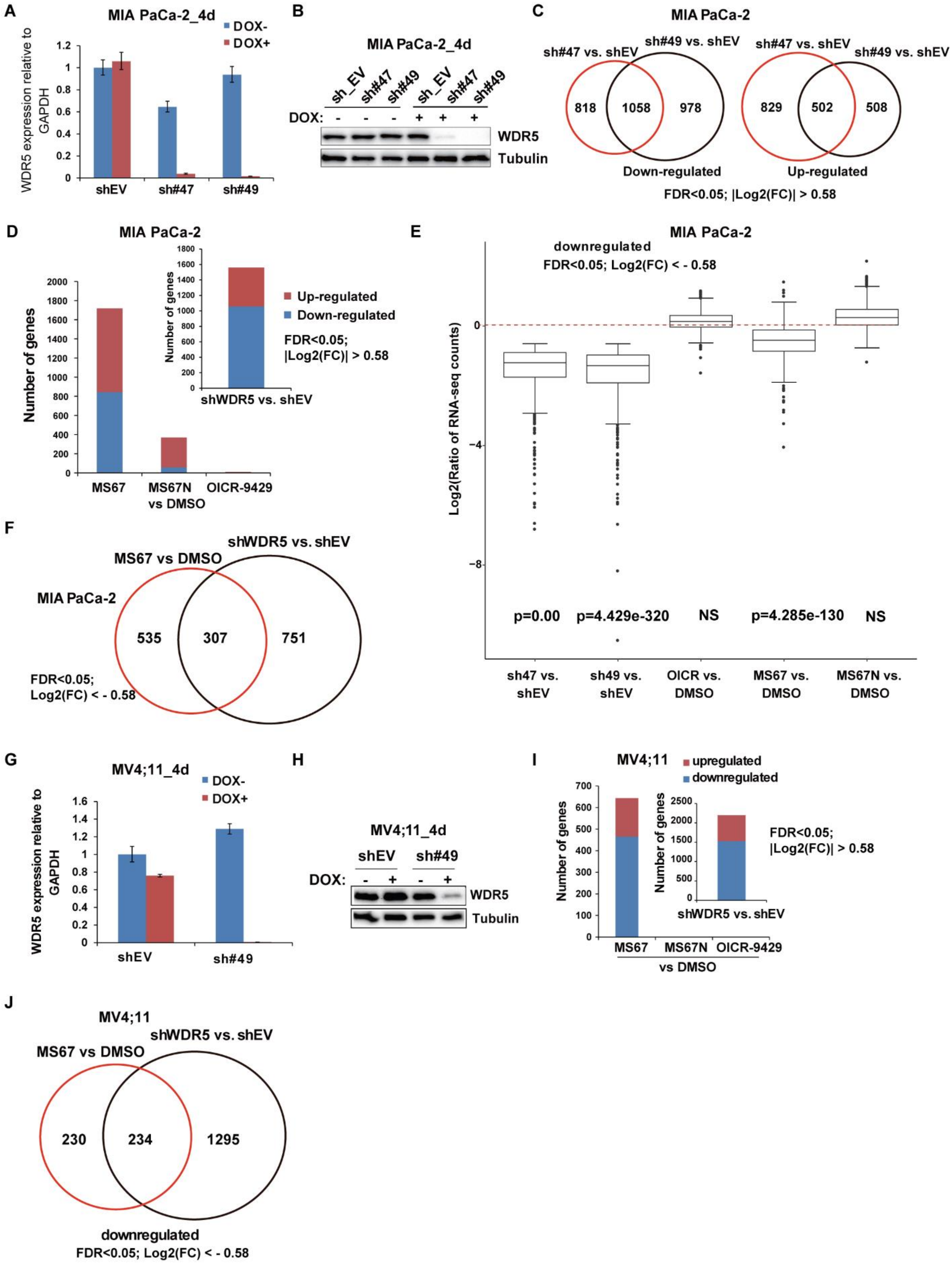

A
MIA PaCa-2_4d
DOX-
DOX+
WDR5 expression relative to GAPDH
shEV
sh#47
sh#49
B
MIA PaCa-2_4d
sh_EV
sh#47
sh#49
DOX:
WDR5
Tubulin
C
MIA PaCa-2
sh#47 vs. shEV
sh#49 vs. shEV
818
1058
978
829
502
508
Down-regulated
Up-regulated
FDR<0.05; |Log2(FC)| > 0.58
D
MIA PaCa-2
Number of genes
Up-regulated
Down-regulated
FDR<0.05; |Log2(FC)| > 0.58
shWDR5 vs. shEV
MS67
MS67N
vs DMSO
OICR-9429
E
MIA PaCa-2
downregulated
FDR<0.05; Log2(FC) < - 0.58
Log2(Ratio of RNA-seq counts)
p=0.00
p=4.429e-320
NS
p=4.285e-130
NS
sh47 vs. shEV
sh49 vs. shEV
OICR vs. DMSO
MS67 vs. DMSO
MS67N vs. DMSO
F
shWDR5 vs. shEV
MS67 vs DMSO
MIA PaCa-2
535
307
751
FDR<0.05; Log2(FC) < - 0.58
G
MV4;11_4d
DOX-
DOX+
WDR5 expression relative to GAPDH
shEV
sh#49
H
MV4;11_4d
shEV
sh#49
DOX:
WDR5
Tubulin
I
MV4;11
upregulated
downregulated
Number of genes
shWDR5 vs. shEV
FDR<0.05; |Log2(FC)| > 0.58
MS67
MS67N
OICR-9429
vs DMSO
J
MV4;11
shWDR5 vs. shEV
MS67 vs DMSO
230
234
1295
downregulated
FDR<0.05; Log2(FC) < - 0.58

Fig. S11. RNA-seq profiling of MV4;11 or MIA PaCa-2 cells post-knockdown (KD) of WDR5 or post-treatment with WDR5 inhibitor or degrader. (**A** and **B**) RT-qPCR (**A**) and immunoblots (**B**; with Tubulin as a loading control) for WDR5 in MIA PaCa-2 cells with stable expression of a doxycycline-inducible shRNA targeting WDR5 (sh#47 or sh#49) or empty vector (shEV), after treatment with either vehicle (DOX-) or doxycycline (DOX+; 0.5 μg/mL) for 4 days. (**C**) Venn diagram showing the overlap for differentially expressed genes (DEGs), either down-regulated (left) or up-regulated (right), identified by WDR5 KD with two independent shRNAs (sh#47 and sh#49) in MIA PaCa-2 cells. DEG is determined with a cut-off of the absolute value for Log2(FC) greater than 0.58 and FDR value less than 0.05. (**D**) Bar charts showing the total number of DEGs, either down-(blue) or up-regulated (red), as identified by RNA-seq in MIA PaCa-2 cells post-treatment with MS67, MS67N or OICR-9429 in comparison to DMSO (bottom panel), or post-KD of WDR5 relative to mock (upper). (**E**) Box plots showing the Log2 ratios for down-regulated DEGs due to WDR5 shRNA (sh#47 and sh#49 versus shEV; two left panels) in MIA PaCa-2 cells. Comparison was done across sh#47 versus shEV, sh#49 versus shEV, OICR-9429 versus DMSO, MS67N versus DMSO and MS67 versus DMSO. *P* value was generated for each comparison. (**F**) Venn diagram showing the overlap between down-regulated DEGs due to treatment of MIA PaCa-2 cells with MS67 (red) and the 1,058 common down-regulated DEGs due to WDR5 KD by sh#47 and sh#49 (black), relative to mock. (**G** and **H**) RT-qPCR (G) and immunoblots (**H**; with Tubulin as a loading control) for WDR5 in MV4;11 cells with stable expression of a doxycycline-inducible shRNA targeting WDR5 (sh#49) or shEV, after treatment with either vehicle (DOX-) or doxycycline (DOX+) for 4 days. (**I**) Bar charts showing the total number of DEGs, either down-(blue) or up-regulated (red), as identified by RNA-seq in MV4;11 cells post-treatment with MS67, MS67N or OICR-9429 (bottom panel) or post-KD of WDR5 (upper; sh#49), relative to mock. (**J**) Venn diagram showing the overlap between down-regulated DEGs due to treatment of MV4;11 cells with MS67 (red) and those due to WDR5 KD with sh#49 (black), relative to mock.

A

MIA PaCa-2 MS67_1 μM_6d

GENESET	NES	NOM p-val	FDR q-val
MIAPACA2_WDR5_KD(SH47)_DOWN_REGULATED	-3.304	0	0
MIAPACA2_WDR5_KD(SH49)_DOWN_REGULATED	-3.125	0	0
GO_CYTOSOLIC_LARGE_RIBOSOMAL_SUBUNIT	-2.460	0	0
GO_CYTOSOLIC_RIBOSOME	-2.383	0	0
WDR5 BINDING_CARUGO_ET_AL_	-2.311	0	0
GO_NEURAL_CREST_CELL_MIGRATION	-1.966	0	0.03
GO_POLYSOMAL_RIBOSOME	-1.918	0	0.04
GO_TRANSLATIONAL_INITIATION	-1.886	0	0.05

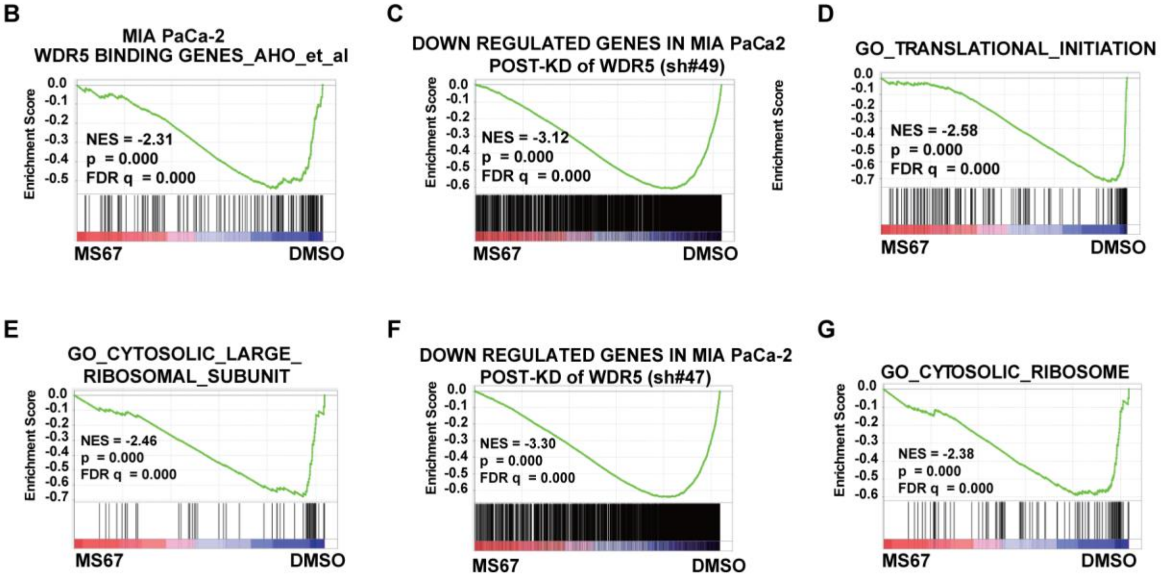

Fig. S12. GSEA analysis of RNA-seq data in MIA PaCa-2 cells. (**A**) Summary of the significantly enriched gene sets based on GSEA using the RNA-seq data in MIA PaCa-2 cells treated with 1 μM of MS67 for 6 days, relative to mock. (**B-G**) GSEA analysis examples showing correlation analysis between the indicated gene sets in MIA PaCa-2 cells treated with MS67 (1 μM for 6 days).

A

MV4;11 MS67_0.1μM_3d

GENESET	NES	NOM p-val	FDR q-val
MV4;11_WDR5_KD(SH49)_DOWN_REGULATED	-3.440	0	0
WDR5 BINDING_CARUGO_ET_AL_	-2.918	0	0
GO_CYTOSOLIC_RIBOSOME	-2.862	0	0
GO_CYTOSOLIC_LARGE_RIBOSOMAL_SUBUNIT	-2.834	0	0
GO_STEROL_BIOSYNTHETIC_PROCESS	-2.671	0	0
GO_TRANSLATIONAL_INITIATION	-2.583	0	0
GO_REGULATION_OF_LIPID_BIOSYNTHETIC_PROCESS	-2.575	0	0
GO_STRUCTURAL_CONSTITUENT_OF_RIBOSOME	-2.530	0	0
YU_MYC_TARGETS_UP	-2.414	0	0
HALLMARK_E2F_TARGETS	-2.350	0	0
MIAPACA2_WDR5_KD(SH47)_DOWN_REGULATED	-2.299	0	0
GO_POLYSOMAL_RIBOSOME	-2.258	0	2.47E-05
HALLMARK_G2M_CHECKPOINT	-2.235	0	9.46E-05
GO_CYTOSOLIC_SMALL_RIBOSOMAL_SUBUNIT	-2.154	0	8.74E-04
HALLMARK_MTORC1_SIGNALING	-2.132	0	0.001
MIAPACA2_WDR5_KD(SH49)_DOWN_REGULATED	-2.124	0	0.001
HALLMARK_HYPOXIA	-2.010	0	0.005
GO_DNA_REPLICATION	-1.968	0	0.009

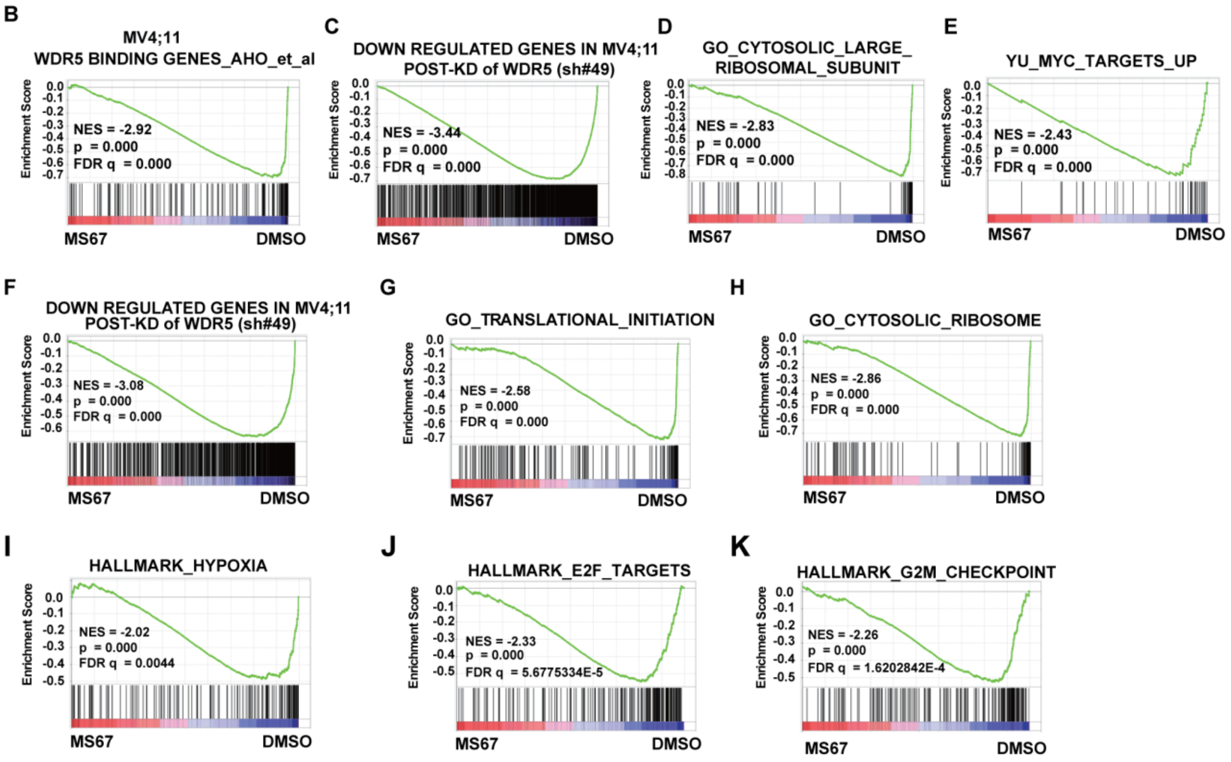

Fig. S13. GSEA analysis of RNA-seq data in MV4;11 cells. (**A**) Summary of the significantly enriched gene sets based on GSEA using the RNA-seq data in MV4;11 cells treated with 0.1 μM of MS67 for 3 days, relative to mock. (**B-K**) GSEA analysis examples showing correlation analysis between the indicated gene sets in MV4;11 cells treated with MS67 (0.1 μM for 3 days).

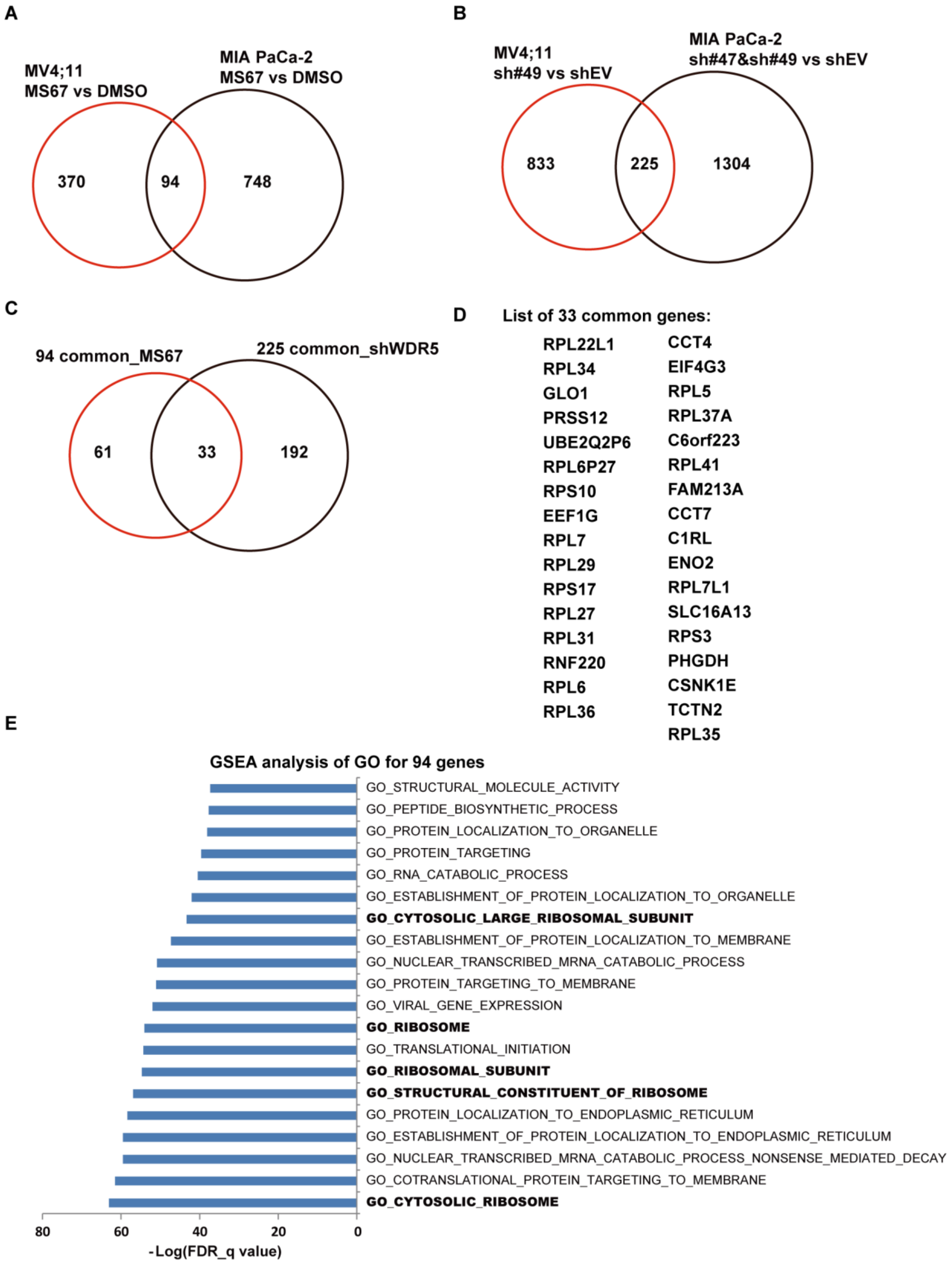

Fig. S14. Overlap of down-regulated genes in MV4;11 and MIA PaCa-2 cells. (**A**) Venn diagram showing the total number and overlap of down-regulated DEGs due to treatment with MS67 in MV4;11 cells (red) and MIA PaCa-2 cells (black). (**B**) Venn diagram showing the total

number and overlap of down-regulated DEGs due to WDR5 KD in MV4;11 cells (sh#49, red) and MIA PaCa-2 cells (common from sh#47 and sh#49, black). (**C**) Venn diagram showing the overlap of common down-regulated DEGs from (**A**) and (**B**). (**D**) Gene list of the 33 common genes from (**C**). (**E**) GO analysis of the overlapped down-regulated DEGs from (**A**).

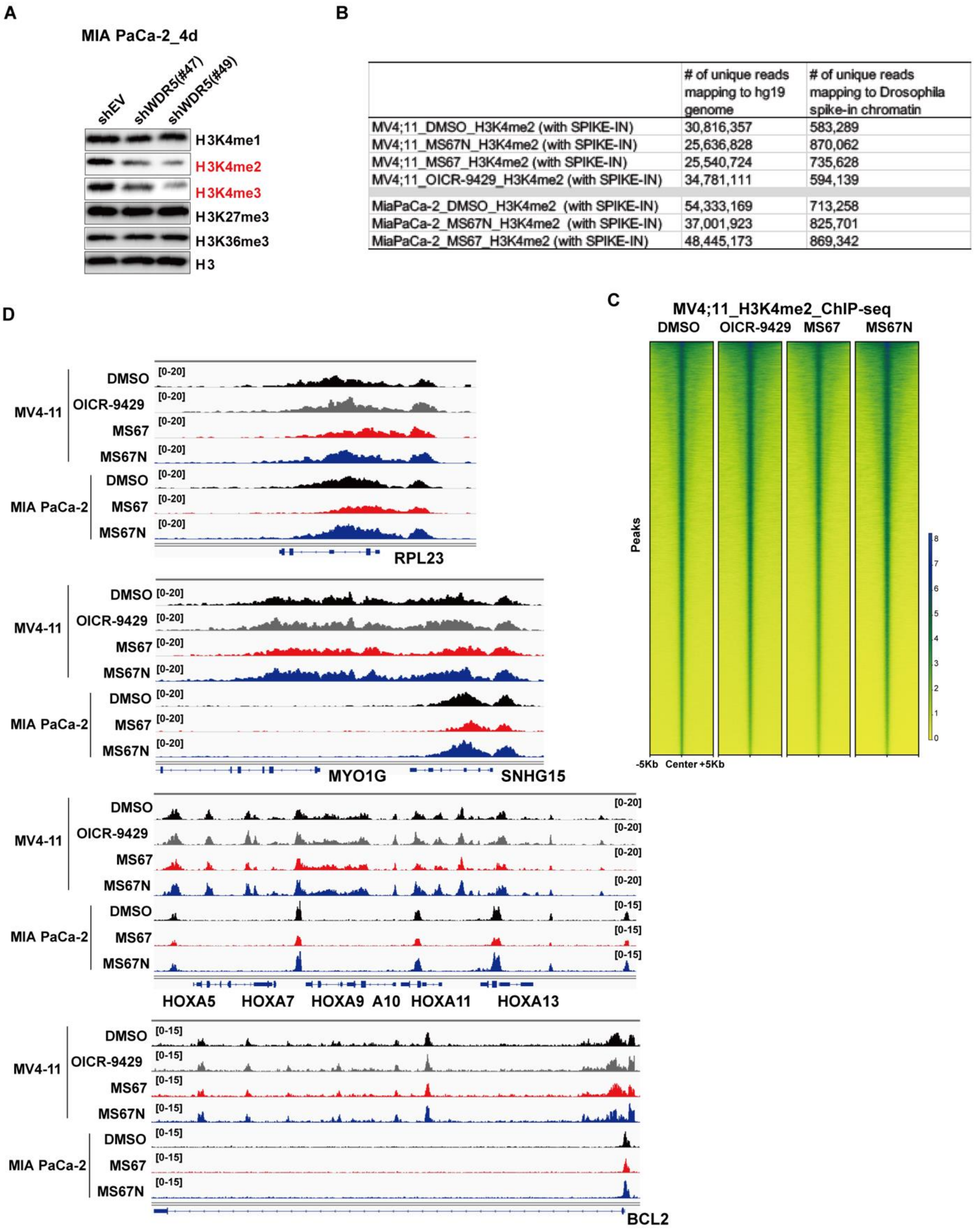

	# of unique reads mapping to hg19 genome	# of unique reads mapping to Drosophila spike-in chromatin
MV4;11_DMSO_H3K4me2 (with SPIKE-IN)	30,816,357	583,289
MV4;11_MS67N_H3K4me2 (with SPIKE-IN)	25,636,828	870,062
MV4;11_MS67_H3K4me2 (with SPIKE-IN)	25,540,724	735,628
MV4;11_OICR-9429_H3K4me2 (with SPIKE-IN)	34,781,111	594,139
MiaPaCa-2_DMSO_H3K4me2 (with SPIKE-IN)	54,333,169	713,258
MiaPaCa-2_MS67N_H3K4me2 (with SPIKE-IN)	37,001,923	825,701
MiaPaCa-2_MS67_H3K4me2 (with SPIKE-IN)	48,445,173	869,342

Fig. S15. MS67 treatment leads to global decrease of H3K4 methylation in vitro. (**A**) Immunoblots for the indicated histone modifications (with H3 as a loading control) in MIA PaCa-2 cells expressing vector control (shEV) or an inducible shRNA targeting WDR5 (sh#47 or sh#49) post-treatment with 0.5 µg/mL doxycycline treatment for 4 days. (**B**) Summary of counts

of reads uniquely mapped to human (hg19) or Drosophila (dm3; spike-in control) in the indicated H3K4me2 spike-in ChIP-seq experiments using MV4;11 and MIA PaCa-2 cells, treated with DMSO, OICR-9429, MS67N or MS67. (**C**) Heatmap showing the spike-in-normalized H3K4me2 ChIP-seq signal density, -/+5Kb around the called peaks in the mock condition, in MV4;11 cells treated with DMSO (mock) or 0.1 μM of OICR-9429, MS67 or MS67N for 3 days. (**D**) Integrative genomics viewer (IGV) views of the indicated genes showing the decreased H3K4me2 by treatment of MV4;11 (0.1 μM for 3 days) and MIA PaCa-2 cells (2 μM for 8 days) with MS67 in comparison with DMSO, OICR-9429 or MS67N treated.

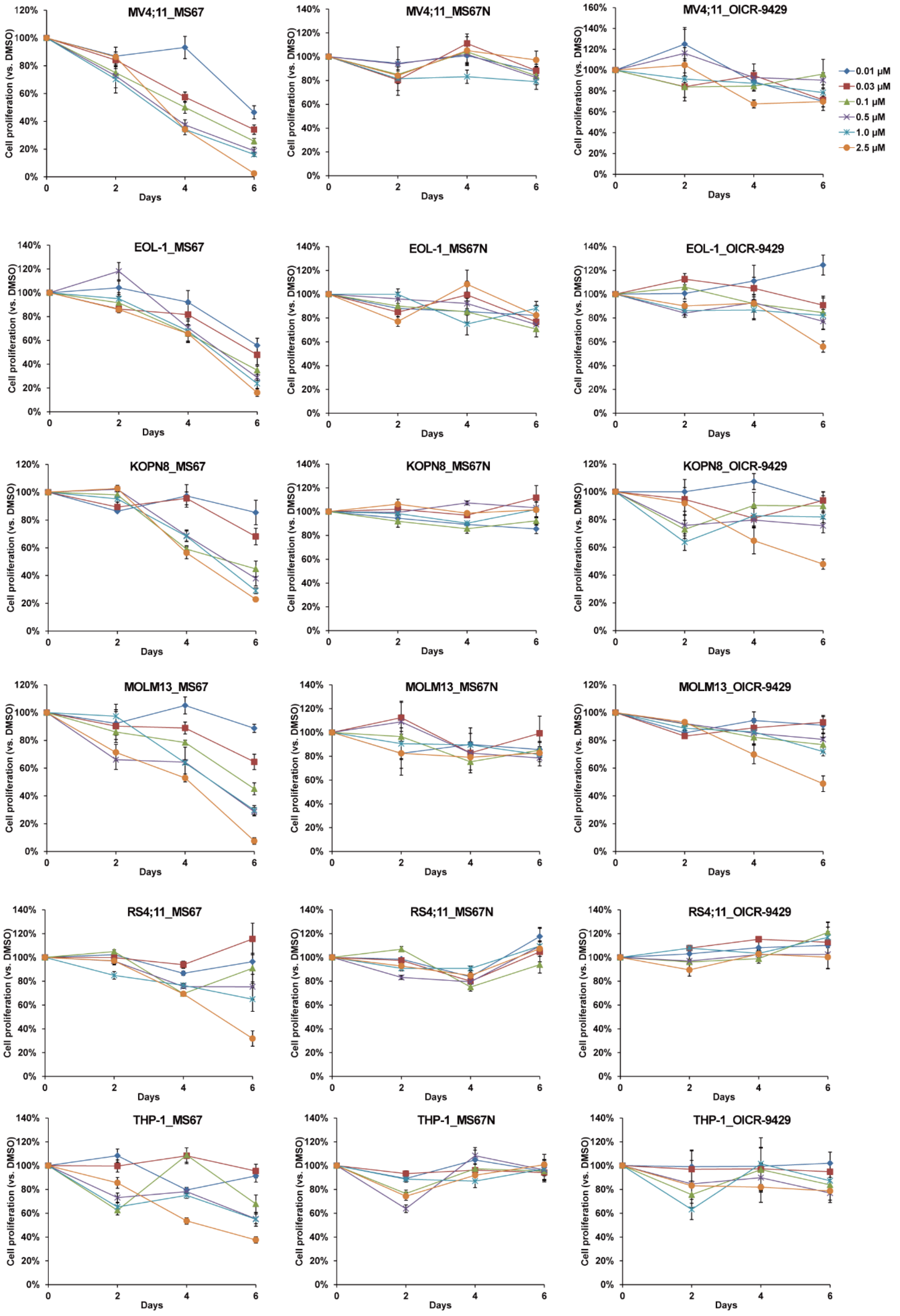
MV4;11_MS67
MV4;11_MS67N
MV4;11_OICR-9429
EOL-1_MS67
EOL-1_MS67N
EOL-1_OICR-9429
KOPN8_MS67
KOPN8_MS67N
KOPN8_OICR-9429
MOLM13_MS67
MOLM13_MS67N
MOLM13_OICR-9429
RS4;11_MS67
RS4;11_MS67N
RS4;11_OICR-9429
THP-1_MS67
THP-1_MS67N
THP-1_OICR-9429
0.01 µM
0.03 µM
0.1 µM
0.5 µM
1.0 µM
2.5 µM
Cell proliferation (vs. DMSO)
Days

Fig. S16. MS67 inhibits the growth of human leukemia cell lines. Growth inhibition curves of MS67 in human leukemia cells, MV4;11, EOL-1, KOPN8, MOLM13, RS4;11, and THP-1. Y-axis, presented in the mean ± SEM of data from three independent experiments, shows the relative cell number post-treatment with MS67 at the indicated concentrations and days (x-axis), normalized to DMSO-treated.

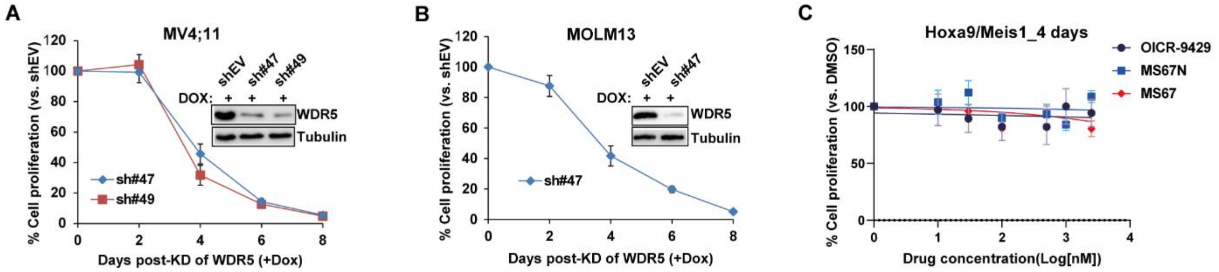

Fig. S17. The effect of WDR5 KD, MS67, MS67N and OICR-9429 on cell growth inhibition in leukemia cells. (**A-B**) Cell proliferation of MV4;11 (**A**) and MOLM13 (**B**) cells bearing a WDR5-targeting shRNA (sh#47 or #49) or empty vector (shEV), as determined by cell counting. Immunoblots for WDR5 and Tubulin in MV4;11 (**A**) and MOLM13 (**B**) cells with stable expression of a doxycycline-inducible shRNA targeting WDR5 (sh#47 or #49) or shEV, after treatment with doxycycline (DOX+; 0.5 µg/mL) for 4 days. (**C**) Growth inhibition curves of MS67, MS67N and OICR-9429 in murine AML cells established by Hoxa9/Meis1. Y-axis, presented in the mean ± SEM of data from three independent experiments, shows the relative cell number post-treatment with the indicated concentrations (x-axis) of compounds for 4 days, normalized to DMSO-treated.

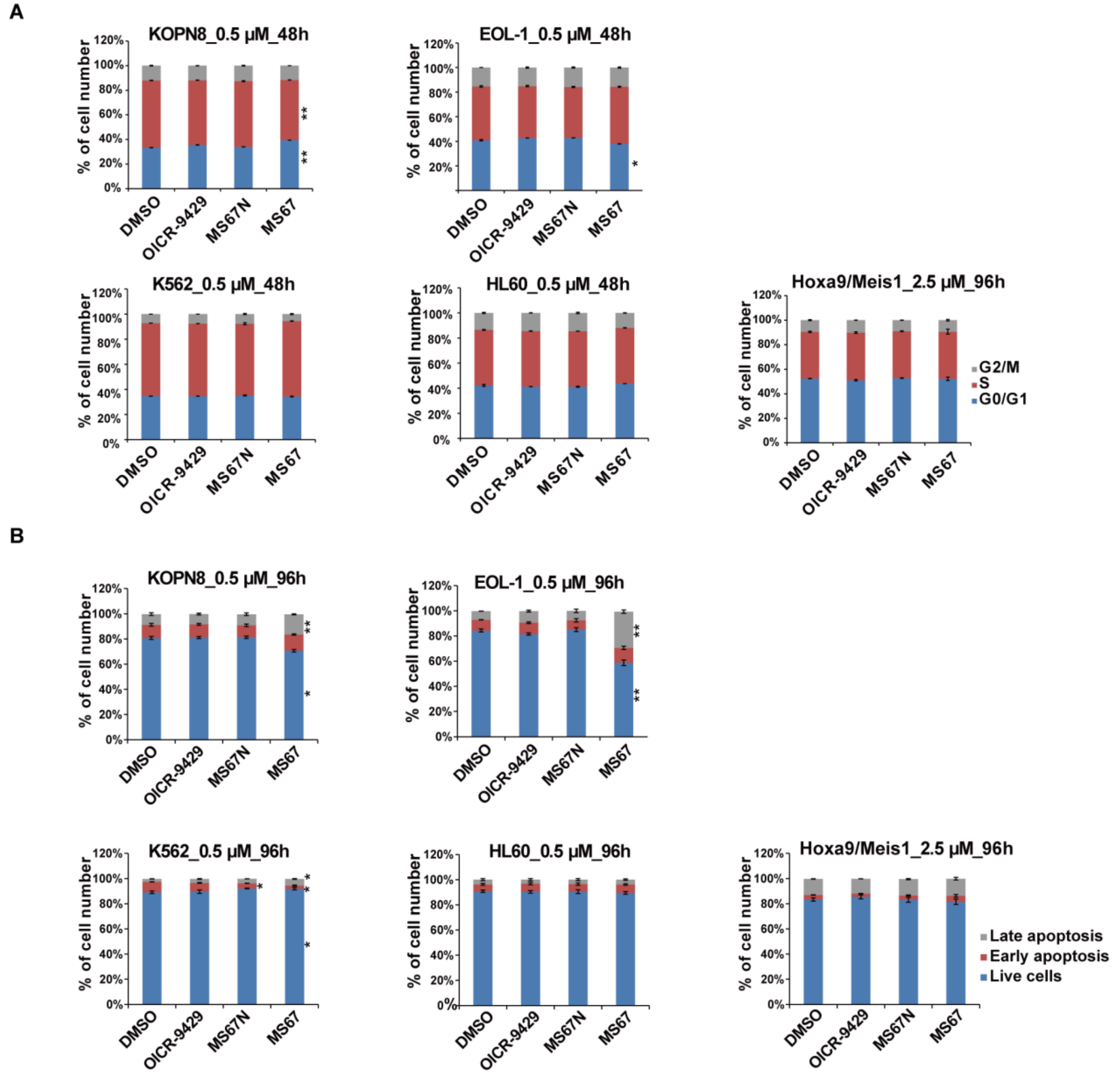

Fig. S18. MS67, but not MS67N or OICR-9429, induces cell cycle arrest and apoptosis in sensitive cell lines. Cell cycle progression (**A**; after 48 or 96 h treatment) and apoptosis analysis (**B**; 96 h treatment) using two sensitive cell lines (KOPN8 and EOL-1) and three insensitive cell lines (K562, HL60 and a murine AML line transformed by Hoxa9 plus Meis1). Cells were treated with DMSO, OICR-9427, MS67N or MS67 at indicated concentrations. Student's t test: *, $P < 0.05$; **, $P < 0.01$.

A

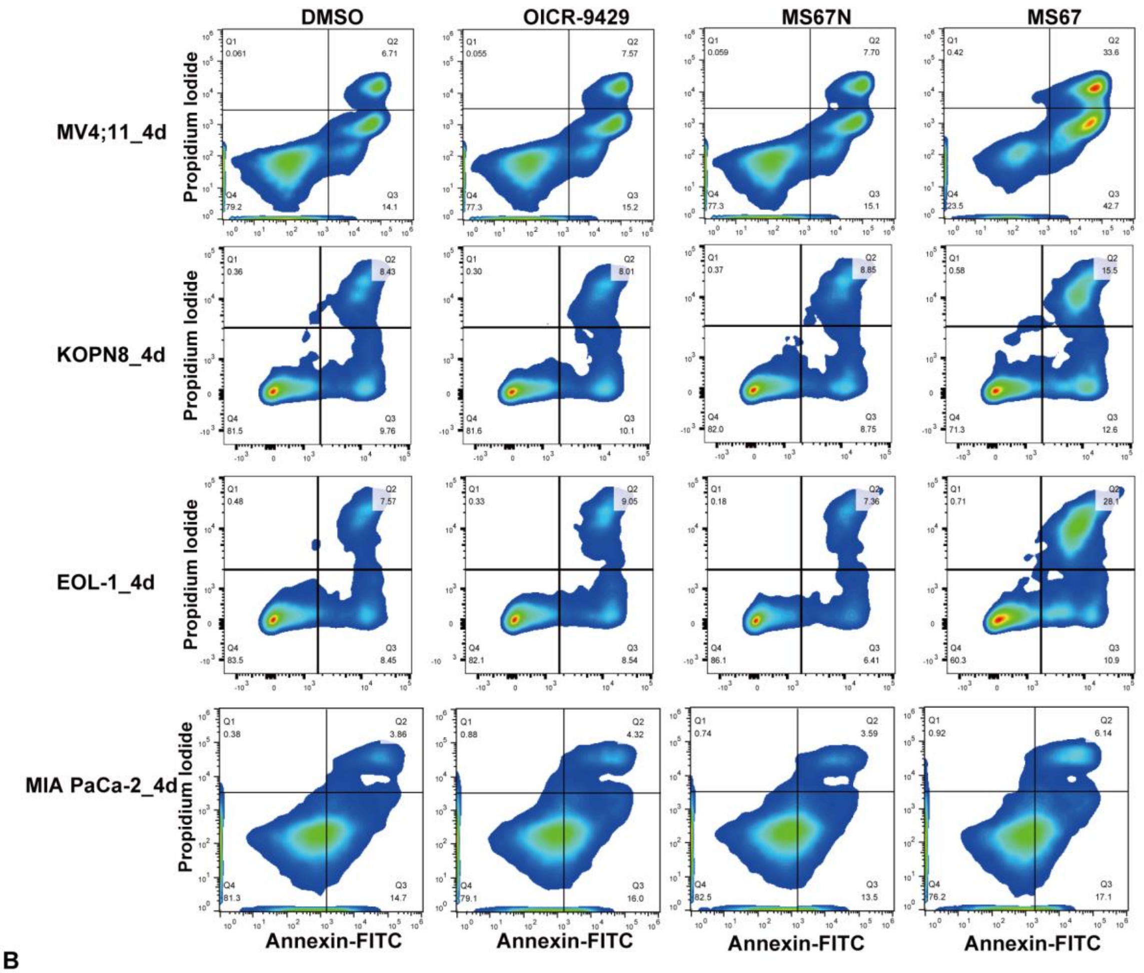
DMSO
OICR-9429
MS67N
MS67
MV4;11_4d
KOPN8_4d
EOL-1_4d
MIA PaCa-2_4d
Propidium Iodide
Annexin-FITC

B

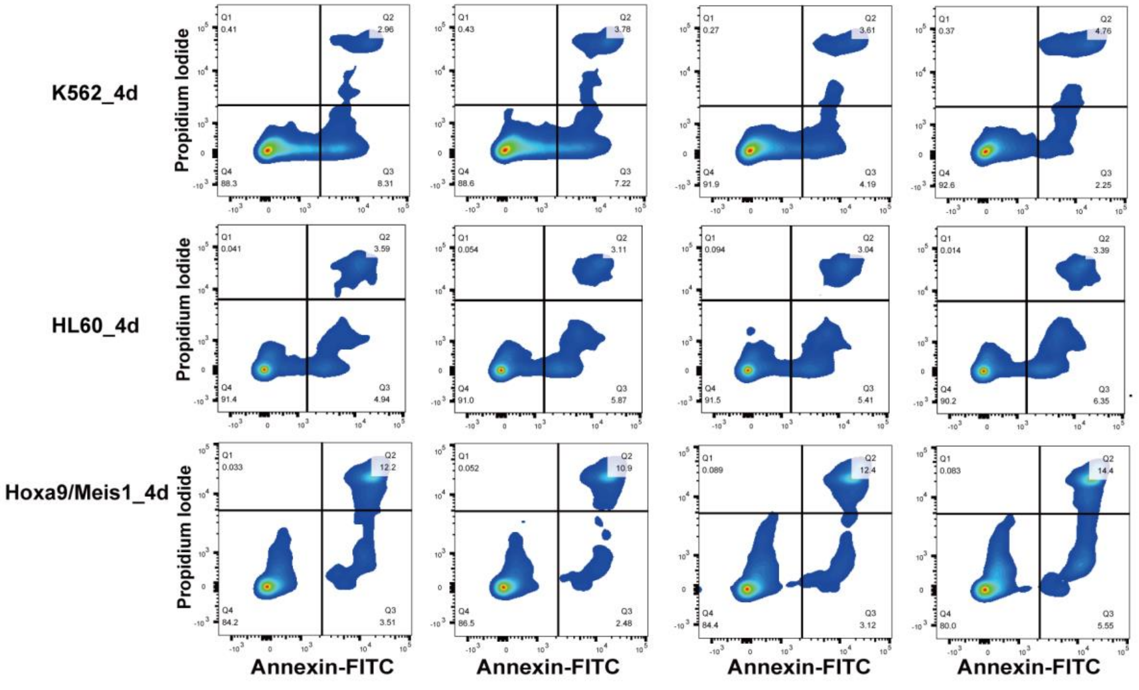
K562_4d
HL60_4d
Hoxa9/Meis1_4d
Propidium Iodide
Annexin-FITC

Fig. S19. Representative FACS scoring cell apoptosis in sensitive and insensitive cells. FACS scoring cell apoptosis of (**A**) sensitive cell lines (MV4;11, KOPN8, EOL-1 and MIA PaCa-2) and (**B**) insensitive cell lines (K562, HL60 and a murine AML line transformed by Hoxa9 plus Meis1). Cells were treated with DMSO or indicated concentrations of OICR-9429, MS67N or MS67 for 96 h.

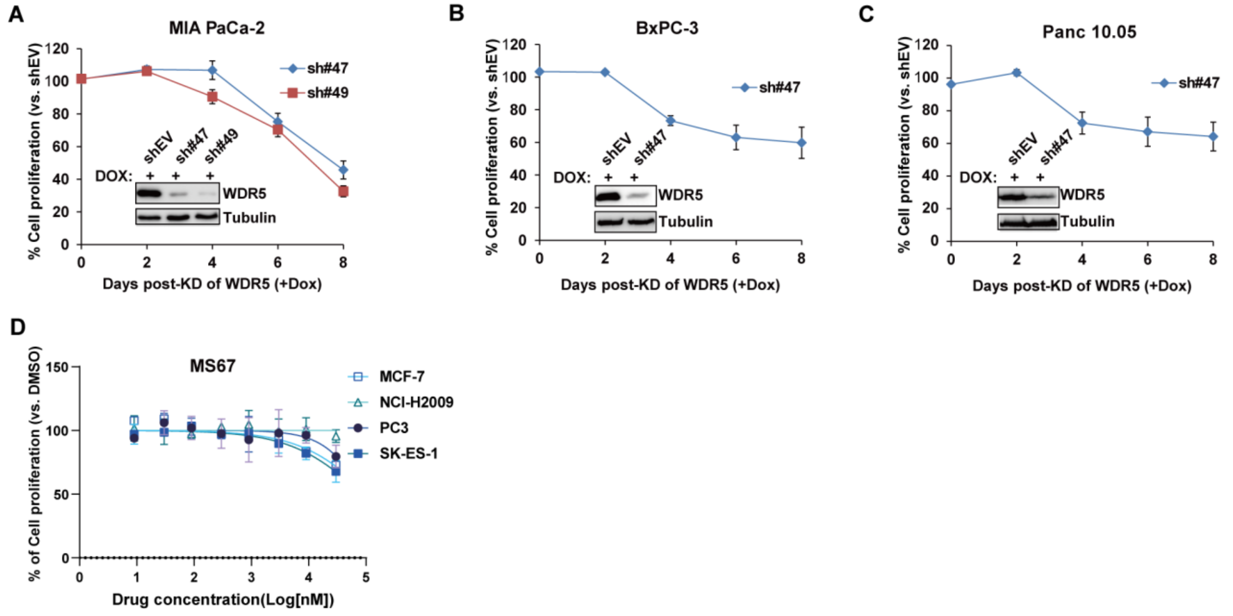

Fig. S20. The effect of WDR5 KD on cell growth inhibition in PDAC cells and the effect of MS67, MS67N and OICR-9429 on the growth in other cancer cell lines. (**A-C**) Cell proliferation of MIA PaCa-2 (**A**), BxPC-3 (**B**) and Panc 10.05 (**C**) cells bearing a WDR5-targeting shRNA (sh#47 or #49) or shEV, as determined by MTT assay. Immunoblots for WDR5 and Tubulin in MIA PaCa-2 (**A**), BxPC-3 (**B**) and Panc 10.05 (**C**) cells with stable expression of a doxycycline-inducible shRNA targeting WDR5 (sh#47 or #49) or shEV, after treatment with doxycycline (DOX+; 0.5 µg/mL) for 4 days. (**D**) Growth inhibitory curves of MS67 in MCF-7, NCI-H2009, PC3 and SK-ES-1 cells. Y-axis, presented in the mean ± SEM (n = 2), shows the relative cell number post-treatment with the indicated concentrations (x-axis) of MS67 for 3 days, normalized to DMSO-treated.

A

Sample ID	Age group	Material type	Diagnosis	Diagnosis phase	Genetic mutation	Blasts	Cytogenetics
172152	adult	BM	AML	New diagnosis	DNMT3A, RUNX1, STAG2,U2AF1	80%	47,XY,+6[9]/46,XY[11]
173685	adult	BM	AML	New diagnosis	CEBPA, TET2	75%	46,XX,i(7)(q10)[5]/46,XX[20]
182027	adult	BM	AML	New diagnosis	N/A	75%	446,XX,t(15;17)(q24;q21)[20]. PML/RARA results are positive. t(15;17)
172071	adult	BM	AML	Relapse or Refractory	FLT3-ITD, NPM1, DNMT3A	72%	46,XY,t(7;10)(p21;q22)[4]/46,XY[16]
PDX68555	adult	BM	AML	Relapse or Refractory	FLT3-ITD, MLL-AF9	93%	46,XX,t(9;11)(p22;q23)[3]

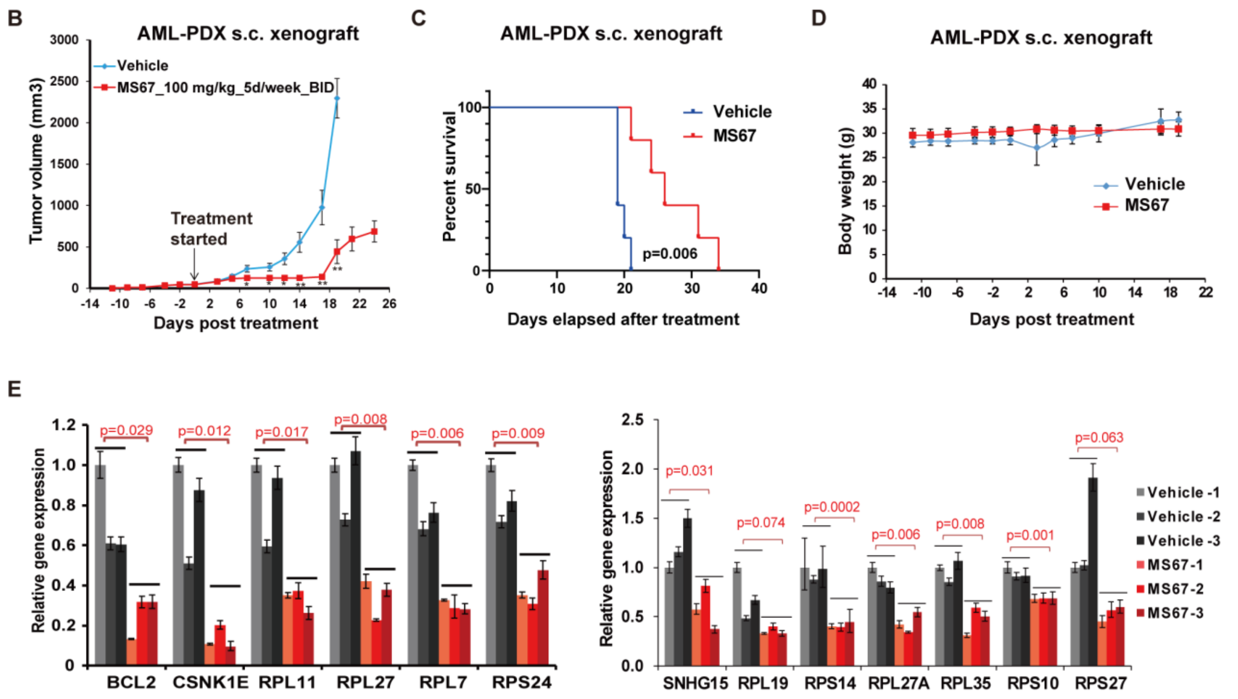

Fig. S21. MS67 suppresses the tumor growth, prolongs the survival, and downregulates WDR5 target genes and oncogenesis-related transcripts in an AML PDX model. (**A**) Characterization of the primary AML patient samples used in Fig. 8, **A** to **F**, and fig. S21, **B** to **E**. (**B**) The effect of MS67 on the growth of MLL-AF9+ AML PDX tumors xenografted subcutaneously (s.c.). Tumor bearing NSG-SGM3 mice were treated with vehicle (blue; n = 10) or 100 mg/kg of MS67 (red; n = 8, i.p. BID) for five days per week, starting at day 11 after inoculation. Y-axis shows the tumor volumes, measured every 2-3 days and presented in the mean ± SEM. *, *P* <0.05; **, *P* <0.01. (**C**) Kaplan-Meier curve of NSG-SGM3 mice xenografted with the MLL-AF9+ AML PDX tumors. Tumor bearing NSG-SGM3 mice were treated with vehicle (blue; n = 5) or 100 mg/kg of MS67 (red; n = 5, i.p. BID) for five days per week. (**D**) Body weights of NSG-SGM3 mice bearing the MLL-AF9+ AML PDX tumors, treated with vehicle (blue; n = 5) or 100 mg/kg of MS67 (red; n = 5, i.p. BID) for five days per week. (**E**) RT-qPCR of the indicated WDR5 target genes in tumor samples isolated from NSG-SGM3 mice bearing MLL-AF9+ AML PDX xenografts, treated with vehicle (gray; n = 3) or 100 mg/kg of MS67 (red; n = 3, i.p. BID).

Table S1. Data collection and refinement statistics. *Highest resolution shell is shown in parentheses.

	VCB-MS33-WDR5	VCB-MS67-WDR5
**Data Collection**		
Space group	$P2_1$	$P2_12_12_1$
Cell Dimensions		
a, *b*, *c* (Å)	47.4 187.8 49.2	63.8 98.6 127.6
α, β, γ (˚)	90 116.96 90	90 90 90
Resolution (Å)	93.92-1.70 Å (1.79-1.70)*	78.03-2.12 (2.23-2.12)*
Unique Reflections	83,681	46,520
R_{sym} or R_{merge} (%)	7.7 (40.5)	9.5 (56.7)
Rpim	4.7 (25.8)	3.8 (22.6)
Wilson B factor ($Å^2$)	24	33
$CC_{1/2}$	99.7 (50.6)	99.8 (82.1)
Completeness (%)	99.9 (100.00)	100 (100.00)
Redundancy	3.6 (3.3)	7.1 (7.2)
**Refinement**		
No. of Reflections	83,544	46,448
R_{work} (%)/ R_{free} (%)	20.0/22.6	19.1/22.4
No. of atoms		
Protein	5039	4971
Ligand	86	73
Water	646	439
Protein Residues	641	634
Average B-factors ($Å^2$)		
Protein	27	40
Ligand	27	27
Water	37	44
Ramachandran favored (%)	96.83	95.99
Ramachandran allowed (%)	3.17	4.01
Ramachandran outliers (%)	0.00	0.00
RMS Bonds (Å)	0.007	0.008
RMS Bond angles (˚)	1.21	1.21

Table S2. Selectivity of MS67 against 22 protein methyltransferases. Assays were performed in duplicate.

**Methyltransferase**	**% of Inhibition (10 μM)**
DOT1L	<10
EZH1 Complex	<10
EZH2 Complex	<10
G9a	<10
GLP	<10
NSD1	<10
NSD2	<10
NSD3	<10
PRMT1	<10
PRMT3	<10
PRMT4	<10
PRMT5/MEP50 Complex	<10
PRMT6	<10
PRMT7	<10
PRMT8	<10
SET1b Complex	<10
SET7/9	<10
SET8	<10
SETD2	<10
SMYD3	<10
SUV39H1	<10
SUV39H2	<10

Table S3. Selectivity of MS67 against 45 kinases. Assay results are shown as the mean ± SD from duplicate experiments.

**Target Name**	**% of Binding (1 μM)**
Abl	<10
Aurora-A	<10
CaMKIIα	<10
CDK1/cyclinB	<10
CDK2/cyclinA	<10
CHK1	22 ± 1
CHK2	<10
c-RAF	13 ± 5
cSRC	<10
EGFR	<10
EphA2	20 ± 6
EphA3	<10
EphB4	<10
FGFR1	<10
FGFR2	16 ± 1
FGFR3	<10
GSK3β	<10
IKKα	<10
IR	13 ± 1
IRAK4	<10
JAK3	<10
JNK1α1	<10
KDR	<10
Lck	<10
MAPK2	<10
MAP4K4	<10
MAPKAP-K2	<10
MARK1	<10
Met	12 ± 11
Mnk2	<10
NEK2	<10
PAK2	11 ± 2
PAK4	<10
PDK1	<10
Pim-2	<10
PKA	<10
PKBα	<10
PKCβII	<10
Plk1	13 ± 12
ROCK-I	<10
SAPK2a	<10
SGK	<10
SIK	16 ± 5
TAO2	<10
TRKA	<10

Table S4. Selectivity of MS67 against 44 GPCRs, ion channels, and transporters. Assay results are shown as the mean ± SD from quadruplicate experiments.

**Target Name**	**% of Binding (1 μM)**
5-HT1A	14 ± 13
5-HT1B	<10
5-HT1D	42 ± 5
5-HT1E	<10
5-HT2A	11 ± 7
5-HT2B	<10
5-HT2C	<10
5-HT3	26 ± 12
5-HT5A	36 ± 12
5-HT6	14 ± 8
5-HT7A	12 ± 8
Alpha1A	24 ± 20
Alpha1B	28 ± 10
Alpha1D	28 ± 6
Alpha2A	21 ± 7
Alpha2B	21 ± 7
Alpha2C	<10
Beta1	17 ± 21
Beta2	<10
Beta3	32 ± 12
BZP Rat Brain Site	42 ± 11
D1	19 ± 5
D2	25 ± 15
D3	23 ± 11
D4	<10
D5	22 ± 3
DAT	41 ± 22
GABAA	45 ± 3
H1	16 ± 6
H2	20 ± 3
H3	21 ± 19
H4	22 ± 7
KOR	26 ± 7
M1	44 ± 11
M2	31 ± 14
M3	24 ± 2
M4	31 ± 7
M5	<10
MOR	22 ± 7
NET	<10
PBR	22 ± 10
SERT	<10
Sigma 1	49 ± 3
Sigma 2	67 ± 10
